# Esophageal and Oropharyngeal Dysphagia: Clinical Recommendations From the United European Gastroenterology and European Society for Neurogastroenterology and Motility

**DOI:** 10.1002/ueg2.70062

**Published:** 2025-06-21

**Authors:** Amir Mari, Francesco Calabrese, Andrea Pasta, Greta Lorenzon, Bas Weusten, Jutta Keller, Pierfrancesco Visaggi, Sabine Roman, Elisa Marabotto, Ram Dickman, Jordi Serra, Nicola De Bortoli, Paola Iovino, Daniel Pohl, Dan Dumitrascu, Mentore Ribolsi, Claudia Barber, Serhat Bor, Mark Fox, Rami Sweis, Vicente Lorenzo‐Zuniga, Filiz Akyuz, Matteo Ghisa, Altay Celebi, Fahmi Shibli, Rainer Dziewas, Ismail Hakkı Kalkan, Jan Tack, Pere Clavé, Silvia Carrion, Ivy Cheng, Noemi Tomsen, Omar Ortega, Sergio Marin Rubio, Nicole Pizzorni, Emilia Michou, Julie Regan, Shaheen Hamdy, Nathalie Rommel, Martina Scharitzer, Olle Ekberg, Antonio Schindler, Renee Speyer, Anna Gillman, Frank Zerbib, Edoardo V. Savarino

**Affiliations:** ^1^ Gastroenterology Unit Nazareth EMMS Hospital Nazareth Israel; ^2^ The Azrieli Faculty of Medicine Bar Ilan University Ramat Gan Israel; ^3^ Gastroenterology Unit Department of Internal Medicine University of Genoa Genoa Italy; ^4^ IRCCS Policlinic San Martino Hospital Genoa Italy; ^5^ Department of Surgery, Oncology and Gastroenterology University of Padua Padua Italy; ^6^ Department of Gastroenterology St Antonius Hospital Nieuwegein the Netherlands; ^7^ Department of Gastroenterology University Medical Center Utrecht Utrecht University Utrecht the Netherlands; ^8^ Israelitic Hospital Hamburg Academic Hospital University of Hamburg Hamburg Germany; ^9^ Gastroenterology Unit Department of Translational Research and New Technologies in Medicine and Surgery University of Pisa Pisa Italy; ^10^ Digestive Endoscopy Unit Azienda Ospedaliero‐Universitaria Pisana Pisa Italy; ^11^ Digestive Physiology Hospices Civils de Lyon Hopital E Herriot Lyon I University LabTAU INSERM Lyon France; ^12^ Division of Gastroenterology Rabin Medical Center Beilinson Hospital Tel‐Aviv Israel; ^13^ Faculty of Medicine Tel Aviv University Tel Aviv Israel; ^14^ Digestive System Research Unit University Hospital Vall d’Hebron Barcelona Spain; ^15^ Centro de Investigación Biomédica en Red (CIBERehd) Barcelona Spain; ^16^ Gastroenterology Unit Department of Medicine, Surgery and Dentistry Scuola Medica Salernitana University of Salerno Salerno Italy; ^17^ Department of Gastroenterology University Hospital Zurich Zurich Switzerland; ^18^ Iuliu Hatieganu University of Medicine and Pharmacy Cluj County Clinical Emergency Hospotal Cluj‐Napoca Romania; ^19^ Unit of Gastroenterology and Digestive Endoscopy Campus Bio Medico University Rome Italy; ^20^ Division of Gastroenterology & Ege Reflux Team School of Medicine Ege University Izmir Turkey; ^21^ Digestive Function: Basel, Laboratory and Clinic for Motility Disorders and Functional Digestive Diseases Centre for Integrative Gastroenterology Arlesheim Switzerland; ^22^ Department of Gastroenterology and Hepatology University Hospital Zürich Switzerland; ^23^ GI Physiology Unit University College London Hospital London UK; ^24^ La Fe University and Polytechnic Hospital/IISLaFe Valencia Spain; ^25^ Catholic University of Valencia Valencia Spain; ^26^ Department of Gastroenterology Istanbul University Istanbul Faculty of Medicine Istanbul Turkey; ^27^ Department of Gastroenterology Faculty of Medicine Kocaeli University Kocaeli Turkey; ^28^ Institute of Gastroenterology and Hepatology Emek Medical Center Afula Israel; ^29^ Rappaport Faculty of Medicine Technion‐Israel Institute of Technology Haifa Israel; ^30^ Department of Neurology and Neurorehabilitation Klinikum Osnabrück – Academic Teaching Hospital of the University of Münster Osnabrück Germany; ^31^ Department of Neurology University Hospital Münster Münster Germany; ^32^ Department of Gastroenterology Türkiye Yüksek Ihtisas Training and Research Hospital Ankara Turkey; ^33^ Translational Research Center for Gastrointestinal Disorders University of Leuven Leuven Belgium; ^34^ Department of Gastroenterology and Hepatology UZ Leuven Leuven Belgium; ^35^ Gastrointestinal Physiology Lab Hospital de Mataró, Consorci Sanitari del Maresme Mataró Spain; ^36^ Centro de Investigación Biomédica en Red en Enfermedades Hepáticas y Digestivas (CIBEREHD) Instituto de Salud Carlos III Madrid Spain; ^37^ Academic Unit of Human Communication, Learning and Development Faculty of Education University of Hong Kong Hong Kong Hong Kong; ^38^ Division of Diabetes, Endocrinology, and Gastroenterology Centre for Gastrointestinal Sciences School of Medical Sciences University of Manchester Manchester UK; ^39^ Department of Biomedical and Clinical Sciences Università degli Studi di Milano Milan Italy; ^40^ Department of Speech Language Therapy School of Health Rehabilitation Sciences University of Patras Patras Greece; ^41^ Department of Clinical Speech & Language Studies Trinity College Dublin Dublin Ireland; ^42^ Division of Diabetes, Endocrinology and Gastroenterology GI Sciences School of Medical Sciences Faculty of Biology, Medicine and Health University of Manchester Manchester UK; ^43^ Department of Neurosciences ExpORL Deglutology University of Leuven Leuven Belgium; ^44^ Department of Gastroenterology, Neurogastroenterology and Motility University Hospitals Leuven Leuven Belgium; ^45^ Department of Biomedical Imaging and Image‐Guided Therapy Medical University of Vienna Vienna Austria; ^46^ Department of Translational Medicine/Radiology Lund University Malmö Sweden; ^47^ School of Health Sciences College of Medicine, Nursing & Health Sciences University of Galway Galway Ireland; ^48^ Speech and Language Therapy Department Tallaght University Hospital Dublin Ireland; ^49^ Department of Gastroenterology CHU de Bordeaux Centre Médico‐Chirurgical Magellan Hôpital Haut‐Lévêque INSERM CIC 1401 Université de Bordeaux Bordeaux France; ^50^ Gastroenterology Unit Azienda Ospedale Università di Padova Padua Italy

**Keywords:** barium swallow, Delphi consensus, diagnosis, dysphagia, esophageal motility disorders, high resolution manometry (HRM), management, oropharyngeal dysphagia, workup

## Abstract

Dysphagia is a prevalent symptom of the upper gastrointestinal tract causing health related consequences, impacting quality of life and is associated with global economic burden. Swallowing difficulties are classified into oropharyngeal dysphagia (OD) and esophageal dysphagia. Despite its clinical importance, dysphagia is associated with several uncertainties regarding its optimal diagnostic work‐up and management, particularly, considering the progress with diagnostic modalities and technologies. A Delphi consensus was performed with experts from various disciplines who conducted a literature summary and voting process on 41 statements. Quality of evidence was evaluated using the grading of recommendations, assessment, development, and evaluation criteria. Consensus was reached for all the statements. The panel agreed with the definition and prevalence of esophageal and OD types. The role of endoscopy, high‐resolution manometry, EndoFLIP, barium swallow and other imaging tests in evaluating esophageal dysphagia has reached overall strong agreement. Videofluoroscopic swallow study, alongside fiber‐endoscopic evaluation of swallowing, as the methods of choice for the instrumental assessment of oropharyngeal dysfunction is a strong recommendation. Regarding treatment, a weak recommendation was achieved for the use of PPIs, calcium‐channel blockers, nitrates, phosphodiesterase type 5 inhibitors, antidepressants or peppermint oil for the treatment of hypercontractile esophagus. A strong recommendation exists for endoscopic and surgical treatment of achalasia, while a weak recommendation is provided for other esophageal motility disorders. Regarding OD, a weak recommendation was achieved for swallow therapy, to improve swallowing mechanics, reduce symptoms, and enhance quality of life. Swallow therapy could be more effective when using validated assessment tools, consistent treatment parameters, and considering long‐term follow‐up. A multinational group of European experts summarized the current state of consensus on the definition, diagnosis, and management of dysphagia.

## Graphical abstract

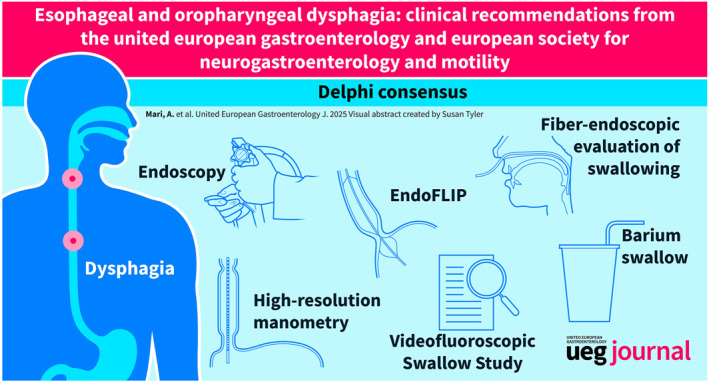

**Abbreviations**
AI‐EoE‐EREFS: Artificial Intelligence‐based Eosinophilic Esophagitis Endoscopic Reference Score
DBS: deep brain stimulation
EGJ: esophagogastric junction
EGJ‐DI: esophagogastric junction distensibility index
EGJOO: esophagogastric junction outflow obstruction
EoE: eosinophilic esophagitis
ESNM: European Society for Neurogastroenterology and Motility
EUS: endoscopic ultrasonography
FEES: fiberoptic endoscopic evaluation of swallowing
FLIP: functional lumen imaging probe
GERD: gastroesophageal reflux disease
HRM: high‐resolution manometry
IRP: integrated relaxation pressure
LES: lower esophageal sphincter
MBS: modified barium swallow
MRI: magnetic resonance imaging
MRS: multiple rapid swallows
NMES: neuromuscular electrical stimulation
OD: oropharyngeal dysphagia
PES: pharyngeal electrical stimulation
PHRM: pharyngeal high‐resolution manometry
PMS: peripheral magnetic stimulation
POEM: peroral endoscopic myotomy
RDC: rapid drink challenge
rTMS: repetitive transcranial magnetic stimulation
TBE: timed barium esophagram
tDCS: transcranial direct current stimulation
UEG: United European Gastroenterology
UES: upper esophageal sphincter
VFSS: videofluoroscopic swallow study

## 1 Introduction

Dysphagia is a prevalent upper gastrointestinal symptom, manifesting as a swallowing dysfunction, which has a substantial impact on patient quality of life [1]. This condition can involve patients of any age, but its prevalence is particularly common among elderly individuals and patients with neurological disorders. However, in recent years the prevalence of dysphagia is rising among young adults, primarily due to an increase in diagnosis such as gastroesophageal reflux disease (GERD) and eosinophilic esophagitis [2, 3].

The primary distinction in dysphagia is based on the anatomical region involved, whether esophageal or oropharyngeal [3]. Each of these disease categories impacts the swallowing process through different mechanisms resulting in distinct clinical challenges that require different diagnostic approaches, and tailored management strategies. This complexity underscores the pivotal relevance of a systematic and rigorous diagnostic approach, guiding the development of optimal, evidence‐based management strategies that target the specific mechanisms underlying dysphagia [4].

Technological innovation has driven improvements in dysphagia management with substantial progress witnessed in diagnostic modalities as well as new therapeutic options. High‐resolution manometry (HRM) is now the gold‐standard method for the assessment of esophageal peristalsis and the function of the EGJ [5]. Meaningfully, the evolution of HRM has permitted the introduction of the Chicago Classification (CC), which is the accepted working algorithm for analyzing and interpreting HRM studies. The most recent iteration of the CC divides esophageal motility disorders into disorders of the esophago‐gastric junction (such as achalasia, esophagogastric outflow obstruction) and of esophageal peristalsis (such as esophageal spasm, ineffective peristalsis) [6]. More recently, the endoluminal functional lumen imaging probe (EndoFLIP) has been introduced and implemented in clinical practice. EndoFLIP is an innovative technology, performed under sedation, to assess function of the esophageal body and esophagogastric junction by using a special balloon designed to measure diameter and distensibility, respectively [7, 8]. Esophageal imaging, such as the video fluoroscopic swallowing exam (VFSE), timed barium swallow (TBS), and timed barium surface area measurement, further enrich the diagnostic opportunities when investigating dysphagia.

The effective management of dysphagia demands substantial healthcare resources, encompassing specialized diagnostic evaluations, therapeutic interventions, and often long‐term care for high‐risk patients [9]. Given this knowledge, the United European Gastroenterology (UEG) and the European Society for Neurogastroenterology and Motility (ESNM) recognized the need to develop updated clinical guidelines. These guidelines aim to raise awareness of dysphagia, support clinicians in accurately diagnosing and managing these disorders, and ultimately improve patient outcomes.

## 2 Methods

In collaboration with other European societies, the UEG and ESNM have initiated a Delphi process to formulate consensus statements on various aspects of dysphagia. This approach combines evidence‐based medicine with systematic literature reviews and a structured voting procedure to reach consensus on complex medical topics, particularly when evidence from randomized controlled trials is limited.

The first step of this process was the selection of a six‐member Working Group from the ESNM, each with extensive expertise in gastroenterology, esophageal motility disorders and oropharyngeal conditions, and/or Delphi methodology. Then, the Working Group identified 41 clinical questions using the PICO (Patient, Intervention, Comparator, Outcome) framework to guide each question and allow the selection of a European Consensus Group consisting of gastroenterology and motility experts, ear‐nose‐throat specialists and methodologists from 10 European countries, nominated by the ESNM board and UEG‐associated societies, including the Società Italiana di Gastroenterologia ed Endoscopia (SIGE), Romanian Society of Gastroenterology & Hepatology (RSGH), Turkish Society of Gastroenterology (TSG), Israeli Association of Gastroenterology (IGA), European Society of Gastrointestinal Endoscopy (ESGE), European Society for Clinical Nutrition and Metabolism (ESPEN), European Society of Gastrointestinal and Abdominal Radiology (ESGAR), European Society of Swallowing Disorders (ESSD). The Consensus Group ESNM and UEG board members selected a total of 40 experts with different background in gastroenterology, neurology, radiology, speech therapy, surgery, and ear‐nose‐throat disorders to ensure multidisciplinary perspectives. Table S1 reports all questions and search methodologies.

A systematic literature review has been conducted for each question using MEDLINE, EMBASE, and the Cochrane Database, with all sources and references accessible to participants, until 1st October 2024. Two rounds of voting were required to refine and approve the final statements. During each round, statements were rated on a 6‐point Likert scale (Table 1), with consensus defined as at least 80% agreement. Feedback from participants guided revisions and additional question development were also collected and integrated in the final draft.

**TABLE 1:** Six‐point Likert scale.

Point description
A+ agree strongly
A agree with minor reservation
A− agree with major reservation
D− disagree with major reservation
D disagree with minor reservation
D+ disagree strongly

The recommendations were divided into two categories based on the strength of the evidence according to GRADE methodology (https://www.gradeworkinggroup.org). Strong recommendations indicate high or moderate‐quality evidence, suggesting that the benefits clearly outweigh the risks (for) or vice versa (against), and should be applied in most patients. In cases where evidence was insufficient or the application of GRADE was not appropriate, we adhered to the good practice statement guidance established by the GRADE Working Group. The Guidelines International Network (GIN) group outlines specific criteria for issuing good practice statement when formal grading is unsuitable. Statements that did not fully meet these criteria were explicitly labeled as “Expert opinion,” whereas those that satisfied the requirements without employing GRADE were classified as “Good Practice Statements” [10]. Specifically, statements were classified as GPS only if they met all five GRADE‐defined criteria: (1) the statement is necessary for healthcare practice; (2) implementing the statement would result in large net positive consequences after consideration of all relevant outcomes and downstream effects; (3) conducting a systematic collection and summary of indirect evidence would be an inefficient use of the guideline panel's limited time and resources; (4) a clear and explicit rationale connecting the statement to indirect evidence is provided; and (5) the statement is clear and actionable. Statements not meeting all these explicit criteria were labeled as “Expert opinion” [10].

The quality of the evidence was rated on a scale. High‐quality evidence meant there was strong confidence in the findings, with little likelihood that future research would alter the results. Moderate‐quality evidence indicated a fair level of confidence, though further studies could potentially impact the conclusions. When the evidence was low in quality, confidence in the findings was more limited, and additional research could change the results. Finally, very low‐quality evidence suggested minimal confidence in the findings due to serious limitations in the data. Additional considerations included the balance of benefits and risks, patient preferences, and resource utilization to ensure practical relevance and applicability.

In August 2024, the Consensus Group completed the initial voting, and statements were revised before a final voting round (October 2024). After achieving consensus, the statements were compiled, reviewed and approved by all participants, and finalized for publication. These guidelines represent a collaborative effort to provide evidence‐based recommendations and support clinicians in dysphagia management, aiming to enhance patient outcomes and optimize clinical practices across Europe.

## 3 Results

### 3.1 Section 1: Definition and Epidemiology

Statement 1.1 UEG/ESNM propose the following definition of esophageal dysphagia: a difficulty swallowing generally sensed retrosternally and related to the esophagus or its lower esophageal sphincter (LES) caused by anatomical, mechanical or functional abnormalities.

Quality of evidence: very low; recommendation: expert opinion

Statement endorsed, overall agreement: 87% (A+ 69%, A 18%, A− 8%, D− 0%, D 5%, D+ 0%)

**Summary of evidence**

A universal consensus on the definition of dysphagia has been difficult to establish. In published papers, no uniform, standardized definition has been applied and different health organizations have defined esophageal dysphagia variously. Generally, esophageal dysphagia referred to swallowing impairment originating from the esophagus [11], characterized by sensation of retrosternal food sticking [12]. Dysphagia is classified under “symptoms and signs” in ICD‐10 and is simply defined as difficulty in swallowing [13]. According to the World Gastroenterology Organization (WGO), esophageal dysphagia refers to the sensation that foods and/or liquids are somehow being obstructed in their passage from the mouth to the stomach (usually described as “esophageal dysphagia”) [14]. The European Society for Swallowing Disorders has recently published a White Paper on screening and non‐instrumental assessment of dysphagia for adults [15]. The used definition of dysphagia in this paper was: dysphagia is the dysfunction of one or more parts of the swallowing apparatus, including the mouth, tongue, oral cavity, pharynx, airway, and finally esophagus and its upper and lower sphincters, often due to anatomical or structural deficiencies or abnormalities [16]. In a recent modified multi‐professional Danish Delphi study aimed at establishing an acceptable definition of dysphagia for application across specialities and sectors to enhance collaborations [17], the definition that was widely acceptable by most healthcare professionals who participated in the study was: “Dysphagia is a functional impairment that either prevents or limits the intake of food and fluids, and which makes swallowing unsafe, inefficient, uncomfortable or affects quality of life.” In this study, no particular definition to esophageal dysphagia was given. In summary, a definition that clearly distinguishes between oro‐pharyngeal and esophageal dysphagia has not been established. To address this, the UEG guidelines propose the following definition of esophageal dysphagia: a difficulty swallowing generally sensed retrosternally and related to the esophagus or its LES caused by anatomical, mechanical or functional abnormalities.

Statement 1.2 UEG/ESNM recognize that OD is defined as a swallowing dysfunction that causes impairment in moving the bolus safely and effectively from the mouth through the pharynx into the esophagus. The main signs are divided into safety (airway penetration and aspiration) or efficacy (prolonged swallow response, impaired labial seal, premature spillage in the pharynx, piecemeal deglutition, nasal regurgitation, oral or pharyngeal residue and impaired upper esophageal sphincter opening) swallowing impairments. The main accompanying symptoms include cough, choking, fear of eating, voice change, globus, prolonged meal duration, fatigue, and reduced pleasure of eating.

Quality of evidence: very low; recommendation: expert opinion

Statement endorsed, overall agreement: 88% (A+ 73%, A 15%, A− 5%, D− 2%, D 5%, D+ 0%)

**Summary of evidence**

OD refers to any impairment in the oral preparatory, oral propulsive and/or pharyngeal phases of swallowing, resulting in the difficulty or inability to move an alimentary bolus from the mouth to the esophagus [3, 18]. OD can include oropharyngeal aspiration and is classified as a health problem under “digestive symptoms and signs” in the International Classification of Diseases (ICD) also promoted by the WHO [13].

There are several definitions of OD in the literature. Two classical ones are:

1. OD results from either oropharyngeal swallowing dysfunction or perceived difficulty in the process of swallowing. Major categories of swallowing dysfunction are (a) an inability or excessive delay in initiation of pharyngeal swallowing, (b) aspiration of ingestate, (c) nasopharyngeal regurgitation, and (d) residue of ingestate within the pharyngeal cavity after swallowing and (e) impaired upper esophageal sphincter (UES) function [19].
2. OD is a symptom of a swallow dysfunction that provokes difficulty or inability to form or move the alimentary bolus safely from the mouth to the esophagus. It can include oropharyngeal aspiration (the entry of secretions, food, or drink from the oro‐pharynx into the trachea or the lungs) and choking (the subsequent mechanical obstruction of pulmonary air flow) [20].

Signs of OD can be divided into signs of impaired safety and signs of impaired efficacy of swallowing [21, 22]. Signs of impaired safety refer to airway penetration and aspiration. Signs of impaired efficacy include prolonged oral or pharyngeal phases, anterior spillage of the bolus due to impaired labial seal, premature spillage in the pharynx, piecemeal deglutition, nasal regurgitation, oral residue, pharyngeal residue and impaired UES opening.

Patients with OD can experience a wide variety of digestive and respiratory symptoms. Most commonly reported symptoms are: coughing during eating or drinking, choking, fear of eating/choking, change in vocal quality when eating or drinking (typically wet/gurgly voice), sensation of food sticking in the throat, feeling of residue in the pharynx, prolonged meal duration, fatigue at meals, diet restriction to specific foods or consistencies, and reduced pleasure of eating [16, 23].

Normal swallowing requires the close functional coordination of the mouth, pharynx, and esophagus, and if one of these components becomes functionally impaired, it is likely that the others may be affected [24]. There is evidence of OD in non‐obstructive esophageal dysphagia and in children with eosinophilic esophagitis, but more studies are needed to explore this relationship [25].

Statement 1.3 UEG/ESNM recognizes that the general prevalence of dysphagia is estimated to be between 10% and 20%, depending on the definition used and the population included. Notably, no studies have assessed esophageal dysphagia prevalence as a separate concrete entity, therefore it is not possible to estimate its true prevalence.

Quality of evidence: very low; recommendation: expert opinion

Statement endorsed, overall agreement: 87% (A+ 50%, A 37%, A− 3%, D− 8%, D 2%, D+ 0%)

**Summary of evidence**

The prevalence of dysphagia is hard to estimate due to the use of different definitions of dysphagia across the various studies, inclusion of heterogeneous populations and the application of various tools or questionnaires. Notably, to the best of our knowledge, no subclassification to OD and esophageal dysphagia was performed in epidemiological studies. According to the limited data collected and with the aforementioned limitations, the estimated prevalence of dysphagia in the general population could be around 20%, increasing with age [26]. Wilkins et al. reported that 22.6% of the USA general population experienced dysphagia at least several times per month and that the prevalence was higher among the elderly population [27]. Adkins et al. conducted a population‐based survey of over 31,000 USA adults to evaluate the epidemiology of dysphagia and reported a prevalence of 16% [1]. Despite the lack of subclassification into OD and esophageal dysphagia, 40.6% of respondents reported being diagnosed with at least one esophageal disorder (most often Gastro‐Esophageal Reflux Disease) [1]. This survey highlighted that dysphagia is common, affecting around 1 out of 6 of adults at some point during their lives. Comparable dysphagia prevalence was reported among Australian [28] and South American [29] populations, whereas lower prevalence rates of dysphagia were reported in Asian populations [30, 31]. On the other hand, when considering only the diagnoses of functional dysphagia, the prevalence significantly decreases, ranging between 1.2% and 3.2%, depending on the population studied [32].

To summarize, the global prevalence of dysphagia is challenging to evaluate but can be estimated to be between 10% and 20%. Notably, these prevalence estimates are further limited by the heterogeneity in study methodologies, variations in diagnostic criteria, and the lack of standardized subclassification between oropharyngeal and esophageal dysphagia, making direct comparisons and accurate epidemiological assessments challenging.

Statement 1.4 UEG/ESNM recognize that the prevalence of OD varies according to the target population. Elderly patients, patients with neurological or neurodegenerative diseases, and patients with head or neck diseases have the highest prevalence.

Level of evidence: moderate, grade of recommendation: good practice statement

Statement endorsed, overall agreement: 90% (A+ 76%, A 14%, A− 6%, D− 2%, D 0%, D+ 2%)

**Summary of evidence**

The prevalence of OD increases with age, and the main populations at risk of OD are elderly patients, patients with neurological or neurodegenerative diseases, and patients with head or neck diseases [3]. In a recent meta‐analysis investigating the prevalence of OD in a sample of more than 9000 individuals with different phenotypes, the overall estimated global prevalence was 43.8% (95% CI, 33.3%–54.9%) [33]. However, there was significant fluctuation in the overall prevalence of OD according to different populations and diagnostic tools. A systematic review with meta‐analysis reported an overall prevalence of OD in the general population was 13.4% (95% CI, 4.4–34.5), while the prevalence was 31.5% (95% CI, 8.9–68.4) in patients with head or neck cancer, 48.1% (95% CI, 31.9–64.7) in elderly people, 55.4% (95% CI, 37.2–72.2) in patients with previous stroke, and up to 72.4% (95% CI, 26.7–95.0) in patients with dementia [33]. Specifically in elderly patients, another systematic review conducted on 31,488 adult patients older than 60 years, estimated a pooled prevalence of OD of 42% [34], but with different prevalence according to the target populations, with a prevalence of 23% in community older patients, between 29.4% and 47% in hospitalized patients, but increasing to 75%–91% in elderly patients admitted to the hospital with a community‐acquired pneumonia, and between 40% and 51% in nursing home residents [35]. Another meta‐analysis including 26,366 acute post‐stroke patients estimated a pooled OD prevalence of 42% (95% CI, 37%–48%) [36], while in another meta‐analysis, the prevalence of OD was 36.9% (95% CI: 30.7%–43.6%) in patients with Parkinson's disease [37], and in a systematic review was between 27% and 30% in patients with brain injury [38]. Dysphagia is also prevalent among patients with idiopathic inflammatory myopathies. A systematic review and meta‐analysis which included 10,382 patients found that the overall prevalence of dysphagia among patients with IIM is 36% (95% CI, 33%–40%) with the highest prevalence in patients with inclusion body myositis: 56% (95% CI, 47%–65%) [39].

Recently, it was also shown that the global prevalence of OD varied according to the tool used for the diagnosis, and was 20.4% (95% CI, 9.6–38.4) based on swallow questionnaires, 40.9% (95% CI, 26.3–57.3) based on physical examination, and 54.4% (95% CI, 39.2–68.8) according to the volume‐viscosity swallow test [33]. Using instrumental explorations further increases the prevalence of the condition, increasing the prevalence of OD in the chronic phase of stroke to 45%–81% with instrumental assessment [35].

Statement 1.5 UEG/ESNM recognize that OD is associated with higher hospital stays and costs as well as higher probability of being transferred to post‐acute care facilities such as nursing homes. Moreover, the development of nutritional and respiratory complications, such as malnutrition and respiratory infections, has been associated with an independent increase in long‐term costs for follow‐up, especially in post‐stroke patients.

Level of evidence: moderate, recommendation: good practice statement

Statement endorsed, overall agreement: 95% (A+ 71%, A 24%, A− 3%, D− 0%, D 0%, D+ 2%)

**Summary of evidence**

Recent literature shows that OD is linked to longer hospital stays, higher costs, and an increased likelihood of transfer to post‐acute care facilities. OD‐related complications, such as malnutrition and respiratory infections, further increase healthcare resource consumption [40].

An observational study in hospitalized U.S. patients showed OD significantly longer hospital stays and higher costs (length of hospital stay was 3.8 days longer, and total inpatient costs were $6243 higher), with a 33.2% higher probability of transfer to post‐acute care facilities [41]. A systematic review and meta‐analysis showed OD secondary to different etiologies increased hospital stay by 2.99 days and costs by 40.36% [42].

OD's economic impact has been primarily evaluated in stroke patients. A prospective U.S. study found higher mean hospitalization costs for those dependent on tube feeding compared with non‐tube‐fed patients ($12,538 vs. $5949; *p* < 0.0001) [43]. A retrospective study found the 1‐year attributable cost of OD after ischemic stroke to Medicare was $4510, increasing in‐hospital costs by 23% and length of hospital stay by 30% [44]. European studies also identified post‐stroke OD as a risk factor for longer hospital stays and the need for discharge to nursing homes [45].

A prospective study in Catalonia showed OD resulted in an independent and significant cost increase of 789.68 euros during hospitalization and significantly higher mean costs of around 12,500 euros for those patients who developed post‐stroke OD, were at risk or malnourished and had at least one episode of respiratory infection compared with those who did not develop OD at 12 months [46]. A retrospective study in Germany found severe OD as an independent predictor of increased health insurance expenditures [47]. Systematic reviews indicate increased hospital stay lengths for stroke patients with OD and suggest cost‐effectiveness for interventions aiming to prevent OD complications [40, 42, 48].

The health economic impact of OD has also been assessed in geriatric populations. A retrospective study in Denmark found 1‐year significantly higher in‐hospital (3677 euros) and municipality costs (6192 euros) for geriatric patients with OD after acute hospitalization [49]. In Canada, hospitalized patients with Parkinson's disease and OD had 44% longer hospital stays and 46% higher inpatient costs [50].

Given the chronic nature of OD, it is reasonable to assume that costs associated with OD encompass in‐hospital costs after acute events to chronic care costs in ambulatory settings, rehabilitation facilities, socio‐sanitary care institutions, or nursing homes. Programs based on early detection and prevention of OD‐related complications could be cost‐effective [40, 42, 46, 48].

Focusing on esophageal dysphagia, the available data are more limited and often do not permit a separate analysis of this condition apart from OD. From a national database analysis in US of around 77 million hospital admissions it was found that dysphagia, regardless of whether it is oropharyngeal or esophageal, is linked to prolonged hospital stays (from 2.4 to 4.04 days), higher inpatient costs, and increased comorbidities and consequent mortality [51]. Overall, dysphagia impacts healthcare costs extensively, with an average increase of $6243 in hospital care for those diagnosed compared to those without it [41]. Furthermore, patients with dysphagia were 33.2% more likely to be transferred to post‐acute care facilities, and in‐hospital mortality rates were 1.7 times higher compared to non‐dysphagia cases [41]. The psychosocial impact, particularly among the elderly population, is significant, with many avoiding social interaction and meals due to dysphagia‐related anxiety [52].

### 3.2 Section 2: Physiopathology and Related Conditions

Statement 2.1 UEG/ESNM recognize that a wide range of systemic neuromuscular, rheumatological/immunological, endocrinological, and infectious diseases can be associated with esophageal and/or OD.

Level of evidence: moderate, recommendation: good practice statement

Statement endorsed, overall agreement: 82% (A+ 69%, A 13%, A− 15%, D− 3%, D 0%, D+ 0%)

**Summary of evidence**

A wide range of systemic diseases can lead to esophageal and/or OD, spanning various categories including neuromuscular, rheumatological/immunological, endocrinological, and infectious conditions [53]. Each of these disease categories impacts the swallowing process through different mechanisms, resulting in significant clinical challenges and requiring tailored management strategies [28, 53]. Table 2 reports the main disease and medical disorders associated with dysphagia.

*Neurologic and neuromuscular diseases:* Esophageal and OD can result from several neuromuscular disorders. Multiple sclerosis, Parkinson's disease, and amyotrophic lateral sclerosis are significant contributors [54]. Additionally, conditions like myasthenia gravis and polymyositis/dermatomyositis specifically affect the oropharyngeal region due to their impact on muscle strength and coordination [55, 56].

*Rheumatological and immunological diseases:* Systemic sclerosis and rheumatoid arthritis are important rheumatological disorders that can cause esophageal dysphagia due to fibrosis and inflammation of the esophagus [57, 58]. Sjogren syndrome and systemic lupus erythematosus are associated with OD, likely due to their effects on salivary and other exocrine glands, leading to dry mouth and swallowing difficulties [59]. Pemphigus and sarcoidosis are immunological diseases that can cause dysphagia in both the esophageal and oropharyngeal regions due to mucosal involvement and granulomatous inflammation, respectively [60]. Eosinophilic esophagitis (EoE), characterized by eosinophilic infiltration, specifically affects the esophagus [2].

*Endocrinological diseases:* Diabetes mellitus and hypothyroidism are endocrinological conditions that can lead to dysphagia. In diabetes, autonomic neuropathy can impair esophageal motility, while hypothyroidism can lead to muscle weakness and reduced gastrointestinal motility, affecting both esophageal and oropharyngeal swallowing processes [61].

*Infectious diseases:* Various infections can result in dysphagia. COVID‐19, candidiasis, and herpes simplex virus (HSV) can cause esophageal dysphagia. While COVID‐19, candidiasis, Epstein–Barr virus (EBV) infection, and botulism can lead to OD. These infections can directly damage the mucosa or affect the neural control of swallowing [62, 63].

*Congenital/developmental:* Congenital conditions like esophageal atresia, tracheoesophageal fistula, and esophageal webs can cause dysphagia by altering the esophageal structure from birth. Developmental disorders such as cerebral palsy contribute to OD due to impaired muscle coordination [64].

*Structural/mechanical:* Structural causes like esophageal strictures, rings, webs, and diverticula obstruct food passage, leading to dysphagia. GERD can result in peptic strictures, while hiatal hernias and Zenker's diverticulum affect normal swallowing mechanics [65].

*Oncological:* Esophageal and oropharyngeal cancers, such as squamous cell carcinoma, obstruct swallowing pathways. Cancer treatments, including surgery and radiation, often result in scarring or nerve damage, contributing to dysphagia [66].

**TABLE 2:** Diseases and medical disorders associated with dysphagia.

Category	Diseases associated with oropharyngeal dysphagia	Diseases associated with esophageal dysphagia
Neurologic/neuromuscular	Stroke, multiple sclerosis, Parkinson's disease, amyotrophic lateral sclerosis, myasthenia gravis, polymyositis/dermatomyositis, Huntington disease	Multiple sclerosis, Parkinson's disease, amyotrophic lateral sclerosis
Rheumatological	Sjogren syndrome, systemic lupus erythematosus	Systemic sclerosis, rheumatoid arthritis
Other immunological	Pemphigus, sarcoidosis	Pemphigus, EoE, sarcoidosis, lichen planus
Endocrinological	Diabetes mellitus, hypo/hyperthyroidism	Diabetes mellitus, hypothyroidism
Infectious	COVID‐19, candidiasis, Epstein–Barr virus, citomegalovirus, botulism, meningitis, Lyme disease, diphtheria, syphilis	COVID‐19, candidiasis, herpes simplex virus
Congenital/developmental	Craniofacial anomalies (cleft lip and/or palate, Pierre Robin sequence, Treacher Collins syndrome), neuromuscular disorders (cerebral palsy, congenital myopathies, Moebius syndrome), congenital brain malformations (Chiari malformation, hydrocephalus), congenital laryngeal abnormalities (laryngomalacia, tracheoesophageal fistula), genetic syndromes (Down syndrome, DiGeorge syndrome, Prader–Willi syndrome)	Esophageal atresia, tracheoesophageal fistula (TEF), congenital esophageal stenosis, vascular rings and slings, esophageal webs and rings (e.g., congenital Schatzki ring)
Structural/mechanical	Zenker's diverticulum, cervical osteophytes, cricopharyngeal bar	Esophageal strictures, esophageal webs, Schatzki rings, hiatal hernia, esophageal diverticula, extrinsic compression
Oncological	Squamous cell carcinoma, lymphomas, salivary gland cancer, laryngeal cancer, hypopharyngeal cancer, nasopharyngeal cancer, metastatic cancers, treatment‐related causes	Squamous cell carcinoma, adenocarcinoma, lung cancer, mediastinal lymphoma, thyroid cancer, metastatic cancers, treatment‐related causes

Statement 2.2 UEG/ESNM recognize that motility disorders identified on HRM are generally associated with symptoms, primarily dysphagia.

Level of evidence: high, grade of recommendation: strong

Statement endorsed, overall agreement: 82% (A+ 59%, A 23%, A− 5%, D− 3%, D 5%, D+ 5%)

**Summary of evidence**

In the absence of organic pathology such as cancer, eosinophilic esophagitis and anatomic abnormalities (such as a large hiatus hernia), esophageal motility disorders are the primary cause of esophageal dysphagia [67]. Esophageal manometry, developed to diagnose these disorders, has been successfully utilized [68]. HRM marks a significant advancement in identifying esophageal motility disorders [69]. According to the latest iteration of the Chicago classification (v4.0), esophageal motility disorders are categorized into disorders of esophagogastric junction outflow and disorders of peristalsis [6]. Each of these disorders can explain esophageal dysphagia [70, 71]. A multicenter study found HRM to be superior to conventional manometry in diagnosing dysphagia caused by motility disorders, particularly achalasia [72]. Consequently, esophageal motility disorders identified by HRM can be considered a cause of esophageal dysphagia. HRM has also helped define new motility disorders causing dysphagia, such as EGJ‐OO and hypercontractile esophagus. Artificial intelligence holds promise for enhancing HRM diagnosis of esophageal motility disorders as a cause of dysphagia [73]. HRM investigations should be preceded by a detailed history, clinical examination, and upper digestive endoscopy, and, if necessary, complemented by pH‐impedance or Functional Lumen Imaging Probe (FLIP) evaluation.

Statement 2.3 UEG/ESNM recognize that spastic (premature) esophageal contraction is a mechanism underlying the symptoms of esophageal dysphagia.

Level of evidence: low, grade of recommendation: weak

Statement endorsed, overall agreement: 90% (A+ 62%, A 28%, A− 8%, D− 0%, D 2%, D+ 0%)

**Summary of evidence**

Distal esophageal spasm (DES) occurs due to defective propagation of peristaltic waves through the esophageal wall, while the LES relaxation is normal [74]. This condition is characterized by pathologically rapid wave progression or even simultaneous, non‐peristaltic contraction in the distal, smooth muscle esophagus, an abnormality that impairs propagation of the food bolus through the esophagus [74, 75].

The development of HRM and the Chicago classification of esophageal motility disorders introduced the concept of premature contraction for the first time in 2012, defined by a distal latency of < 4.5 s [76]. The Chicago definition of DES requires that at least 20% of swallows induce premature contractions [6, 76]. This definition shifted the focus from peristaltic velocity to latency as the crucial defining factor for DES, based on a retrospective review of more than 1000 manometric studies among which 91 patients had simultaneous contractions: the rapid contraction group was very heterogeneous while the premature group more closely fitted the clinical spectrum of DES [74].

Recent studies have demonstrated that including a standardized solid test meal in HRM significantly increases the sensitivity of diagnosing major motility disorders, particularly in patients with dysphagia, compared to single water swallows. Indeed, 67% of patients with dysphagia were diagnosed with a major motility disorder using solid swallow, compared to only 35% with single water swallows, suggesting that solid bolus ingestion may better replicate the esophageal challenges encountered during normal eating [77]. It is important to note that the solid meal used in studies that redefined the IRP cut‐off included buttered bread, soft biscuits, dumplings, or cake, as suggested by Chicago Classification (v4.0). Solid bolus swallows result in higher IRP values due to increased viscous resistance, better replicating real‐life esophageal challenges. HRM measurements during solid swallows follow a protocol similar to wet swallows, with 1–2 cm cubes of solid food taken, chewed, and swallowed. At least 5 solid swallows (preferably 10) should be performed, allowing 30 s between swallows to prevent interference. A pathological EGJ function is defined as IRP > 25 mmHg, with effective esophageal contractions requiring peristalsis (DL > 4.5 s), minimal front break (< 3 cm), and DCI > 1000 mmHg·cm·s [78].

The pathophysiology of premature contractions involves a defect of deglutitive inhibition. Swallowing induces an inhibitory wave that is followed by a contractile wave involving both circular and longitudinal muscles in a peristaltic fashion along the esophageal body. Deglutitive inhibition in the skeletal muscle of the esophagus is controlled in the brain stem whilst in the smooth muscle it is determined by regional gradients of inhibitory (nitrergic) and excitatory (acetylcholinergic) myenteric nerves, and the intrinsic properties of the smooth muscle [79]. Distal latency is a manometric marker of inhibition that defines premature contraction and thus DES: distal latency measures the end of inhibition and post‐inhibition after contraction [79].

The role of premature contractions as a cause of dysphagia is supported by observational studies [80]. However, DES may be observed incidentally, and the latest iteration of the Chicago classification considers that a conclusive diagnosis of DES implies the presence of symptoms such as dysphagia or chest pain [6]. Moreover, data obtained before the use of HRM have shown that 50% of patients with DES have normal bolus transit, thus supporting that these patients represent a heterogeneous group in which other factors, such as esophageal hypersensitivity may play a significant role [81, 82].

Statement 2.4 UEG/ESNM recognize that hypercontractile motility is a mechanism underlying symptoms of esophageal dysphagia.

Level of evidence: moderate, grade of recommendation: weak

Statement endorsed, overall agreement: 92% (A+ 67%, A 25%, A− 8%, D− 0%, D 0%, D+ 0%)

**Summary of evidence**

Hypercontractile esophagus (HE) is an esophageal motility disorder that in the past was referred to as “Nutcracker” or “Jackhammer” esophagus [6]. HE is characterized by excessive peristaltic vigor, which can be associated with high pressure of the LES in the absence of disorders of the esophago‐gastric junction (EGJ) outflow [6]. Chicago classification v.4 diagnoses HE when at least 2 out of 10 liquid swallow‐induced peristaltic contractions have a Distal Contractile Integral (DCI) > 8000 mmHg·s·cm (contractile pressures typically > 280 mmHg) [6]. Three different sub‐groups of HE are observed: single‐peaked hypercontractile swallows, jackhammer esophagus and hypercontractile swallows with a vigorous LES after‐contraction [6].

The pathophysiology of HE seems to be related to an excess of cholinergic stimulation that may be associated also with a temporal asynchrony of both internal and external esophageal muscle layers, as demonstrated by HRM provocative tests [83].

A conclusive diagnosis requires both a conclusive manometric diagnosis and the presence of clinically relevant symptoms [6]. In medical literature, publications have reported several symptoms associated with HE in cohorts of patients with unclear mechanisms of generation with dysphagia reported by 32%–100% [83]. Roman et al., before the last update of Chicago classification, reported that the prevalence of dysphagia was 72% of patients when at least one contraction had a DCI > 8000 mmHg·s·cm [84]. Herregods et al. confirmed this finding by observing dysphagia in 67.6% of patients with at least two contractions with DCI > 8000 mmHg·s·cm in a European cohort [85]. A Canadian meta‐analysis reached the same conclusion, underlying that dysphagia is the most commonly reported symptom in jackhammer esophagus (64.1%) [86]. Several studies have tried to link higher vigor contraction to symptom frequency or severity and have confirmed the above rates [87]. The repetitive, prolonged contractions in jackhammer esophagus are typically associated with higher DCI values and worse symptom severity [87].

We can conclude that dysphagia is the main associated symptom in patients with HE.

Statement 2.5 UEG/ESNM recognize that IEM is a mechanism underlying symptoms of esophageal dysphagia.

Level of evidence: low, grade of recommendation: weak

Statement endorsed, overall agreement: 82% (A+ 62%, A 20%, A− 8%, D− 5%, D 3%, D+ 2%)

**Summary of evidence**

According to the latest iteration of the Chicago Classification, ineffective esophageal motility (IEM) is defined as more than 70% ineffective swallows (esophageal contraction with a distal contractile integral < 450 mmHg·cm·s) or at least 50% failed peristalsis in a context of normal EGJ relaxation [6]. The definition of IEM has evolved over the years to improve the clinical relevance of this disorder [88]. The presence of IEM is common in patients with GERD and is known to prolong volume and acid clearance from the esophagus [89], but supporting data to link IEM and dysphagia are less robust.

One hypothesis to explain dysphagia in patients with IEM might be a delayed esophageal clearance induced by IEM. Ineffective swallows and mild bolus stasis were found in asymptomatic controls in a study combining videofluoroscopy and conventional manometry [90]. Recent evidence has shown that incorporating a standardized solid test meal into HRM protocols significantly enhances diagnostic sensitivity for esophageal motility disorders, particularly by improving the detection of IEM in patients with dysphagia compared to using single water swallows alone [77].

Besides the vigor of the contraction, the presence of a large break in esophageal contraction might induce delayed esophageal clearance. Indeed, breaks in peristaltic integrity predicted abnormal bolus clearance better than distal contractile integral in asymptomatic controls [91]. Furthermore, they were considered as a potential cause of unexplained dysphagia [92]. Using high‐resolution impedance manometry, a large break in esophageal contractions was more likely associated with abnormal bolus transit and more likely encountered in patients with dysphagia than in controls [93]. A recent study including 101 patients with IEM and 42 healthy controls revealed that novel impedance parameters (impedance ratio) might be useful in the diagnosis of IEM to predict symptom occurrence [94].

Interestingly, if IEM was associated with impaired bolus transit, the severity of dysphagia was associated with hypervigilance and visceral hypersensitivity in a study comparing 33 patients with IEM and 44 patients with normal esophageal motility [95]. Finally, in a series of 130 patients referred for HRM, patients with IEM (as defined by the Chicago Classification v4.0) had a similar dysphagia score as patients with normal motility, while GERD‐Q score was higher in these patients [96].

While IEM might be associated with impaired bolus transit, its role in dysphagia is not consistent. Overall, whether IEM could be associated with dysphagia is still debated and this uncertainty is reflected in the voting of the experts.

Statement 2.6 UEG/ESNM recognize that esophageal hypersensitivity is a mechanism underlying symptoms of esophageal dysphagia.

Level of evidence: very low, grade of recommendation: weak

Statement endorsed, overall agreement: 80% (A+ 45%, A 35%, A− 13%, D− 3%, D 2%, D+ 2%)

**Summary of evidence**

It is very difficult to demonstrate the role of esophageal hypersensitivity in generating dysphagia, with most evidence being indirectly deduced: the occurrence of symptoms in the absence of overt bolus stasis [80, 97, 98]. Indeed, the relationship between esophageal bolus stasis and the sensation of dysphagia is not perfect, introducing the concept of esophageal hypersensitivity as a factor contributing to this sensation [80, 99].

Esophageal hypersensitivity as a mechanism of dysphagia can be suspected in different conditions where patients experience dysphagia despite normal bolus transit [100, 101].

Dysphagia may occur in patients without any significant mucosal or structural abnormality and in the absence of significant esophageal motor disorder. This is the definition of functional dysphagia according to the Rome IV criteria [102]. The diagnosis of functional dysphagia will not be detailed here, but, in this condition, even advanced technologies such as FLIP or high‐resolution impedance manometry do not consistently demonstrate significant abnormalities.

During a rapid drink challenge with 200 mL of water, approximately 25% of patients report dysphagia, while no evidence of obstruction or hypercontractility is simultaneously observed. Most of these patients have normal motility or IEM on HRM [103].

Esophageal hypersensitivity is also suspected in patients with abnormal esophageal motility. Indeed, 50% of patients with DES and 88% of patients with HE have normal bolus transit, thus supporting that these patients represent a heterogeneous group in which hypersensitivity may play a significant role [82]. Of note, these results were obtained before the use of HRM.

Solid meals can help uncover underlying causes of esophageal dysphagia, such as esophageal motor disorders. Indeed, using solid swallows increases the sensitivity for detecting an underlying diagnosis compared to using only liquid boluses [77].

Regarding the potential underlying mechanisms, a superficial location of nociceptive sensory nerves in the proximal esophagus has recently been reported to be associated with more severe dysphagia in patients with motility disorders [81]. This suggest that the proximal esophagus, and perhaps the transition zone between skeletal and smooth muscle contraction segments, may be important in perceptive dysphagia [81]. However, this has not yet been reported in patients without significant motility disorders.

Statement 2.7 UEG/ESNM recognize that decreased EGJ distensibility is a mechanism underlying symptoms of esophageal dysphagia.

Level of evidence: high, grade of recommendation: strong

Statement endorsed, overall agreement: 87% (A+ 62%, A 25%, A− 10%, D− 0%, D 3%, D+ 0%)

**Summary of evidence**

The introduction of the Chicago Classification version 4.0 has improved our ability to establish a conclusive and clinically relevant diagnosis of esophageal motor disorders esophageal motor disorders [6].

In particular, the version 4.0 has better characterized esophagogastric junction outflow obstruction (EGJOO) [6]. Manometric diagnosis of EGJOO requires a pathological integrated relaxation pressure (IRP) both in supine and upright position, intact peristaltic activity and an elevated intrabolus pressure in at least 20% of supine swallows. EGJOO can only be diagnosed in patients with symptoms of dysphagia and/or chest pain, as its identification in asymptomatic individuals does not constitute a true diagnosis and may lead to unnecessary interventions [6]. If the manometric findings are not conclusive, then an abnormal timed barium esophagram or impedance planimetry (Endo‐FLIP) testing can provide additional evidence to support the diagnosis, and may also elucidate the underlying etiology and rule out other causes [6, 104]. Jain et al. examined the relationship between the distensibility index (DI) and IRP and assessed correlations with dysphagia symptoms in patients with achalasia and EGJOO. DI does not correlate with HRM metrics during water swallows (solids not studied) and has the strongest correlation with the severity of dysphagia [105].

Furthermore, in clinical practice, we often find abnormal nonspecific HRM findings or distinctive repetitive patterns that we are not able to decipher. They are not completely understood or defined; they don't fall within the diagnostic criteria of the Chicago Classification version 4.0, but they could clarify some symptoms [106]. We must consider that there is a poor correlation between the severity of symptoms and manometric patterns and that the physiopathology of dysphagia is associated not only with the manometric pattern but also with the altered emptying of the esophagus [107, 108]. Moreover, the CCv 4.0 recommends using solid bolus swallow to clarify the diagnosis since it has been shown that this increases the diagnostic sensitivity and accuracy [77].

A new functional technique, the FLIP, can optimize HRM results, especially when there is an inconclusive diagnosis of achalasia or EGJOO [106]. In the majority of cases of EGJOO, timed barium swallow is normal, but solid swallows have high sensitivity and specificity for uncovering the pathology [109].

FLIP is able to evaluate esophageal and esophagogastric junction distensibility, providing answers in case of symptomatic patients with normal manometry or IEM [7, 110]. An EGJ DI of ≤ 2.0 mm^2^/mmHg^−1^ indicates poor distensibility [111].

In a recent study, Choi et al. retrospectively analyzed 684 patients who underwent HRM. Four patients with dysphagia, esophageal wall thickening, and both a normal HRM result and an IEM diagnosis underwent FLIP topography. They had poor EGJ distensibility and underwent peroral endoscopic myotomy (POEM) with a good therapeutic response. FLIP alone or in combination with HRM can help identify patients who may respond to POEM. Poor EGJ distensibility may appear as an obstruction to bolus flow [106].

We can deduce that decreased EGJ distensibility is a mechanism underlying symptoms of esophageal dysphagia.

Statement 2.8 UEG/ESNM recognize that impaired LES relaxation is a mechanism underlying the symptoms of esophageal dysphagia, apart from achalasia.

Level of evidence: moderate, grade of recommendation: strong

Statement endorsed, overall agreement: 95% (A+ 67%, A 28%, A− 3%, D− 2%, D 0%, D+ 0%)

**Summary of evidence**

Swallow‐induced impaired relaxation of the LES disrupts normal transit across the EGJ [112]. In clinical practice, EGJ pressure and relaxation are assessed using HRM, though it cannot determine whether the EGJ pressure depends on the LES or on other contributors such as the crural diaphragm. Additionally, mechanical causes of EGJOO (such as stenotic lesions or external compression) may increase intrabolus pressure as seen also with impaired LES relaxation. Consequently, the Chicago Classification (CC) selected integrated relaxation pressure (IRP) as the metric to define normal versus abnormal EGJ relaxation with swallowing [6]. For this reason, impaired relaxation of the LES is manifested on HRM as median IRP above the upper limit of normal, which is 15 mmHg (supine) and 12 mmHg (sitting) [6].

Impaired LES relaxation and/or opening is observed in two major disorders of EGJ outflow: achalasia and EGJOO. In both disorders median IRP is higher than the upper limit of normal, but only in achalasia is it accompanied by complete absence of peristalsis [6]. Dysphagia is the main clinical presentation in both achalasia and EGJOO.

In achalasia, dysphagia is related to both esophageal aperistalsis and impaired LES relaxation. In achalasia, it is thought that an inflammatory process damages inhibitory neurons (nitric oxide producers) in the distal esophagus [113]. The loss of inhibitory neurons at the LES increases the basal sphincter pressure and impairs LES relaxation [114]. In the distal (smooth muscle) part of the esophagus, the loss of inhibitory neurons results in aperistalsis [114]. Although both mechanisms cause dysphagia, most of the symptoms and signs in primary achalasia are related to bolus retention in the esophagus caused by the impaired LES relaxation, which is the cause of impaired EGJ outflow in this condition [115].

EGJOO is a heterogeneous condition in which dysphagia is related to impaired EGJ outflow. Underlying mechanisms include impaired LES relaxation, which may represent an incomplete expression or evolving case of achalasia, and other causes that aren't related to the LES such as stenotic strictures, sliding hiatal hernia, opioid effect and other benign and malignant infiltrative disorders [116]. In contrast with achalasia, dysphagia due to EGJOO is not usually associated with aperistalsis, as some evidence of peristalsis is almost always present, precluding a diagnosis of achalasia. For that reason, if the underlying cause is not clear, then EGJOO‐related dysphagia should be confirmed with complementary testing such as FLIP or timed barium swallow [117]. It is important to note that EGJOO can only be diagnosed in patients with symptoms of dysphagia and/or chest pain [6].

Studies have shown that a solid bolus can reveal motor dysfunctions undetectable with a liquid bolus in approximately 20%–30% of patients. For instance, in an analysis of dysphagic patients, a solid bolus demonstrated a significant increase in IRP compared to those obtained with liquid boluses. To note, a with solid swallows the upper normal limit of IRP with solid swallows is 25 mmHg and a normal peristalsis requires a DCI > 1000 mmHg·s·cm in the absence of a significant break (> 5 cm) in the contraction front [2].

Finally, elevated basal LES pressure and impaired swallow‐induced LES relaxation were also observed in non‐achalasia elderly patients with dysphagia [56, 118]. However, the contribution of HRM findings and of healthy aging to dysphagia requires further investigation. Another controlled study that evaluated dysphagia in opioid users found that high intake was associated with impaired swallow‐induced LES relaxation and abnormal peristaltic sequence [119]. However, in that study, a significant proportion of dysphagic patients were ultimately diagnosed with achalasia type 3 and EGJOO.

Statement 2.9 UEG/ESNM recognize that esophageal dysmotility may predispose to disorders like epiphrenic diverticulum, candida esophagitis and strictures.

Level of evidence: very low, GRADE of recommendation: weak

Statement endorsed, overall agreement: 87% (A+ 62%, A 26%, A− 10%, D− 3%, D 0%, D+ 0%)

**Summary of evidence**

Various esophageal motility disorders may predispose individuals to conditions such as epiphrenic diverticulum, candida esophagitis, and strictures. Indeed, motility disorders such as achalasia, diffuse esophageal spasm, and IEM often lead to impaired clearance of food and secretions, creating an environment conducive to the development of these complications. However, there are no prospective, randomized controlled studies specifically addressing these outcomes in the context of chronic motility disorders, leaving the current level of evidence as very low.

Epiphrenic diverticula are rare protrusions through the esophageal muscular layer forming saccular cavities. The true prevalence of epiphrenic diverticula is unknown, but radiological studies have shown it to be around 0.015% in the United States, up to 0.77% in Japan, and 2.0% in Europe [120]. Esophageal dysmotility, such as achalasia and diffuse esophageal spasm, appear to be a risk factor for epiphrenic diverticula. The natural history of epiphrenic diverticula is unclear, and optimal management is debated. Symptomatic patients require therapy, but it can be challenging to determine if symptoms stem from the anatomical abnormality or the underlying esophageal dysmotility [121].

Candida esophagitis is common in immunosuppressed individuals even in the absence of motility disorders. Any impairment of esophageal clearance may lead to food and secretion stasis, promoting Candida albicans overgrowth and infection [122]. Management typically involves oral antifungal therapy, though severe cases may require intravenous treatment. Recurrence is higher if underlying motility issues are untreated.

Strictures are rare in esophageal dysmotility but can occur in overlapping conditions like eosinophilic esophagitis and GERD. GERD can cause scarring, narrowing, and impaired esophageal propulsion and clearance. Esophageal dilation is often required for narrowing or strictures, following general treatment principles and endoscopic dilation.

Other potential complications over time include aspiration pneumonia, chronic cough, and recurrent infections [114]. In cases of severe dysmotility, food and liquids may regurgitate into the airways, leading to chronic aspiration and increasing the risk of aspiration pneumonia. This is particularly common in advanced achalasia and other disorders with impaired esophageal clearance [114].

Nutritional management, particularly when the esophageal dysmotility cannot be treated endoscopically or surgically, lacks consensus and high‐quality studies, with most research focused on OD. General recommendations include small, frequent, high‐energy, high‐protein meals of appropriate consistency, avoiding large meals or snacks before bedtime, and sitting upright for 30–60 min after eating. Thickened liquids can reduce choking, aspiration, and reflux complications [123].

Statement 2.10 UEG/ESNM recognize that the main phenotypes of patients with OD are older adults, patients with neurological and neurodegenerative diseases, patients with head and neck cancer (pre‐ or post‐treatment), and patients with anatomical/structural alterations. A less prevalent group includes patients with infectious or metabolic diseases, side effects of drugs, or iatrogenic conditions.

Level of evidence: high, grade of recommendation: good practice statement

Statement endorsed, overall agreement: 93% (A+ 76%, A 17%, A− 7%, D− 0%, D 0%, D+ 0%)

**Summary of evidence**

The main phenotypes of patients with OD can be classified into four primary groups: aging, neurogenic diseases, head and neck cancer, and anatomical or structural alterations. Less prevalent causes include metabolic, iatrogenic, and infectious diseases, recently including COVID‐19 [3, 124].

OD is recognized as a major geriatric syndrome due to its high prevalence among older adults and its association with other geriatric syndromes, such as functional and cognitive dependency [125]. Age‐related changes in the swallowing system include osteophytes in the cervical spine, sarcopenia, reduced muscle mass, tissue elasticity, saliva production, poor dental status, and weakened oral and pharyngeal muscles [126]. These changes lead to difficulties in all phases of swallowing. For instance, impaired lip closure can cause bolus spillage, and tongue sarcopenia can result in weak propulsion and premature spillage, leading to aspiration. Elderly individuals also exhibit impaired oropharyngeal sensory function and reduced cough reflex, increasing the risk of silent aspiration [127].

Oropharyngeal dysphagia in neurogenic diseases includes both non‐progressive and progressive neurological disorders. Stroke patients often have impairments in all swallowing phases due to cortical lesions, leading to aspiration, pharyngeal residue, and impaired swallowing response. Subcortical and brainstem lesions can cause pharyngeal phase dysfunction and a prolonged risk of aspiration [128]. Traumatic brain injury (TBI) can result in dysphagia from neurological and cognitive deficits, affecting oral and pharyngeal phases and increasing aspiration risk due to glottic anatomical changes [129]. Cerebral palsy (CP) involves movement and motor function disorders causing dysphagia, with difficulties in oral and pharyngeal phases, including inadequate lip seal, reduced tongue movement, and aspiration [130].

Dysphagia in Parkinson's disease (PD) results from degeneration of dopaminergic and non‐dopaminergic pathways, impacting all swallowing phases. PD patients can also have impaired esophageal motility. Additionally, there is increased aspiration risk due to muscle rigidity and coordination issues. In dementia and Alzheimer's disease (AD), progressive cognitive decline and cortical network changes cause dysphagia, with prolonged bolus preparation, delayed swallow initiation, and pharyngeal residue leading to aspiration [131].

In head and neck cancer patients, dysphagia depends on tumor size, location, and treatment [132]. Radiotherapy and chemoradiotherapy can cause long‐term neuromuscular injury, with early radiation‐induced dysphagia characterized by inflammation and mucositis, and late dysphagia by fibrosis and muscle atrophy, impairing swallowing kinematics and increasing aspiration risk [132]. Structural alterations like cervical osteophytes, cricopharyngeal bar, and Zenker's diverticulum impair swallowing by causing airway protection issues and pharyngeal residue, with Zenker's diverticulum linked to restricted UES opening and significant dysphagia symptoms [133]. Other causes of OD include metabolic, iatrogenic, and infectious diseases, with COVID‐19 recently identified as a contributor due to associated comorbidities and neurological symptoms [3, 124].

Statement 2.11 UEG/ESNM recognize that patients with OD have impaired biomechanics of the oropharyngeal swallow response, including delayed total deglutition time, delayed time to laryngeal vestibule closure, delayed UES opening, and reduced bolus kinematics (propulsion force and bolus velocity). Neurophysiologically, they exhibit impairments in both afferent (sensory) and efferent (motor) pathways. Additionally, specific structural changes in the oral cavity, pharynx, larynx, and UES can lead to the development of structural OD.

Level of evidence: moderate, recommendation: good practice statement

Statement endorsed, overall agreement: 86% (A+ 67%, A 19%, A− 0%, D− 3%, D 5%, D+ 6%)

**Summary of evidence**

Patients with OD (OD) have impaired biomechanics of the oropharyngeal swallow response, including delayed total deglutition time, delayed time to laryngeal vestibule closure (LVC), delayed UES opening (UESO), and reduced bolus kinematics (propulsion force and bolus velocity) [3]. Neurophysiologically, they may exhibit impairments in both afferent (sensory) and efferent (motor) pathways. Additionally, specific structural changes in the oral cavity, pharynx, larynx, and UES can lead to the development of structural OD [3].

OD can result from biomechanical alterations in oropharyngeal physiology, affecting swallow safety and efficacy. Impaired tongue movement and mastication during the oral phase can cause pre‐deglutitive aspiration, where food falls into the pharynx and open airway before swallowing. Delays in triggering the oropharyngeal swallow response and LVC can lead to aspiration during swallowing. Weak tongue propulsion, reduced laryngeal elevation, and weak pharyngeal contraction cause residue in the pharynx, potentially leading to post‐deglutitive aspiration [3].

The central nervous system mediates swallowing through dynamic interactions between sensory and motor neural pathways. Functional imaging studies show cortical involvement in swallowing, with activity in several cortical regions, including the primary motor cortex, sensory cortex, insula, and supplementary motor area [134]. Sensory inputs from the oral and pharyngeal regions are essential for initiating and regulating swallowing motor sequences. These sensory signals are processed in the brainstem and cortex, generating motor signals that execute swallowing [135].

In older and post‐stroke patients with OD, studies show impairments in sensory and motor pathways. In older patients with OD have higher motor thresholds and delayed motor responses, indicating reduced excitability of the pharyngeal motor cortex [136]. Dysphagia in neurological disorders, such as Parkinson's disease (PD) and amyotrophic lateral sclerosis (ALS), is associated with distinct cortical activation patterns, suggesting unique adaptive mechanisms [137].

Structural alterations in the oral cavity, pharynx, larynx, and UES can cause OD. Head and neck cancer (HNC) and its treatments (surgery and radiotherapy) can lead to dysphagia due to tumor obstruction, tissue damage, and fibrosis [132]. Cervical osteophytes, typically seen in the elderly, can impair swallowing by causing airway obstruction and reduced laryngeal elevation [133]. Zenker's diverticulum is associated with reduced UES opening, leading to significant dysphagia symptoms [138].

Statement 2.12 UEG/ESNM recognize that OD is associated with serious nutritional and respiratory complications, including malnutrition, dehydration, and respiratory infections such as aspiration pneumonia. These complications are linked to poor health outcomes, including hospital readmissions and increased morbidity and mortality.

Level of evidence: moderate; recommendation: good practice statement

Statement endorsed, overall agreement: 90% (A+ 76%, A 14%, A− 7%, D− 0%, D 0%, D+ 3%)

**Summary of evidence**

Oropharyngeal dysphagia (OD) leads to severe complications, including choking, tracheobronchial aspiration, and aspiration pneumonia (AP), as well as malnutrition (MN) and dehydration (DH), potentially resulting in morbidity and mortality [3]. OD is a significant risk factor for lower respiratory tract infections in older, independently living patients [139].

Among stroke patients, up to 20% suffer from early AP, a major cause of mortality within the first year after discharge. AP is also the leading cause of death in Parkinson's disease patients [140]. The prevalence of AP is underestimated, estimated at 5%–15% among patients hospitalized with community‐ and hospital‐acquired pneumonia [141].

Poor oral health, malnutrition, and aspiration are major risk factors for AP in older patients and those with neurological diseases [141, 142]. A possible mechanism involves reduced neurotransmitter release, impairing the swallow response and cough reflex [141, 142]. Microaspiration during sleep may be considered normal, but large‐volume aspiration (macroaspiration) of colonized secretions is a major cause of AP [142].

A study involving 2359 elderly patients found that OD was diagnosed in 47.5%, with 7.9% experiencing pneumonia readmissions, indicating a higher incidence in dysphagic patients [125]. AP leads to muscle atrophy and pneumonia‐associated sarcopenia, contributing to recurrent pneumonia, feeding difficulties, and malnutrition. Frailty‐associated pneumonia primarily affects adults over 80, leading to significant functional decline and mortality [143]. The increasing elderly population and life expectancy contribute to rising pneumonia incidence, emphasizing the need for effective diagnosis, treatment, and prevention [3, 142].

Impaired swallowing efficacy, associated with reduced tongue propulsion and volume due to sarcopenia, leads to malnutrition and dehydration [139]. Malnutrition prevalence varies by patient phenotype; in independent older adults with OD, the annual incidence is 18.6%, with a 26% prevalence compared to 11.4% without OD [139]. In acute geriatric units, malnutrition is present in 45.3% of OD patients, linked to longer hospital stays, higher institutionalization risk, and increased mortality [144]. There is limited high‐quality literature on malnutrition and dysphagia prevalence in long‐term care [145].

A 2009 review found a strong relationship between dysphagia and malnutrition following stroke, with malnutrition prevalence ranging from 8.2% to 49% [146]. Neurodegenerative diseases like multiple sclerosis and Parkinson's present high OD risks, significantly influencing clinical outcomes [140]. In children with cerebral palsy, OD contributes significantly to malnutrition and dehydration [130]. A 2022 review found dehydration prevalence in OD patients ranged from 19% to 100%, with low thickened fluid consumption necessitating strict monitoring [147].

Statement 2.13 UEG/ESNM recognize dysphagia and anxiety/depression can be associated conditions. While this link is well addressed in the case of OD, data on esophageal dysphagia are scarce.

LE: very low; recommendation: expert opinion

Statement endorsed, overall agreement: 87% (A+ 44%, A 44%, A− 10%, D− 3%, D 0%, D+ 0%)

**Summary of evidence**

Previous studies have assessed the putative link between dysphagia and psychological and emotional disorders, mainly depression and anxiety. Importantly, most of these studies have addressed OD, not esophageal dysphagia [52, 56, 148, 149]. Ekberg et al. investigated the psychological and social impact of dysphagia on a sample of 350 patients. Forty‐one percent of patients reported that they experienced panic or anxiety during mealtimes, with 36% avoiding eating with others because of their dysphagia [52]. This study found an independent relationship between intermittent dysphagia and anxiety, while progressive dysphagia was associated with depression. Nonetheless, no specific reason for this finding was determined by studies that have reported links between dysphagia and psychological/emotional disorders [52, 149].

Eslick and Talley [28] performed a survey‐based epidemiological study that aimed to estimate the prevalence of dysphagia in an Australian population and assessed the correlation to anxiety and depression among patients with dysphagia compared to those without dysphagia. The prevalence of clinical depression was 7% (95% CI: 3%–14%) versus 5% (95% CI: 3%–7%), anxiety 20% (95% CI: 13%–29%) versus 14% (95% CI: 11%–17%). Analyses of those with dysphagia compared with no dysphagia found that there was a significant difference for anxiety (OR = 1.08, 95% CI: 1.03–1.13) and depression (OR = 1.06, 95% CI: 1.01–1.13). Several studies have reported that patients with OD (from several etiologies, i.e., mental, neurological, oncological etc.), may also suffer from increased psychological conditions such as of anxiety and depression [150, 151, 152]. Nonetheless, no accurate data exist regarding the link between esophageal dysphagia and anxiety/depression.

Finally, it is important to highlight that this association may fall within the context of functional dysphagia, which is defined according to the Rome IV criteria as a difficulty in swallowing in the absence of structural, motor, or obstructive abnormalities that could explain the symptoms. Functional dysphagia is diagnosed when no identifiable organic causes are found [153].

### 3.3 Section 3: Diagnosis

Statement 3.1 UEG/ESNM recommends careful focused history taking, although it is insufficient to differentiate between esophageal and OD.

Quality of evidence: very low; recommendation: expert opinion

Statement endorsed, overall agreement: 85% (A+ 65%, A 20%, A− 10%, D− 5%, D 0%, D+ 0%)

**Summary of evidence**

A careful history taking is an important first step when assessing dysphagia. It could aid in identifying the anatomical origin (esophageal vs. oropharyngeal) of the symptoms as well as its pathophysiological background (obstructive vs. non‐obstructive) [154]. For instance, globus sensation and xerostomia are common conditions that could mimic OD and should be carefully evaluated during history taking [53]. Moreover, distinguishing between oropharyngeal and esophageal dysphagia is crucial to identify the etiology and plan a correct diagnostic work up [155]. Notably, direct questioning regarding the location where the patient is experiencing dysphagia has limited accuracy [156]. This discomfort was felt anteriorly at the suprasternal notch and not posteriorly at the back of the throat. This is in line with the fact that OD is not uncommon in achalasia [157, 158]. Besides questioning regarding the site of the swallowing difficulty sensation, four questions have been highlighted for their practical clinical utility in differentiating oropharyngeal from esophageal dysphagia with 80% accuracy if any of the questions are answered positively ((1) Is there a delay in initiating the swallow? (2) Is there deglutitive postnasal regurgitation? (3) Is there deglutitive coughing? (4) Is repetitive swallowing needed to achieve satisfactory clearance?) [159]. Lastly, careful history taking could aid in recognizing the primary pathology likely to be responsible for esophageal dysphagia (obstructive vs. non‐obstructive/esophageal motility disorder) [160]. In particular, it is imperative to differentiate whether the dysphagia is related to solids and liquids (suggestive of non‐obstructive pathologies), or solids alone (suggestive of obstructive pathologies), and whether it is progressive and worsening over time (obstructive) or intermittent (non‐obstructive) [53, 154].

Statement 3.2 UEG/ESNM recommend upper gastrointestinal endoscopy (EGD) to evaluate esophageal dysphagia in all adult patients. EGD also plays a pivotal role in cases of acute onset of inability to swallow, which suggests a food impaction in the esophagus.

Level of evidence: low, grade of recommendation: strong

Statement endorsed, overall agreement: 92% (A+ 79%, A 13%, A− 5%, D− 0%, D 3%, D+ 0%)

**Summary of evidence**

Since the introduction of flexible fiberoptic endoscope, there has been an explosion of technical achievements in EGD that enable direct assessment of the esophagus‐stomach‐duodenum, allow biopsies and, when required, therapeutic interventions [161]. Most guidelines recommend EGD as the first test for the evaluation of esophageal dysphagia to define the exact cause and to initiate appropriate therapy [162]. Dysphagia only in relation to the ingestion of solids suggests a mechanical obstruction. Dysphagia to both solids and liquids is probably due to an underlying esophageal motility disorder. Esophageal dysphagia due to malignancy progresses more rapidly. Additionally, esophageal dysphagia has a high predictive value for cancer in older patients [163]. However, a definitive diagnosis cannot be established in most instances by history alone [164], reinforcing the clinical dogma that patients need urgent endoscopy. Dysphagia in a primary care setting is associated with an overall positive predictive value up to 5.5% for esophageal cancer and the National Institute for Health and Care Excellence (NICE) in the UK recommends that direct, open access EGD should be offered to all patients with dysphagia within 2 weeks to rule out an organic process [165]. Dysphagia has a high predictive value for finding significant pathology [163]. It is equally important to make a precise and early diagnosis of a major non‐malignant condition, so that treatment can be initiated rapidly instead of waiting for diagnoses to emerge [166]. A sudden acute onset of inability to swallow solids and/or liquids, including secretions, suggests food impaction in the esophagus. It represents a major challenge and EGD is the most common therapeutic approach [167].

Statement 3.3 UEG/ESNM recognize that the yield of EGD for diagnosing structural abnormalities underlying esophageal dysphagia varies among studies. However, structural abnormalities are well visualized on EGD. Erosive reflux disease that can progress to peptic strictures and EoE, which can be associated with fibrotic narrowing of the esophagus, are common. Idiopathic fibrosis (Schatzki ring), complications of hiatus hernia, as well as malignancies, are less frequent.

Level of evidence: low, grade of recommendation: strong

Statement endorsed, overall agreement: 93% (A+ 63%, A 30%, A− 3%, D− 0%, D 2%, D+ 2%)

**Summary of evidence**

The diagnostic role of EGD in the initial evaluation of dysphagia has evolved over time. In the past, it was considered an invasive and expensive tool, so its use was restricted. However, when endoscopic therapy complements the diagnostic value of endoscopy, cost savings could be achieved compared to a diagnostic approach starting with barium esophagography followed by therapeutic endoscopy [168] and, in addition, the overall incidence of complications of diagnostic endoscopy is low (0.1%) [169]. EGD allows for the direct visualization of structural abnormalities such as strictures, rings, esophagitis, mucosal features of eosinophilic esophagitis (EoE), Barrett's and cancer that commonly share the process of narrowing the lumen of the esophagus with subsequent delay in passage of solid foods. Infectious esophagitis, such as that caused by Candida, herpes simplex virus (HSV), or cytomegalovirus (CMV), can also be identified with EGD, often presenting with characteristic mucosal lesions or ulcers that may require biopsy for definitive diagnosis [65]. Krishnamurthy et al. analyzed retrospectively the frequency of various endoscopic findings in 30,377 patients who underwent EGD for the evaluation of dysphagia and found abnormal findings in 66% of them, with esophageal strictures being the most frequent, followed by esophagitis/ulcers and Schatzki's ring. Other etiologies that accounted for < 10% were retained food and suspected malignancy [170]. It should also be recognized that GERD might be associated with esophageal dysphagia [171]. A recent study confirmed the presence of abnormalities at EGD in 64% of the patients with dysphagia with a higher percentage of esophagitis (36%) and, a decrease in peptic strictures (1.43%) compared to previous studies [172]. Hiatal hernia was a frequent finding (31%), however, it is unlikely that small and uncomplicated hernias could cause dysphagia by a mechanical mechanism [173]. Barrett's esophagus, Schatzki's ring, malignancies and candidiasis accounted for < 10% in this study [172]. There is evidence of a change in the relative proportion of the various disease in the last few decades [174]. In Western countries benign esophageal peptic stricture has become less common. This finding is possibly due to the increase in the use of proton pump inhibitors (PPI) [175], while EoE is increasing. Currently, EoE is identified in 12%–23% of patients undergoing EGD for dysphagia and appears to be rising in incidence at a rate faster than would be expected based solely on increased recognition of the disease [2].

Statement 3.4 UEG/ESNM suggest that esophageal biopsy sampling should be obtained in all patients with dysphagia, especially if clinical characteristics (male, young age, history of atopy or allergy) are suggestive for eosinophilic esophagitis even if macroscopic endoscopy results normal.

Level of evidence: low, grade of recommendation: strong

Statement endorsed, overall agreement: 97% (A+ 76%, A 22%, A− 3%, D− 0%, D 0%, D+ 0%)

**Summary of evidence**

Esophageal biopsies in patients with dysphagia should be collected in all cases of evidence of mucosal lesions, ulcers, nodules, or precancerous lesions. New advances in endoscopic techniques and technology have improved diagnostic accuracy and increased the therapeutic potential of EGD [176, 177, 178, 179]. Despite that, routine biopsies in dysphagic patients may still be warranted to avoid missed diagnoses, especially in non‐classical presentations of eosinophilic esophagitis (EoE). According to major guidelines on the management of EoE, at least 6 esophageal biopsies should be obtained from a minimum of 2 esophageal levels (e.g., proximal/mid and distal), ideally targeting endoscopic findings of EoE to optimize the assessment of histologic features consistent with the condition [180, 181].

Dellon et al. developed in 2015 a predictive model in which younger age, male gender, white race ethnicity, history of atopy or food allergies, and presence of dysphagia of food impaction were highly associated with risk of eosinophilic esophagitis (87%–90%) [182]. A similar model was recently evaluated by Cotton et al. The authors showed that clinical features and endoscopic findings were able to identify patients with EoE with an AUC of 0.92 even without histologic data and in the absence of dysphagia [183]. The authors concluded that a predictive model could help clinicians to make decisions in patients with upper GI symptoms about whether to perform early diagnostic endoscopy or gastroenterology referral for suspicion of EoE [183, 184]. Prasad et al. showed that EoE was present in at least 10% of patients with solid food dysphagia who appeared endoscopically normal. An endoscopic appearance suggestive of EE accurately predicted the presence of EoE in 38% of patients. Younger age, history of food impaction, lack of PPI use, and endoscopic appearance suggestive of EE predicted a higher likelihood of EE on biopsy [185]. Diagnosis of EoE was found in 10% of normal endoscopies, in 14% of patients with reflux esophagitis; 5% of patients with Schatzki's ring, in 50% of patients with esophageal non‐neoplastic strictures and in 38% of patients with endoscopic findings of EoE. This study supports a practice of routine esophageal biopsy in all patients with non‐malignant solid food dysphagia regardless of endoscopic findings.

Mackenzie et al. showed in a large group of patients that EoE was diagnosed in 12% of the patients presenting with dysphagia with relative risk of 9.5 if age < 50 years. Esophageal biopsies are warranted in patients presenting with dysphagia especially in the younger population [186]. In this study, 13 of 31 (41%) EoE patients did not have findings thought to be classical for EoE and would have been missed without esophageal biopsies.

A meta‐analysis from Kim et al. concluded that esophageal biopsies should be obtained from all patients who present with symptoms of EoE, regardless of the endoscopic appearance of the esophagus [176].

Statement 3.5 UEG/ESNM recognize that Edema, Rings, Exudates, Furrows and Stenosis are the most relevant endoscopic findings of EoE.

Level of evidence: high, grade of recommendation: strong

Statement endorsed, overall agreement: 95% (A+ 71%, A 24%, A− 3%, D− 2%, D 0%, D+ 0%)

**Summary of evidence**

On a practical level, endoscopic assessment is easy and reliable, requiring little additional time during standard‐of‐care procedures. Studies in the U.S. and Europe have validated the EoE Endoscopic Reference Score (EREFS) with high inter‐ and intra‐observer agreement, showing 90% sensitivity and specificity in both pediatric and adult patients [187, 188, 189, 190, 191]. Additionally, the responsiveness of the EREFS to therapy has been confirmed in clinical trials, with placebo studies supporting the objectivity of the endoscopic assessment, free from placebo effect bias [192, 193]. Normalization of inflammatory features provides immediate feedback on the effectiveness of medical or dietary interventions, guiding decisions on esophageal dilation in dysphagia cases when disease activity is controlled.

The EREFS score, a validated classification system for endoscopic findings in EoE, stands for furrows (linear or longitudinal: 80%), edema, rings (64%), exudates (16%), and strictures (12%), showing high diagnostic accuracy [187, 191]. Longitudinal furrows appear as long, linear lines parallel to the esophagus's long axis, while esophageal exudates, whitish plaques, may be misinterpreted as candidiasis [187]. Edema is characterized by loss of vascular pattern and mucosal pallor [187]. Fibrotic changes can be focal, long, or diffuse, and milder strictures with a diameter > 10 mm can be subtle [187]. However, patients with EoE may have a normal‐appearing esophagus during endoscopic examination [194]. Esophageal biopsy remains necessary to confirm suspected EoE [195].

The EREFS classification effectively identifies most patients with EoE, though its performance in determining treatment response is limited, with an area under the curve from 0.79 to 0.88 when histologic remission is defined as a peak eosinophil count ≤ 15 eos/HPF [190, 191, 196]. A meta‐analysis by Kim et al. showed higher prevalence of individual endoscopic findings (rings, furrows, exudates) in prospective studies compared to retrospective ones. Prevalence of findings also varied by age, with rings and strictures more common in adults, and white plaques and pallor more common in children [176].

The updated international consensus diagnostic criteria for eosinophilic esophagitis (AGREE conference) confirmed that endoscopy should evaluate for EoE signs according to the EREFS Score and alternative esophageal disorders, with numerous biopsies collected [197].

The University of North Carolina, Chapel Hill, developed an AI algorithm (AI‐EoE‐EREFS) that outperformed human endoscopists of varying experience levels. The AI algorithm showed a sensitivity of 0.96, specificity of 0.94, accuracy of 0.95, and an AUC of 0.992, performing significantly better than beginners and senior fellows on the same set of images [198].

Statement 3.6 UEG/ESNM recommend the use of HRM in esophageal dysphagia as HRM is the most important technology focused on assessing esophageal motor function causing dysphagia in endoscopy‐negative dysphagia patients.

Level of evidence: high, grade of recommendation: strong

Statement endorsed, overall agreement: 95% (A+ 74%, A 21%, A− 5%, D− 0%, D 0%, D+ 0%)

**Summary of evidence**

Patients suspected of having esophageal dysphagia should be referred for an EGD, as this test helps rule out mechanical obstruction or an inflammatory process; however, endoscopic observations only occasionally indicate the presence of an esophageal motor disorder [199, 200]. The most important technology for assessing esophageal motor function in cases of endoscopy‐negative dysphagia is HRM [201]. Since its introduction over two decades ago, HRM has evolved significantly, with major advancements in classification schemes and biomarkers of esophageal function. The current classification scheme for esophageal motor dysfunction is the Chicago Classification v4.0 [6]. Compared to conventional manometry, HRM has vastly improved sensitivity for detecting achalasia, primarily due to its objective and accurate identification of impaired esophagogastric junction (EGJ) relaxation [115].

Multiple rapid swallows (MRS) assess esophageal contractile reserve by inducing inhibition of esophageal peristalsis followed by a strong peristaltic contraction, which helps identify achalasia or motility disorders when abnormalities are present. Rapid drink challenge (RDC), using a larger liquid volume, increases sensitivity in detecting EGJOO. Additionally, solid bolus testing during HRM evaluates esophageal function in conditions more similar to daily life, helping to identify motility disorders not evident with liquid boluses and better correlating with patient symptoms such as dysphagia [202].

HRM offers several advantages over conventional manometry [199]: it allows simultaneous viewing of the entire esophagus's contractility in a uniform, standardized format [200], it provides standardized, objective metrics of peristaltic and sphincter function for systematic interpretation, and [201] it enables easier recognition of topographic patterns of contractility with greater reproducibility [203].

Statement 3.7 UEG/ESNM suggest the use of pH and impedance‐pH monitoring as it might provide information on the possible presence of GERD or esophageal hypersensitivity.

Level of evidence: moderate, grade of recommendation: weak

Statement endorsed, overall agreement: 87% (A+ 56%, A 31%, A− 3%, D− 3%, D 8%, D+ 0%)

**Summary of evidence**

Patients with typical GERD symptoms often complain of dysphagia [171]. Studies have shown that 40% of patients with non‐obstructive dysphagia exhibit a pathological acid exposure time. For these patients, adequate PPI therapy significantly decreases the frequency and severity of dysphagia [204] likely because reduced acid exposure can also reduce esophageal sensitivity (see below). Additionally, a considerable proportion of GERD patients present impaired esophageal motility, which may explain the occurrence of dysphagia [205]. However, the presence of IEM does not necessarily identify patients with dysphagia, as not all GERD patients with dysphagia have evidence of IEM [77, 206].

The discrepancy between dysphagia perception and esophageal function suggests other factors, such as hypersensitivity to normal bolus passage, might contribute to dysphagia in GERD patients. It has been demonstrated that pre‐sensitization of esophageal acid‐sensitive chemoreceptors in GERD patients reduces pain threshold and increases pain perception following balloon distension [207]. Furthermore, psychogenic factors like anxiety, stress, and excessive hypervigilance—commonly reported in GERD patients—may alter the cortical response to stimuli mediated by intra‐esophageal content [208].

In this context, catheter‐based impedance‐pH monitoring or prolonged, wireless pH monitoring can provide information on concomitant GERD or the possible presence of esophageal hypersensitivity, which may be associated with dysphagia perception, especially in patients experiencing anxiety, stress, and hypervigilance [209].

Statement 3.8 In patients with esophageal dysphagia, UEG/ESNM suggest a barium esophagram as complementary investigation to improve the diagnosis of structural and functional esophageal disease when endoscopy and HRM did not fully explain symptoms.

Level of evidence: moderate, grade of recommendation: strong

Statement endorsed, overall agreement: 97% (A+ 72%, A 26%, A− 3%, D− 0%, D 0%, D+ 0%)

**Summary of evidence**

In patients with esophageal dysphagia, a barium esophagram may be useful before endoscopy if a proximal esophageal stricture or diverticulum or high‐risk lesion is suspected, or in patients at high risk of perforation due to strictures from caustic ingestion or other pathology [194]. It is also valuable when endoscopy access is challenging, to assess major structural lesions and prioritize endoscopy and specialist evaluation. If the history suggests a proximal esophageal obstruction or a very tight stricture, a fluoroscopic swallowing study with a barium esophagogram can be the most informative initial investigation [155]. The investigation has high sensitivity for diagnosing rings, benign esophageal strictures, hiatal hernia, peptic esophagitis, esophageal motor abnormalities, diverticula, and tumors. It also better delineates extrinsic compression of the esophagus, such as from vascular anomalies like an aberrant right subclavian artery (arteria lusoria) [210, 211]. When esophageal manometry is unavailable, the esophagogram can provide preoperative information for disorders like achalasia or structural alterations such as Zenker's diverticulum. It is an alternative for patients unable to tolerate manometry or when manometric studies are of poor quality or unclear [212].

For diagnosing esophageal motor disorders, specific characteristics in the esophagogram, such as a dilated upper esophagus with a bird's beak appearance, incomplete LES opening, loss of primary peristalsis, and delayed esophageal emptying, are widely accepted for achalasia [213]. Studies have shown that delayed emptying correlates with increased LES pressures, demonstrating a positive correlation between radiographic and manometric findings in achalasia patients [212, 214]. However, esophageal manometry is preferred over the esophagogram for diagnosing esophageal motor disorders [210]. Some studies indicate that the esophagogram has low sensitivity when the manometric study is altered, but it has higher sensitivity for diagnosing achalasia compared to other motility disorders when nutcracker esophagus and other non‐specific motor disorders are ruled out [215, 216, 217].

In a study by Blonski et al. on 309 patients with esophageal motor disorders using timed barium esophagogram (TBE), a barium height of 5 cm at 1 min had a sensitivity of 94% and specificity of 71%, and a height of 2 cm at 5 min had a sensitivity of 85% and specificity of 86% for differentiating untreated achalasia from EGJOO and non‐achalasia [212]. A European study of 30 patients with achalasia found that EGJ distensibility correlated strongly with barium retention height at 5 min, while LES pressure showed a poor correlation with stasis height. This confirmed TBE's efficacy as a surrogate for EndoFlip assessment of EGJ compliance [218]. Moreover, studies show that patients who resolve their achalasia pattern based on HRM improve emptying and symptom scores. The esophagogram is useful for assessing esophageal emptying post‐treatment and predicting future symptom recurrence in achalasia [219].

Combining manometry and esophagogram can be useful in cases like EGJOO or post‐fundoplication dysphagia, as the esophagogram complements functional alteration definition and guides treatment, assessing esophageal emptying [220]. The Chicago Classification version 4.0 suggests that a clinically relevant EGJOO diagnosis requires supportive investigations such as TBE [221], preferably with a barium tablet swallow, and/or FLIP [6]. In some cases of EGJOO, an esophagram is useful when manometry does not provide a conclusive diagnosis or if EGJOO in the supine position may be a false positive [67]. It should also be performed in patients with an inconclusive achalasia diagnosis presenting with dysphagia [6]. For other peristalsis disorders, the esophagogram may support the diagnosis, such as in absent contractility with borderline IRP values to rule out type I achalasia or to demonstrate poor bolus transit in IEM [6, 67].

Finally, evidence from some studies suggests that esophagogram with tablet test is more sensitive for detecting subtle esophageal strictures and obstructions, while liquid barium is better for general motility and large structural abnormalities. However, due to limited availability of TBE, it is not possible to definitively recommend the best approach for clinical practice based on different contexts [222, 223].

Statement 3.9 UEG/ESNM recommends the use of FLIP as an adjunctive diagnostic test in patients with dysphagia and inconclusive diagnoses from previous explorations including upper endoscopy and high‐resolution esophageal manometry.

Level of evidence: moderate, grade of recommendation: strong

Statement endorsed, overall agreement: 85% (A+ 44%, A 41%, A− 9%, D− 3%, D 3%, D+ 0%)

**Summary of evidence**

The FLIP is a technique that measures the mechanical properties of the esophagus, providing new additional information that complements the results of classical assessment of esophageal pressure measurements with manometry and bolus transit with radiology or impedance [224].

In patients with achalasia, an abnormally reduced esophagogastric junction distensibility index (EGJ‐DI) compared to healthy controls has been observed (typically < 2 mm^2^/mmHg) [225]. Moreover, clinical outcomes after treatment correlate with the increase of the EGJ‐DI obtained by the intervention [226, 227].

In patients with eosinophilic esophagitis, FLIP shows decreased distensibility of the esophageal body and esophago‐gastric junction compared to healthy controls [228]. A correlation between the rigidity of the esophageal body and the severity of dysphagia has been demonstrated in one follow‐up study [229].

In one study of 13 patients with severe dysphagia and bolus retention at timed barium esophagogram, but with absent contractility and normal LES‐relaxation at manometry, a low UEG‐DI was found compared to historic controls. Symptomatic improvement was associated with an increment in the UEG‐DI after treatment, supporting the use of FLIP in patients with an inconclusive diagnosis of achalasia [230].

In a retrospective study of 34 patients diagnosed as EGJOO, 7 patients were identified with normal EGJ‐DI, and 18 patients with an EGJ‐DI < 2 mm^2^/mmHg. There was an excellent correlation in all cases with bolus retention at radiology and with the clinical outcome. Hence, the 7 patients with normal EGJ‐DI improved dysphagia with conservative management, and 9 patients with low UEG‐DI underwent achalasia‐type treatment, with improved Eckardt score in 7 of these 9 treated patients [231]. Recently, a prospective trial of 15 patients with EGJOO and low EGJ‐DI that were treated with POEM has reported a clinical success in 93% of treated patients at 6 months [232]. Hence, the latest version of the Chicago classification of esophageal motility disorders recommends including the use of different maneuvers and adjuvant tests (like radiology and FLIP) to confirm the diagnosis of EGJOO [6].

In a single center study in 70 patients referred for dysphagia associated with different causes including achalasia, connective tissue disease, eosinophilic esophagitis and dysphagia post‐fundoplication, the authors reported that endoFLIP assessment independently resulted in a change in the management of 40% of patients, mostly a new or amended therapeutic procedure [233].

In a multicentre study, 40 patients referred for esophageal manometry (32/40 patients with dysphagia) underwent FLIP panometry during the endoscopic evaluation prior to manometry. The authors found an excellent correlation between the results of FLIP panometry and the results of the esophageal manometry, and they suggest that evaluation with FLIP panometry during endoscopy assessment of dysphagia could determine the need of further evaluation with esophageal manometry [234].

Statement 3.10 UEG/ESNM recommend the use of imaging studies when no endoscopic abnormalities are found and when the results of esophageal motility studies are atypical or inconclusive.

Level of evidence: moderate, grade of recommendation: strong

Statement endorsed, overall agreement: 82% (A+ 58%, A 24%, A− 12%, D− 3%, D 3%, D+ 0%)

**Summary of evidence**

A correct medical history is crucial when evaluating dysphagia and differentiating oropharyngeal from esophageal dysphagia. This distinction helps determine the appropriate diagnostic techniques. In challenging cases, multiple diagnostic tests may be required [6].

For esophageal dysphagia, imaging studies like barium swallow are useful when endoscopic abnormalities are absent and esophageal motility study results are atypical or equivocal [160]. Although computer tomography (CT) and magnetic resonance imaging (MRI) may detect morphological causes of dysphagia, barium studies remain the preferred modality. They are frequently requested for dysphagia evaluation and can assess both function and structural problems, sometimes more effectively than endoscopy [65]. Esophagography provides anatomical and functional information about the pharynx, esophagus, and gastroesophageal junction, including esophageal motility evaluation. A modified barium swallow gives information about swallowing function and remains the standard for evaluating the swallowing mechanism [235].

However, in cases of extrinsic compression (e.g., adjacent tumor, mass, adenopathy, or cardiovascular process), advanced imaging studies may be necessary for a definitive diagnosis [65]. CT, MRI, or endoscopic ultrasonography are required in pseudoachalasia evaluations [236]. For instance, thyroid ultrasonography or neck CT can confirm and determine the extent of thyroid disease. Chest CT helps assess disease extent in cases of extrinsic compression due to lung cancer and metastatic adenopathy. If clinically relevant cardiovascular esophageal compression is suspected on esophagram, CT or MR imaging may be necessary for a definitive diagnosis [232].

Endoscopic ultrasonography may be useful for patients with nondiagnostic endoscopy and high clinical suspicion for pseudoachalasia or to measure esophageal muscle thickness, but it is not routinely recommended for achalasia. In cases with manometry suggestive of EGJOO—a heterogeneous condition potentially due to anatomic factors (e.g., small hiatal hernia, extrinsic vascular compression), artifact, or incomplete achalasia expression—further evaluation with endoscopic ultrasound and/or CT is recommended to detect underlying conditions such as submucosal malignancies, infiltrative diseases like sarcoidosis, or vascular malformations [210, 237]. A retrospective study of 62 patients with esophageal motor disorders using standard radial endosonography revealed clinically relevant findings in a significant number of patients, which can alter clinical management. Variability in esophageal wall thickness identified by EUS may explain the observed variability in response to standard therapies for achalasia [237].

Scintigraphy can also be used to assess dysphagia; however, its utility as a screening tool for esophageal transit issues is limited due to several disadvantages, including the need to handle radioactive material, radiation exposure, poor anatomical definition compared to barium swallow, and the lack of well‐defined diagnostic criteria. Thus, it is rarely used in clinical practice [238]. For evaluating the pharynx and cervical esophagus, videofluoroscopy should be used to assess swallowing function and specifically check for aspiration or penetration [65]. Finally, new imaging techniques are emerging, such as real‐time MRI of swallowing, which offers a radiation‐free alternative to traditional methods like barium swallow and videofluoroscopy [239]. Real‐time MRI provides detailed anatomical and functional information about the oropharynx, esophagus, and surrounding structures, capturing dynamic sequences of the swallowing process [240].

Statement 3.11 UEG/ESNM do not recommend clinical swallow evaluation alone to definitely characterize OD.

Level of evidence: high, grade of recommendation: strong

Statement endorsed, overall agreement: 90% (A+ 66%, A 24%, A− 6%, D− 2%, D 0%, D+ 2%)

**Summary of evidence**

The purpose of screening is to identify persons at risk of OD (OD). Patients who are identified as being at risk are typically referred for further assessment: instrumental clinical assessment (e.g., fiberoptic endoscopic evaluation of swallowing [FEES] and videofluoroscopic swallowing study [VFSS]) and/or non‐instrumental clinical assessment (e.g., medical history taking, conducting a physical examination, and patient‐reported functional health status or dysphagia‐related quality of life) [15, 241].

In the process of clinical decision‐making in OD, aspiration is considered the factor most contributing to the severity of OD [242]. However, even though both “gold standard” assessments FEES and VFSS can diagnose aspiration (including silent aspiration) and other physiological problems in the pharyngeal phase of swallowing—whereas non‐instrumental assessment cannot –, other factors may influence choices of assessment methods: for example, radiation exposure, high associated costs, invasive assessment or restricted access to selected assessments. Also, given that OD is a multidimensional phenomenon, and most measures only focus on restricted aspects of OD, clinicians should include multiple measures if aiming to capture the full concept of OD [241]: for example, evaluation of the oral, laryngeal, and pharyngeal anatomy, physiology, and function (including cranial nerve examination); oral intake, nutritional status, and mealtime observations; and intervention trials (e.g., bolus modification, head and postural adjustments, and/or swallow maneuvers).

Statement 3.12 UEG/ESNM indicate FEES for (1) symptoms and signs of OD (OD), (2) selecting optimal dietary conditions, (3) designing a patient‐tailored OD treatment plan, and (4) verifying treatment outcomes and disease progression. UEG/ESNM do not recommend FEES in patients with bilateral complete nasal obstruction, a respiratory rate > 35/min, impaired consciousness, or refusal of oral food administration. UEG/ESNM do not suggest FEES in patients with severe agitation, possible inability to cooperate, and acute risk of vasovagal episode and bradycardia.

Level of evidence: moderate, grade of recommendation: weak

Statement endorsed, overall agreement: 90% (A+ 56%, A 34%, A− 3%, D− 5%, D 2%, D+ 0%)

**Summary of evidence**

FEES is an instrumental procedure used to evaluate swallowing function, including the surface anatomy and motility of the pharynx, tongue base, and larynx [243]. Alongside the VFSS, FEES is one of the gold standards for assessing swallow physiology and function in individuals with OD (OD) [243, 244]. It is a safe procedure with rare and minimal complications [245] and can be used in various settings, including bedridden patients, those with positioning difficulties, and in isolation units, intensive care units, or on mechanical ventilation [243, 244].

FEES contributes to diagnosing the underlying disease causing OD and prescribing a patient‐tailored treatment plan, including medical, surgical, and rehabilitative approaches [244, 246]. It can also assess dysphagia for medication [247] and verify that the swallowing pathophysiology or OD phenotype is coherent with the underlying diseases [248]. FEES has been used as a gold standard in validating several screening tools and monitoring the evolution and outcome of OD in different clinical conditions [249].

FEES is indicated for symptoms and signs of OD, such as increased meal duration, difficulties in managing saliva and secretions, and dysphagia for medication. It is also used to select optimal dietary conditions, design a patient‐tailored OD treatment plan, and verify treatment outcomes and disease progression over time [250]. FEES is contraindicated in patients with bilateral complete nasal obstruction, a respiratory rate > 35/min, impaired consciousness, or those who refuse oral food administration. Relative contraindications include severe agitation, possible inability to cooperate, and an acute risk of vasovagal episodes and bradycardia [243, 250].

Although FEES and VFSS are generally considered to be “gold standard” assessments in the evaluation of dysphagia, particularly silent aspiration, instrumental assessment is not always available, especially in rural and remote or resource poor regions. As a result, health care professionals will rely on alternative assessments including patient‐reported measures and non‐instrumental clinical swallowing evaluation [15].

Statement 3.13 UEG/ESNM recommend the use of VFSS, alongside FEES, as the method of choice for the instrumental assessment of oropharyngeal dysfunction.

UEG/ESNM indicate the use of VFSS to assess structural and functional abnormalities of the oral preparatory, oral transit, pharyngeal and esophageal phases of swallowing and to determine treatment strategies to minimize aspiration risk and increase swallow efficiency.

UEG/ESNM might consider the use of ultrasonograpy to evaluate muscles thickness and hyoid bone motion in patients with dysphagia, but the lack of standardized parameters limits its clinical utility.

Level of evidence: for VFSS: high, for US: low; grade of recommendation: for VFSS: strong, for US: weak

Statement endorsed, overall agreement: 95% (A+ 53%, A 42%, A− 3%, D− 0%, D 2%, D+ 0%)

**Summary of evidence**

Since bedside screening tests and clinical features have limitations in identifying oropharyngeal dysfunction, instrumental assessment is considered the gold standard for detecting swallowing disorders [15].

The VFSS, also known as the modified barium swallow, is a dynamic fluoroscopic imaging examination suitable for individuals of all ages experiencing swallowing difficulties. VFSS allows real‐time visualization and recording of the contrast bolus flow in relation to structural movement, timing, and coordination of the oral preparatory, oral transit, pharyngeal and/or esophageal phases of swallowing [15].

The VFSS serves both diagnostic and therapeutic purposes, including identifying structural abnormalities and impaired swallow function, determining suitable consistencies for oral intake [251], and testing various compensatory strategies such as postural or swallowing maneuvers [252]. As there is insufficient evidence to recommend specific measures as valid and reliable for interpreting videofluoroscopic findings [253], raters should use well‐defined and consensus‐based variables to ensure high intrarater and interrater reliability and accurate measurements [254]. The radiation dose of VFSS is acceptable for both patients and operators [255].

Compared to FEES, endoscopy tends to be slightly more sensitive than VFSS to detect aspiration and residues [256], although both methods have advantages and disadvantages. Especially if there is suspicion of oral or esophageal dysfunction following clinical evaluation, VFSS should be preferred over FEES [15].

Ultrasonography has been used to evaluate swallowing function, primarily focusing on muscle thickness and hyoid bone displacement. However, the absence of standardized protocols and outcomes hinders the assessment of the effectiveness of this technique in OD [257].

To date, no international consensus exists regarding which visuoperceptual or software‐based measures to use for analysis of FEES or VFSS video recordings [253], or about a core outcome set or agreed minimum set of outcomes that should be measured and reported in clinical trials of a specific disease or target population [15]. Therefore, current selections of assessments for use in clinical practice targeting different constructs, subject populations, and respondents, should be based on the robustness of measurement properties (i.e., psychometric domains reliability, validity, and responsiveness) [258], and feasibility criteria for implementation [241, 253, 259].

Statement 3.14 UEG/ESNM suggest the use of pharyngeal HRM in patients in whom the pharyngeal and UES function remains unclear and who present with abnormal swallow efficiency (residue) or safety (aspiration).

Level of evidence: moderate, grade of recommendation: weak

Statement endorsed, overall agreement: 85% (A+ 54%, A 31%, A− 11%, D− 2%, D 2%, D+ 0%)

**Summary of evidence**

For many years, HRM has been the standard method to assess motor function in the esophagus [201]. In the last decade, the applicability of HRM in the pharynx and UES, called Pharyngeal HRM (PHRM), has become clear [260]. Pharyngeal HRM is a catheter‐based assessment method that allows the evaluation of pharyngeal and UES motor function.

PHRM has been clinically used in the care of a wide variety of patients with OD across the lifespan [261, 262], including head & neck oncology [263], critically ill patients with or without tracheostomy, patients with Zenker's diverticulum [264], cervical spine fusion [265], obstructive sleep apnea [266], motor neuron disease [267], neurogenic dysphagia such as stroke, and Parkinson's disease [262, 263].

A global consensus statement has agreed on a protocol and core metrics for performing and analyzing PHRM [260].

The decision to perform HRIM after an initial VFSS, during VFSS (video‐HRIM), or as a stand‐alone test without VFSS depends on the individual patient circumstances. Aspiration risk and anatomical abnormalities should be studied either during VFSS or following a VFSS that establishes swallowing safety. Therefore, the use of VFSS can play an important and complementary role alongside HRM in patients at risk of aspiration or swallowing inefficiency. Patients studied using HRIM alone include those in whom clinical assessment suggests a low risk of aspiration, those who have previously undergone an MBS, and those requiring a repeat HRIM study to assess longitudinal changes or the impact of a therapeutic regimen or intervention [260].

The additional value of HRM compared to other instrumental assessment methods, such as FEES and VFSS, is that HRM can measure motor functions that lead to swallowing inefficiency (residue) or swallow safety issues (aspiration/penetration) observed on FEES and/or VFSS [260, 267]. This includes the characterization of pharyngeal contractility [267], UES relaxation, and bolus pressurization [260]. When impedance is added to the HRM measurement, UES opening can also be defined [267]. HRIM has significant potential to guide therapeutic interventions, such as swallowing exercises, UES dilation, Botox injection, or myotomy, and to objectively monitor these interventions or disease progression [262, 263, 266].

In conclusion, patients whose pharyngeal contractility and UES relaxation and their effects on UES opening remain uncertain may benefit from objective assessment using PHRM [260].

### 3.4 Section 4: Therapeutic Approaches

Statement 4.1 UEG/ESNM recognize that there is no clearly effective medical treatment able to improve motor function in patients with ineffective motility disorders. The management of these patients should be focused on treating the concomitant clinical scenario (e.g., GERD), rather than motor abnormalities.

Level of evidence: very low; grade of recommendation: weak

Statement endorsed, overall agreement: 89% (A+ 53%, A 36%, A− 11%, D− 0%, D 0%, D+ 0%)

**Summary of evidence**

Esophageal hypomotility involves defects in contraction vigor and/or peristalsis, with normal LES relaxation [6, 89]. Ineffective esophageal motility (IEM) and absent contractility (AC) are the corresponding manometric diagnoses according to the Chicago Classifications (CC). While AC often relates to pathological scenarios, IEM can appear in healthy individuals, leading to stricter diagnostic criteria in CC v4.0 [6]. Provocative tests like MRS, RDC, and solid swallow help identify IEM and assess motor esophageal reserve. MRS challenges contractile reserve, and abnormal responses suggest motility issues. RDC enhances sensitivity for detecting EGJ resistance, while solid swallow reveals dysfunctions and reduced reserve, especially in patients with solid food dysphagia [268].

The link between esophageal hypomotility and symptoms is controversial. Most patients present with GERD symptoms or dysphagia. Although some evidence connects IEM with abnormal acid exposure [269], some studies show no correlation between hypomotility and other symptoms, including dysphagia [107, 269]. Consequently, management should focus on treating symptoms rather than motor findings, particularly in GERD patients. PPI therapy is often the first option, and discontinuing drugs that reduce esophageal contraction vigor, such as calcium channel blockers and phosphodiesterase inhibitors, could be considered [270].

No medical treatments have been unequivocally shown to improve esophageal peristalsis. Conventional prokinetic agents like metoclopramide and domperidone do not enhance esophageal motor activity and have significant adverse effects. In one RCT, mosapride, a selective 5HT4 agonist, did not affect distal pressure wave amplitude in secondary peristalsis [271]. Conversely, on healthy volunteers was found that buspirone, a mixed partial 5HT‐1A agonist and dopamine D2 receptor antagonist, increased the amplitude and duration of distal esophageal pressure contractions and altered LES relaxation [272]. Karamanolis et al. reported that buspirone significantly increased LES resting pressure in systemic sclerosis patients, with a non‐significant trend towards higher esophageal contraction amplitude [273]. However, a small trial in IEM patients found buspirone not superior than placebo for improving HRM parameters or symptom scores [274], and in patients with IEM and GERD, buspirone did not outperform PPI alone in relieving dysphagia or enhancing HRM parameters [275]. Macrolide antibiotics like erythromycin and azithromycin have shown prokinetic properties in GERD patients, reducing reflux exposure and enhancing peristalsis, though side effects are not negligible [276]. Bethanechol, a muscarinic receptor agonist, has been used as a promotility agent for GERD, improving contraction and distal esophageal amplitude in both healthy volunteers and IEM patients [277], potentially alleviating dysphagia [278]. However, many patients discontinued due to intolerable side effects [278]. Prucalopride, a 5‐HT4 receptor enterokinetic agent, has shown promise in GERD patients with IEM by enhancing primary and secondary peristalsis [279, 280] and reducing esophageal acid exposure while accelerating gastric emptying in healthy controls [281].

Available evidence on esophageal hypomotility treatments is scarce. Larger, well‐conducted studies are needed to confirm current recommendations and potentially identify effective new prokinetics.

Statement 4.2 UEG/ESNM recognize that the medical management of esophageal hypercontractile motility or spasm requires a multifaceted approach. UEG/ESNM suggest the use of PPIs, calcium‐channel blockers, nitrates, phosphodiesterase type 5 inhibitors and incorporating other medications, such as antidepressants or peppermint oil, though endoscopic interventions may yield better outcomes in selected cases.

Level of evidence: very low—grade of recommendation: weak

Statement endorsed, overall agreement: 82% (A+ 42%, A 40%, A− 16%, D− 2%, D 0%, D+ 0%)

**Summary of evidence**

Hypercontractile motility and esophageal spasm are motility disorders that can present with dysphagia, chest pain, and gastroesophageal reflux symptoms. It is important to note that other causes of chest pain (e.g., cardiac chest pain) must be always ruled out. The pathophysiology may involve excessive cholinergic drive with temporal asynchrony of circular and longitudinal muscle contractions [282].

Medical management of esophageal hypercontractility or spasm is multifaceted. An empiric trial of a PPI is often the first line of therapy due to symptom overlap GERD [87, 282], although a definitive GERD diagnosis in these patients is rare, and the response to empirical PPI therapy is often unsatisfactory [283].

Calcium channel blockers, nitrates, and phosphodiesterase inhibitors have been used to reduce contraction vigor, but these have shown suboptimal symptomatic response [284].

Antidepressants, such as tricyclic antidepressants, selective serotonin reuptake inhibitors (SSRIs), and serotonin‐norepinephrine reuptake inhibitors (SNRIs), may help reduce the sensation of pain in the esophagus [285, 286]. Trazodone has been found to improve symptoms in patients with esophageal contraction abnormalities, such as distal esophageal spasm or hypercontractile esophagus, while SSRIs have been shown to lower chemical and mechanical sensitivity [285, 287]. Venlafaxine, an SNRI, lacks the antimuscarinic effects present in other antidepressants and has no affinity for brain histamine receptors; however, studies have shown venlafaxine's anti‐hyperalgesic properties and it can be used as a treatment option for non‐cardiac chest pain [286].

Peppermint oil, a smooth muscle relaxant, has been used to ease esophageal spasms [288]. A recent meta‐analysis examined treatment outcomes for Jackhammer Esophagus, finding that while medical treatments like calcium channel blockers, nitrates, PPIs, and peppermint oil showed some benefit, outcomes tended to be better with endoscopic interventions. However, conclusions are limited by methodological issues in the available research, as most studies were retrospective with significant heterogeneity in reported efficacy across different studies evaluating medical therapies [86].

In conclusion, the medical management of hypercontractile esophagus or esophageal spasm is complex and often requires a combination of lifestyle modifications and medications. The effectiveness of these treatments can vary, and more research is needed to develop safe and long‐lasting management strategies for this condition.

Statement 4.3 UEG/ESNM suggest that botulin toxin injection might be considered in the management of hypercontractile esophageal motility disorders. POEM could be justified in patients with concomitant esophagogastric junction disorder. In this later population, balloon dilation may also be an effective treatment, but further evidence is needed.

Level of evidence: very low—grade of recommendation: weak

Statement endorsed, overall agreement: 80% (A+ 43%, A 38%, A− 8%, D− 8%, D 5%, D+ 0%)

**Summary of evidence**

In the management of symptomatic patients with hypercontractile esophagus (HE), different endoscopic treatment options have been considered, including botulinum toxin injections, pneumatic dilation, and POEM. However, evidence supporting these treatments is limited, and well‐controlled studies are missing. It is important to note that pharmacological therapy is generally the first‐line approach for managing HE before considering endoscopic treatments, as it is less invasive and supported by broader clinical experience. Endoscopic interventions should be reserved for cases refractory to medical therapy, given the limited evidence and lack of well‐controlled studies supporting their efficacy [289, 290].

Schupack et al. observed that most HE patients showed clinical improvement or symptom resolution over an average follow‐up of 2.8 years without intervention. Specifically, 73% of HE patients reported improvement after a mean follow‐up of 36 weeks [291]. A Canadian meta‐analysis described treatment outcomes as generally satisfactory, while a French multi‐center study found them disappointing [86, 284]. The Canadian study noted a possible greater placebo effect with invasive procedures [86].

A randomized sham‐controlled trial by Mion et al. on botulinum toxin injections showed no significant difference in symptom improvement between the treatment and sham groups, indicating a considerable placebo effect [292]. On the other hand, the effect of botulinum toxin injections on patients with nonachalasia esophageal motility disorders was evaluated in a double‐blind, randomized trial that showed botulin significantly reduced dysphagia symptoms and stabilized weight loss compared to saline injections. The beneficial effects persisted for up to 12 months [293]. It is important to note that botulinum toxin should be injected at the level of the LESp and the distal third of the esophagus, as suggested by ESGE guidelines, while avoiding injections into the mid and proximal thirds [289, 290].

POEM appears to reduce esophageal contractility more effectively and any effect is likely to be long lasting. Only few studies including a limited number of patients are currently available, reporting the efficacy of POEM in patients with non‐achalasia spastic esophageal disorders [294, 295]. A significant manometry improvement and mean long‐term clinical success (defined as Eckard score < 3) were obtained in 93% of patients (72% with “nutcracker” esophagus and 88% with diffuse esophageal spasm). Long myotomy including LES is advisable to reduce symptoms from IEM or progression to achalasia [296]. A meta‐analysis by Khan et al. involving 179 patients found that POEM achieved a cumulative clinical success rate of 87% across all spastic esophageal disorders. Clinical success rates for type III achalasia, DES, and jackhammer esophagus were 92%, 88%, and 72%, respectively, with adverse event rates at 14%. POEM is thus concluded to be a possible treatment for spastic esophageal disorders [297].

Pneumatic dilation has been suggested as a treatment option for spastic esophageal disorders, showing some reported success [298]. However, patients benefiting from pneumatic dilation might actually have spastic achalasia or achalasia with esophageal compression. Accurate manometric classification is essential, as highlighted by Pandolfino et al., who found that pneumatic dilation had a lower treatment response in patients with spastic achalasia compared to those with achalasia accompanied by esophageal compression [115].

Statement 4.4 UEG/ESNM recognize that EGJOO often requires a tailored approach to management, with conservative management being the safest initial option. Structural EGJOO might considered to be treated with endoscopic or surgical interventions, while functional EGJOO might be considered for targeted therapy if symptoms are durable and worsening.

Level of evidence: very‐low, GRADE of recommendation: weak

Statement endorsed, overall agreement: 84% (A+ 63%, A 21%, A− 8%, D− 5%, D 3%, D+ 0%)

**Summary of evidence**

EGJOO is a manometrically defined condition that can be distinguished from achalasia subtypes. While EGJOO presents with a raised IRP, esophageal body motility remains intact. This condition shows significant variability in presentation, clinical manifestations, relevance, and natural history, complicating treatment decisions. In many instances, EGJOO is minimally symptomatic, may improve without specific therapy, and might be a manometric artifact (especially if studies are performed in the supine position only). However, EGJOO can also mimic achalasia with similar symptoms and respond well to achalasia‐type therapies. Some cases show progression towards achalasia, suggesting that in some cases EGJOO might be a variant or early form of achalasia [6, 109]. Notably, the revision of the EGJOO diagnosis criteria in the Chicago Classification version 4 has significantly reduced the prevalence of this disorder, making it challenging to assess the response to previously recommended treatments [299].

EGJOO can be categorized into four types to guide treatment: mechanical obstruction, medication‐related, artifact, and idiopathic non‐relaxing LES. Mechanical obstruction, accounting for up to 75% of cases, includes structural abnormalities like post‐surgery changes, small hiatus hernia, and submucosal lesions, or mucosal diseases like eosinophilic esophagitis and peptic strictures. The remaining 25% of cases are due to medication, artifact, or unexplained idiopathic causes. Opiate‐induced non‐relaxing EGJ makes up 10%–30% of functional EGJOO cases in some series [300].

Structural EGJOO should be treated with endoscopic or surgical interventions [300]. Strictures require dilation, slipped or distorted wraps may need surgical correction, and eosinophilic esophagitis follows specific therapeutic algorithms [301, 302]. Functional EGJOO, after excluding manometric artifacts or structural pathologies, can be considered for targeted therapy if symptoms are durable and worsening [303]. Treatments include smooth muscle relaxants, acid suppressive therapy, botulinum toxin, standard or pneumatic dilation, and myotomy (POEM or Heller). Response rates for botulinum toxin, pneumatic dilation, and POEM are over 60% [232], while no therapy or acid‐reducing therapy also shows high success rates, especially for medication‐related or artifact cases [180, 304].

This highlights the importance of not rushing into invasive treatments for functional EGJOO, allowing time for spontaneous symptom regression or further confirmation of the condition. Each case should be considered individually, making an algorithmic approach challenging [291]. However, the safest strategy is to start with conservative management before considering irreversible or invasive therapies.

Statement 4.5 UEG/ESNM recognize that in patients with functional dysphagia antidepressants or neuromodulators might be considered, but there is lack of data to recommend their use.

Level of evidence: very‐low, GRADE of recommendation: weak

Statement endorsed, overall agreement: 89% (A+ 51%, A 38%, A− 8%, D− 0%, D 3%, D+ 0%)

**Summary of evidence**

Functional dysphagia is characterized by a sensation of abnormal food passage through the esophagus in absence of organic causes, GERD, EoE or major motor disorders [153]. Since establishing the first Rome criteria, antidepressants have been the mainstay of treatment for functional esophageal disorders, presumably through their pain‐modulatory effect. However, the list of different types of antidepressants that have been proposed to improve symptoms in patients with these disorders has expanded over the years and currently includes tricyclic antidepressants, selective SSRIs, trazodone, and serotonin‐norepinephrine reuptake inhibitors [305]. Trazodone has modified symptoms in patients with esophageal contraction abnormalities in a small, controlled trial [287]. Imipramine has been shown to decrease pain in healthy males and noncardiac chest pain patients [306]. Citalopram has been shown to raise stimuli thresholds and benefit patients with esophageal hypersensitivity and functional heartburn [285]. Although psychiatric comorbidities are common in these patients, the effects of these drugs appear to be independent of psychiatric effects and do not appear to affect esophageal motility [287, 306].

In the literature, there is lack of evidence that neuromodulators or antidepressants are efficient in the treatment of functional dysphagia. A recent systematic literature review including randomized controlled trials showed that antidepressants cause visceral analgesia in functional esophageal disorders; however, this systematic literature review did not identify any RCTs describing the use of antidepressants in the context of functional dysphagia, or any other evidence for the use of antidepressants for dysphagia. Moreover, authors of this review emphasized that despite the wide use of antidepressants in clinical practice, there is no evidence for their value in most esophageal disorders, including traditional functional esophageal disorders. Thus, it appears that their use is driven primarily by expert opinion as well as a lack of other effective and well‐tolerated therapeutic strategies [307].

In a very recent small size (10 patients) RCT published after this systematic literature review, a 2 weeks treatment of buspirone (a 5‐HT1a receptor agonist) was not superior to the placebo in term of improvement of dysphagia symptoms in patients with functional dysphagia [274].

Although there is no data in the literature about the efficacy of abovementioned medications on FD, use of these medications in daily practice depends on the extrapolation of available limited data regarding functional esophageal diseases other than FD.

Statement 4.6 UEG/ESNM recognize that in patients with functional dysphagia, there is lack of data to recommend the use of complementary or alternative therapies.

Level of evidence: very‐low, GRADE of recommendation: weak

Statement endorsed, overall agreement: 95% (A+ 72%, A 23%, A− 5%, D− 0%, D 0%, D+ 0%)

**Summary of evidence**

The management of functional dysphagia focuses on therapies addressing visceral hypersensitivity and hypervigilance. Factors such as acid reflux, dietary habits, and psychiatric conditions contribute to esophageal symptoms [308]. Pharmaceutical or talk‐based treatments for esophageal hypersensitivity have largely concentrated on functional chest pain, leaving functional dysphagia less studied [101].

Research indicates that patients with normal esophageal motility or minor abnormalities often experience spontaneous improvement without medical interventions [309]. Case series suggest positive outcomes with various treatments, including tricyclic antidepressants, trazodone, and selective serotonin reuptake inhibitors, and endoscopic empiric dilatation, although no randomized controlled trials validate these treatments [307, 310]. Complementary therapies such as hypnotherapy and Johrei have been explored in related disorders, but robust trials specifically for functional dysphagia are lacking [311].

Brain‐gut behavioral therapies (BGBT) offer potential non‐pharmacologic interventions for functional esophageal symptoms. These include self‐management programs, GI‐focused cognitive behavioral therapy (CBT), gut‐directed hypnotherapy, mindfulness‐based interventions, and psychodynamic‐interpersonal therapy [101]. Among these, only hypnotherapy has shown beneficial effects in individual case reports [312].

Given the limited scientific evidence, treatment for functional dysphagia is primarily supportive, involving reassurance, dietary modifications, and proper swallowing techniques [313]. Analogous to treatments for functional chest pain, some patients may benefit from acid suppression, tricyclic azizantidepressants, and BGBT [98, 309, 313].

In summary, there is a lack of high‐quality data to recommend complementary or alternative therapies for functional dysphagia. Supportive care remains the primary management approach, with potential benefits from acid suppression and behavioral therapies. Well‐designed trials are needed to establish validated treatment modalities for this condition.

Statement 4.7 UEG/ESNM consider the use of swallow therapy for OD to improve swallowing mechanics, reduce symptoms, and enhance quality of life. Swallow therapy could be more effective when using validated assessment tools, consistent treatment parameters, and considering long‐term follow‐up.

Level of evidence: low—grade of recommendation: weak

Statement endorsed, overall agreement: 90% (A+ 54%, A 36%, A− 2%, D− 5%, D 0%, D+ 3%)

**Summary of evidence**

Following a diagnosis of OD, swallow therapy may be implemented to improve biomechanical impairments and the pathophysiology of swallowing, reduce clinical symptoms of dysphagia, and ultimately enhance swallow‐related quality of life.

Several randomized controlled trials (RCTs) have explored various interventions, such as pharyngeal electrical stimulation in people with acute and chronic stroke [314], respiratory muscle strength training in people with subacute and chronic stroke [315], and repetitive transcranial magnetic stimulation (rTMS) in people with acute, subacute, and chronic stroke [316] and advancing Parkinson's disease [317]. Transcranial direct current stimulation (tDCS) was employed in people with subacute and chronic stroke [318], neuromuscular electrical stimulation (NEMS) was used in people with acute stroke [317] and subacute/chronic stroke [319], and oromotor exercises were implemented during chemoradiotherapy for people with head and neck cancer [320] and stroke [321]. The Shaker maneuver, targeting impaired upper esophageal sphincter opening, was investigated in various aetiologies [322]. In addition, McNeill Dysphagia Therapy was studied in people with subacute stroke [323] and modified balloon dilatation therapy was investigated in chronic brainstem stroke [324]. Laryngeal recalibration therapy (LRT) and IQORO are both treatments aimed at improving swallowing function. LRT focuses on retraining the laryngeal muscles to enhance voice and swallowing efficiency. IQORO, a neuromuscular training device, strengthens muscles in the throat and esophagus, helping treat dysphagia, acid reflux, and snoring by targeting the root cause. Both therapies offer non‐invasive ways to restore normal function in patients with swallowing disorders [325, 326].

Overall, although many studies found statistically significant improvements in neurophysiological, biomechanical, clinical, functional, patient‐reported, or quality of life‐related outcomes, there were often no statistically significant differences between the treatment and control groups among several different therapies, which impacted the overall strength of this body of research [315, 316, 317, 318, 319]. Unfortunately, there is low‐quality evidence overall to support swallow therapy in OD, mostly due to the following limitations: small sample sizes impacting statistical significance, differences across studies making it impossible to combine results from treatment groups (such as sample heterogeneity), a wide variety of assessment tools used (often not validated and/or lacking instrumental assessment), different protocols utilized (intensity, duration, frequency, dose for stimulation, location of electrodes, parameters, etc.), lack of follow‐up, and limited blinding resulting in a high risk of bias [315, 316].

A recent systematic review by Speyer et al., highlight the promising effects of behavioral interventions in improving outcomes for people with OD. Although many trials report significant improvements in clinical and functional outcomes, limitations such as small sample sizes, heterogeneous study designs, and lack of standardized assessment tools remain prevalent. To enhance the quality of evidence, further research is needed with larger, more homogeneous samples and consistent, validated outcome measures [327].

Statement 4.8 UEG/ESNM might consider surgical treatment for OD primarily based on the etiology. It is most appropriate for correcting anatomical abnormalities.

Level of evidence: very‐low, grade of recommendation: weak

Statement endorsed, overall agreement: 87% (A+ 49%, A 38%, A− 0%, D− 10%, D 3%, D+ 0%)

**Summary of evidence**

Surgical intervention can be effective in the management of OD, particularly when the condition stems from anatomical abnormalities. Malignant obstructive lesions should be staged and managed according to current guidelines with regards to localization, TNM classification, anatomopathological findings, and the patient's overall health and preferences [328]. Treatment consists of radiotherapy, chemotherapy, targeted therapy, surgery, or a combination. In general, there is a clear indication for surgery in resectable tumors in fit patients [328, 329].

Zenker's diverticulum is a benign anatomical abnormality which can be managed surgically using a diverticulopexy combined with a myotomy. The procedure is safe and efficient. Compared with an endoscopic treatment, surgery results in lower symptom recurrence, but a longer length of procedure and hospitalization, slower diet introduction, and higher rates of complications [330]. Minimally invasive techniques such as Zenker's peroral endoscopic myotomy offer a less invasive alternative for treating Zenker's diverticulum with promising outcomes in reducing symptom recurrence and hospital stays [331].

Surgical therapy in functional OD consists of an open or endoscopic laser myotomy. It can be performed safely. Treatment success can be achieved in selected patients [332, 333]. Selection criteria for this may include: intact voluntary initiation of swallowing, adequate propulsive force generated by the tongue and pharyngeal constrictors, videofluoroscopic demonstration of obstruction to bolus flow at the piriform sinus, manometric evidence of inadequate UES relaxation, and relatively favorable prognosis of the underlying disease [332, 333].

Endoscopic laser cricopharyngeal myotomy is a viable alternative to classic transcervical cricopharyngeal myotomy with equivalent outcomes and comparable if not less morbidity [334].

In comparison to endoscopic dilation, myotomy of the UES led to similar initial improvement in swallow function for patients with primary idiopathic cricopharyngeal bar; however, dilation is more likely to provide temporary benefit [332]. For patients with cricopharyngeal bar, cricopharyngeal POEM is emerging as a promising endoscopic technique, offering minimally invasive treatment with encouraging results for long‐term symptom improvement [335].

Statement 4.9 In patients with post‐stroke dysphagia or due to other neurogenic diseases, UEG/ESNM suggest that treatment with neuromodulatory techniques should preferably be conducted within a clinical trial setting.

For post‐stroke dysphagia: level of evidence: moderate, grade of recommendation: weak

For other dysphagic profiles: level of evidence: very low, grade of recommendation: weak

Statement endorsed, overall agreement: 85% (A+ 48%, A 37%, A− 10%, D− 5%, D 0%, D+ 0%)

**Summary of evidence**

There is currently a large number of systematic reviews with or without meta‐analysis in the literature regarding the use of neuromodulatory treatments in dysphagic patients [336, 337, 338, 339, 340, 341, 342, 343, 344, 345]. The clinical focus for the use of neuromodulatory techniques is post‐stroke dysphagia, followed by neurogenic dysphagia as presented in parkinsonian syndromes (PS). The neuromodulatory techniques presented are repetitive transcranial magnetic stimulation (rTMS), direct transcranial current stimulation (tDCS), neuromuscular electrical stimulation (NMES), peripheral magnetic stimulation (PMS), pharyngeal electrical stimulation (PES), and deep brain stimulation (DBS—only in PS) [338, 339, 340, 341, 342].

Even though there is a large number of studies, it is difficult to draw a valid conclusion on the efficacy of the neuromodulatory techniques in dysphagia [346]. For dysphagia post‐stroke, the data is highly heterogeneous, and the subgroup analysis rarely included stroke type or clinical characteristics of the dysphagic patients [343, 344]. The outcomes of the systematic reviews targeting each of the aforementioned treatments were contradictory to an extent, with a number of researchers proposing combined rTMS or tDCS with behavioral treatments rather than solely brain stimulation [341, 343]. There is high variability in terms of applied protocols (site of lesion, frequency of the stimulation, time since stroke, repeated session, etc.) [338, 339, 340, 341, 342, 343].

Peripheral neuromodulatory techniques, such as NMES, PES, or PMS, have all been applied to post‐stroke dysphagia, showing moderate (PES) to good (NMES) results [338, 340, 343]. Even though NMES has shown better results when combined with traditional therapy, systematic appraisal of the literature showed high heterogeneity in protocols and experimental variables, as well as inconsistent reporting [337, 341]. DBS in PS is shown to have beneficial effects at specified frequencies and site of electrode placement in the subcortical areas [339]. The effect of peripheral interventions on the central nervous system remains questionable, leaving this aspect a gray area in this context.

Further high‐quality research is necessary in order to clarify which stimulation protocols, parameters, and therapy settings are the most beneficial for certain patient groups and degrees of impairment.

## 4 Summary of Recommendation

Questions, statements, and recommendations on the dysphagia guidelines by the UEG/ESNM are detailed in Table 3.

**TABLE 3:** Questions, statements, and recommendations on the dysphagia guidelines.

Section and number	Statement/recommendation	Endorsement	Level of evidence	Recommendation	Agreement
Statement 1.1	UEG/ESNM propose the following definition of ED: A difficulty swallowing generally sensed retrosternally and related to the esophagus or its lower esophageal sphincter (LES) caused by anatomical, mechanical or functional abnormalities	Yes	Very low	Expert opinion	87% A+ 69%, A 18%, A− 8%, D− 0%, D 5%, D+ 0%
Statement 1.2	Oropharyngeal dysphagia is defined as a swallowing dysfunction that causes impairment in moving bolus safely and effectively from the mouth through the pharynx into the esophagus. The main signs are divided into safety (airway penetrations and aspirations) or efficacy (prolonged swallow response, impaired labial seal, premature spillage in the pharynx, piecemeal deglutition, nasal regurgitation, oral or pharyngeal residue and impaired upper esophageal sphincter opening) swallowing impairments. The main accompanying symptoms include cough, choking, fear of eating, voice change, globus, prolonged meal duration, fatigue, and reduced pleasure of eating	Yes	Very low	Expert opinion	88% A+ 73%, A 15%, A− 5%, D− 2%, D 5%, D+ 0%
Statement 1.3	The general prevalence of dysphagia is estimated to be between 10% and 20%, depending on the definition used and the population included. Notably, no studies have assessed esophageal dysphagia prevalence as a separate concrete entity, therefore it is not possible to estimate its true prevalence	Yes	Very low	Expert opinion	87% A+ 50%, A 37%, A− 3%, D− 8%, D 2%, D+ 0%
Statement 1.4	The prevalence of oro‐pharyngeal dysphagia varies according to the target population. Elderly patients, patients with neurological or neurodegenerative diseases, and patients with head or neck diseases have the highest prevalence	Yes	Moderate	Good practice statement	90% A+ 76%, A 14%, A− 6%, D− 2%, D 0%, D+ 2%
Statement 1.5	Oropharyngeal dysphagia is associated with higher hospital stays and costs as well as higher probability of being transferred to post‐acute care facilities such as nursing homes. Moreover, the development of nutritional and respiratory complications, such as malnutrition and respiratory infections, has been associated with an independent increase in long‐term costs for follow‐up, especially in post‐stroke patients. Although data on esophageal dysphagia are limited, the condition has been associated with prolonged hospital stays, higher inpatient costs, and increased comorbidities and mortality	Yes	Moderate	Good practice statement	95% A+ 71%, A 24%, A− 3%, D− 0%, D 0%, D+ 2%
Statement 2.1	A wide range of systemic neuromuscular, rheumatological/immunological, endocrinological, and infectious diseases can be associated with esophageal and/or oropharyngeal dysphagia	Yes	Moderate	Good practice statement	82% A+ 69%, A 13%, A− 15%, D− 3%, D 0%, D+ 0%
Statement 2.2	Major motility disorders identified on HRM are generally associated with symptoms, primarily dysphagia	Yes	High	Strong	82% A+ 59%, A 23%, A− 5%, D− 3%, D 5%, D+ 5%
Statement 2.3	Spastic (premature) esophageal contraction is a mechanism underlying the symptoms of esophageal dysphagia	Yes	Low	Weak	90% A+ 62%, A 28%, A− 8%, D− 0%, D 2%, D+ 0%
Statement 2.4	Hypercontractile contraction is a mechanism underlying symptoms of esophageal dysphagia	Yes	Moderate	Weak	92% A+ 67%, A 25%, A− 8%, D− 0%, D 0%, D+ 0%
Statement 2.5	IEM is a mechanism underlying symptoms of esophageal dysphagia	Yes	Low	Weak	82% A+ 62%, A 20%, A− 8%, D− 5%, D 3%, D+ 2%
Statement 2.6	Esophageal hypersensitivity is a mechanism underlying symptoms of esophageal dysphagia	Yes	Very low	Weak	80% A+ 45%, A 35%, A− 13%, D− 3%, D 2%, D+ 2%
Statement 2.7	Decreased EGJ distensibility is a mechanism underlying symptoms of esophageal dysphagia	Yes	High	Strong	87% A+ 62%, A 25%, A− 10%, D− 0%, D 3%, D+ 0%
Statement 2.8	Impaired LES relaxation is a mechanism underlying symptoms of esophageal dysphagia achalasia apart	Yes	Moderate	Strong	95% A+ 67%, A 28%, A− 3%, D− 2%, D 0%, D+ 0%
Statement 2.9	Esophageal dysmotility may predisposes to disorders like epiphrenic diverticulum, candida esophagitis and strictures	Yes	Very low	Weak	87% A+ 62%, A 26%, A− 10%, D− 3%, D 0%, D+ 0%
Statement 2.10	The main phenotypes of patients with OD are older adults, patients with neurological and neurodegenerative diseases, patients with head and neck cancer (pre‐ or post‐treatment), and patients with anatomical/structural alterations. A less prevalent group includes patients with infectious or metabolic diseases, side effects of drugs, or iatrogenic conditions	Yes	High	Good practice statement	93% A+ 76%, A 17%, A− 7%, D− 0%, D 0%, D+ 0%
Statement 2.11	Patients with oropharyngeal dysphagia (OD) have impaired biomechanics of the oropharyngeal swallow response, including delayed total deglutition time, delayed time to laryngeal vestibule closure, delayed upper esophageal sphincter opening, and reduced bolus kinematics (propulsion force and bolus velocity). Neurophysiologically, they exhibit impairments in both afferent (sensory) and efferent (motor) pathways. Additionally, specific structural changes in the oral cavity, pharynx, larynx, and upper esophageal sphincter can lead to the development of structural OD	Yes	Moderate	Good practice statement	86% A+ 67%, A 19%, A− 0%, D− 3%, D 5%, D+ 6%
Statement 2.12	Oropharyngeal dysphagia is associated with serious nutritional and respiratory complications, including malnutrition, dehydration, and respiratory infections such as aspiration pneumonia. These complications are linked to poor health outcomes, including hospital readmissions and increased morbidity and mortality	Yes	Moderate	Good practice statement	90% A+ 76%, A 14%, A− 7%, D− 0%, D 0%, D+ 3%
Statement 2.13	Dysphagia and anxiety/depression can be associated conditions. While this link is well addressed in the case of oropharyngeal dysphagia, data on esophageal dysphagia are scarce	Yes	Very low	Expert opinion	87% A+ 44%, A 44%, A− 10%, D− 3%, D 0%, D+ 0%
Statement 3.1	UEG/ESNM recommends careful focused history taking, although it is insufficient to differentiate between esophageal and oropharyngeal dysphagia	Yes	Very low	Expert opinion	85% A+ 65%, A 20%, A− 10%, D− 5%, D 0%, D+ 0%
Statement 3.2	UEG/ESNM recommend upper gastrointestinal endoscopy to evaluate esophageal dysphagia in all adult patients. Upper gastrointestinal endoscopy also plays a pivotal role in cases of acute onset of inability to swallow, which suggests a food impaction in the esophagus	Yes	Low	Strong	92% A+ 79%, A 13%, A− 5%, D− 0%, D 3%, D+ 0%
Statement 3.3	The yield of EGD for diagnosing structural abnormalities underlying esophageal dysphagia varies among studies. However, structural abnormalities are well visualized on EGD. Erosive reflux disease that can progress to peptic strictures and EoE, which can be associated with fibrotic narrowing of the esophagus, are common. Idiopathic fibrosis (Schatzki ring), complications of hiatus hernia, as well as malignancies, are less frequent	Yes	Low	Strong	93% A+ 63%, A 30%, A− 3%, D− 0%, D 2%, D+ 2%
Statement 3.4	UEG/ESNM suggest that esophageal biopsy sampling should be obtained in all patients with dysphagia, especially if clinical characteristics (male, young age, history of atopy or allergy) are suggestive for eosinophilic esophagitis even if macroscopic endoscopy results normal	Yes	Low	Strong	97% A+ 76%, A 22%, A− 3%, D− 0%, D 0%, D+ 0%
Statement 3.5	Edema, rings, exudates, furrows and stenosis are the most relevant endoscopic findings of EoE	Yes	High	Strong	95% A+ 71%, A 24%, A− 3%, D− 2%, D 0%, D+ 0%
Statement 3.6	UEG/ESNM recommend the use of HRM in esophageal dysphagia as HRM is the most important technology focused on assessing esophageal motor function causing dysphagia in endoscopy‐negative dysphagia patients	Yes	High	Strong	95% A+ 74%, A 21%, A− 5%, D− 0%, D 0%, D+ 0%
Statement 3.7	UEG/ESNM suggest the use of pH and impedance‐pH monitoring as it might provide information on the possible presence of gastroesophageal reflux disease or esophageal hypersensitivity	Yes	Moderate	Weak	87% A+ 56%, A 31%, A− 3%, D− 3%, D 8%, D+ 0%
Statement 3.8	In patients with esophageal dysphagia, UEG/ESNM suggest a barium esophagram as complementary investigation to improve the diagnosis of structural and functional esophageal disease when endoscopy and HRM did not fully explain symptoms	Yes	Moderate	Strong	97% A+ 72%, A 26%, A− 3%, D− 0%, D 0%, D+ 0%
Statement 3.9	UEG/ESNM recommends the use of EndoFLIP as an adjunctive diagnostic test in patients with dysphagia and inconclusive diagnoses from previous explorations including upper endoscopy and high‐resolution esophageal manometry	Yes	Moderate	Strong	85% A+ 44%, A 41%, A− 9%, D− 3%, D 3%, D+ 0%
Statement 3.10	UEG/ESNM recommended the use of imaging studies when no endoscopic abnormalities are found and when the results of esophageal motility studies are atypical or inconclusive	Yes	Moderate	Strong	82% A+ 58%, A 24%, A− 12%, D− 3%, D 3%, D+ 0%
Statement 3.11	UEG/ESNM do not recommend clinical swallow evaluation alone to definitely characterize oropharyngeal dysphagia	Yes	High	Strong	90% A+ 66%, A 24%, A− 6%, D− 2%, D 0%, D+ 2%
Statement 3.12	UEG/ESNM indicate FEES for (1) symptoms and signs of oropharyngeal dysphagia (OD), (2) selecting optimal dietary conditions, (3) designing a patient‐tailored OD treatment plan, and (4) verifying treatment outcomes and disease progression. UEG/ESNM do not recommend FEES in patients with bilateral complete nasal obstruction, a respiratory rate > 35/min, impaired consciousness, or refusal of oral food administration. UEG/ESNM do not suggest FEES in patients with severe agitation, possible inability to cooperate, and acute risk of vasovagal episode and bradycardia	Yes	Moderate	Weak	90% A+ 56%, A 34%, A− 3%, D− 5%, D 2%, D+ 0%
Statement 3.13	UEG/ESNM recommend the use of videofluoroscopic swallow study (VFSS), alongside fiberendoscopic evaluation of swallowing (FEES), as the method of choice for the instrumental assessment of oropharyngeal dysfunction	Yes	For VFSS: High For US: Low	For VFSS: Strong For US: Weak	95% A+ 53%, A 42%, A− 3%, D− 0%, D 2%, D+ 0%
	UEG/ESNM indicate the use of VFSS to assess structural and functional abnormalities of the oral preparatory, oral transit, pharyngeal and esophageal phases of swallowing and to determine treatment strategies to minimize aspiration risk and increase swallow efficiency				
	UEG/ESNM might consider the use of ultrasonograpy to evaluate muscles thickness and hyoid bone motion in patients with dysphagia, but the lack of standardized parameters limits its clinical utility				
Statement 3.14	UEG/ESNM suggest the use of pharyngeal high‐resolution manometry in patients in whom the pharyngeal and UES function remains unclear and who present with abnormal swallow efficiency (residue) or safety (aspiration)	Yes	Moderate	Weak	85% A+ 54%, A 31%, A− 11%, D− 2%, D 2%, D+ 0%
Statement 4.1	There is no clearly effective medical treatment able to improve motor function in patients with ineffective motility disorders. The management of these patients should be focused on treating the concomitant clinical scenario (e.g., GERD), rather than motor abnormalities	Yes	Very low	Weak	89% A+ 53%, A 36%, A− 11%, D− 0%, D 0%, D+ 0%
Statement 4.2	The medical management of esophageal hypercontraction or spasm requires a multifaceted approach. UEG/ESNM suggest the use of PPIs, calcium‐channel blockers, nitrates, phosphodiesterase type 5 inhibitors and incorporating other medications, such as antidepressants or peppermint oil, though endoscopic interventions may yield better outcomes in selected cases	Yes	Very low	Weak	82% A+ 42%, A 40%, A− 16%, D− 2%, D 0%, D+ 0%
Statement 4.3	UEG/ESNM suggest that botulin toxin injection might be considered in the management of hypercontractile esophageal motility disorders. POEM could be justified in patients with concomitant esophagogastric junction disorder. In this later population, balloon dilation may also be an effective treatment, but further evidence is needed	Yes	Very low	Weak	80% A+ 43%, A 38%, A− 8%, D− 8%, D 5%, D+ 0%
Statement 4.4	EGJOO often requires a tailored approach to management, with conservative management being the safest initial option. Structural EGJOO might considered to be treated with endoscopic or surgical interventions, while functional EGJOO might be considered for targeted therapy if symptoms are durable and worsening	Yes	Very low	Weak	84% A+ 63%, A 21%, A− 8%, D− 5%, D 3%, D+ 0%
Statement 4.5	In patients with functional dysphagia antidepressants or neuromodulators might be considered, but there is lack of data to recommend their use	Yes	Very low	Weak	89% A+ 51%, A 38%, A− 8%, D− 0%, D 3%, D+ 0%
Statement 4.6	In patients with functional dysphagia, there is lack of data to recommend the use of complementary or alternative therapies	Yes	Very low	Weak	95% A+ 72%, A 23%, A− 5%, D− 0%, D 0%, D+ 0%
Statement 4.7	UEG/ESNM consider the use of swallow therapy for oropharyngeal dysphagia to improve swallowing mechanics, reduce symptoms, and enhance quality of life. Swallow therapy could be more effective when using validated assessment tools, consistent treatment parameters, and considering long‐term follow‐up	Yes	Low	Weak	90% A+ 54%, A 36%, A− 2%, D− 5%, D 0%, D+ 3%
Statement 4.8	UEG/ESNM might consider surgical treatment for oropharyngeal dysphagia primarily based on the etiology. It is most appropriate for correcting anatomical abnormalities. For functional abnormalities, UEG/ESNM might suggest considering surgery only after conservative treatments have failed	Yes	Very low	Weak	87% A+ 49%, A 38%, A− 0%, D− 10%, D 3%, D+ 0%
Statement 4.9	In patients with post‐stroke dysphagia or due to other neurogenic diseases, UEG/ESNM suggest that treatment with neuromodulatory techniques should preferably be conducted within a clinical trial setting	Yes	For post‐stroke dysphagia: Moderate For other dysphagic profiles: Very low	Weak	85% A+ 48%, A 37%, A− 10%, D− 5%, D 0%, D+ 0%

**FIGURE 1:**
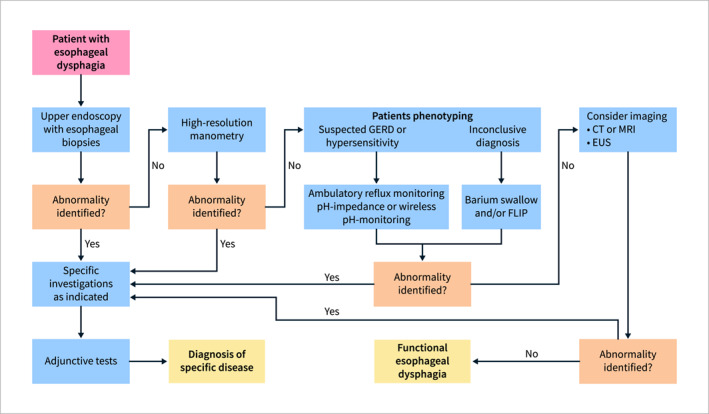
Diagnostic approach for esophageal dysphagia. CT, computer tomography; EUS, endoscopic ultrasound; FLIP, functional lumen imaging probe; GERD, gastroesophageal reflux disease; MRI, magnetic resonance imaging.

Figure 1 schematically summarizes the main findings regarding the diagnostic approach to esophageal dysphagia. The consensus supports the performance of EGD with multiple esophageal biopsies as the initial diagnostic modality, mainly to search for anatomical and mucosal lesions. The next diagnostic modality recommended by the consensus is HRM, a modality that evaluate the esophageal motility as well as the LES function. In case of negative findings at HRM, the next step is patient's symptoms phenotyping to GERD/hypersensitive esophagus or dysphagia with inconclusive diagnosis. In these clinical scenarios, either ambulatory reflux monitoring or FLIP/Barium swallows are performed. In patients with persistent symptoms and additional testing might be required, such as imaging (CT, MRI, EUS). Functional dysphagia is the diagnosis in case of negative diagnostics.

**FIGURE 2:**
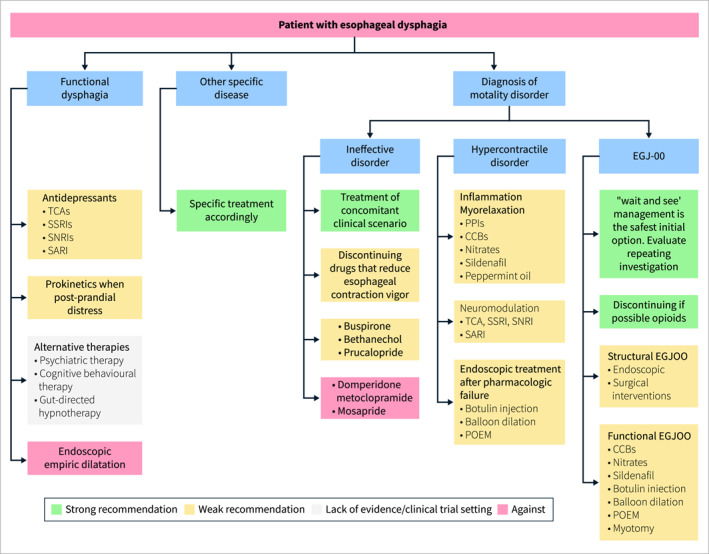
Therapeutic approach for esophageal dysphagia. CCBs, calcium channel blockers; EGJ‐OO, esophagogastric junction outflow obstruction; POEM, peroral endoscopic myotomy; PPIs, proton pump inhibitors; SARI, serotonin antagonist and reuptake inhibitors; SNRIs, serotonin‐norepinephrine reuptake inhibitors; SSRIs, selective serotonin reuptake inhibitors; TCAs, tricyclic antidepressants.

Figure 2 illustrates recommendations regarding treatment schematically. A strong consensus was achieved for the “wait and see” approach and for the discontinuation of opioids for patients with EGJOO. Very weak/low recommendation was reached regarding surgical and endoscopic treatment for structural EGJOO as well as for CCB, nitrates, sildenafil, botulinum toxin, balloon dilation and myotomy—for the treatment of functional EGJOO. Moreover, weak/low recommendation was reached for PPI, CCB, nitrates, sildenafil, TCA, SSRI, NSRI, SARI, peppermint oil as well as botulinum toxin, balloon dilation and myotomy—for the treatment of hypercontractile esophagus. For patients with ineffective esophageal motility, a strong consensus was achieved for the treatment of concomitant clinical scenario such as GERD. Moreover, weak/low recommendation was reached for buspirun, bethanechol and prucalopride. The consensus voted against the use of prokinetics such as donperidone, metoclopramide and mosapride. A strong consensus was achieved for the use of TCA, SSRI, NSRI, SARI‐ for the treatment of functional dysphagia.

**FIGURE 3:**
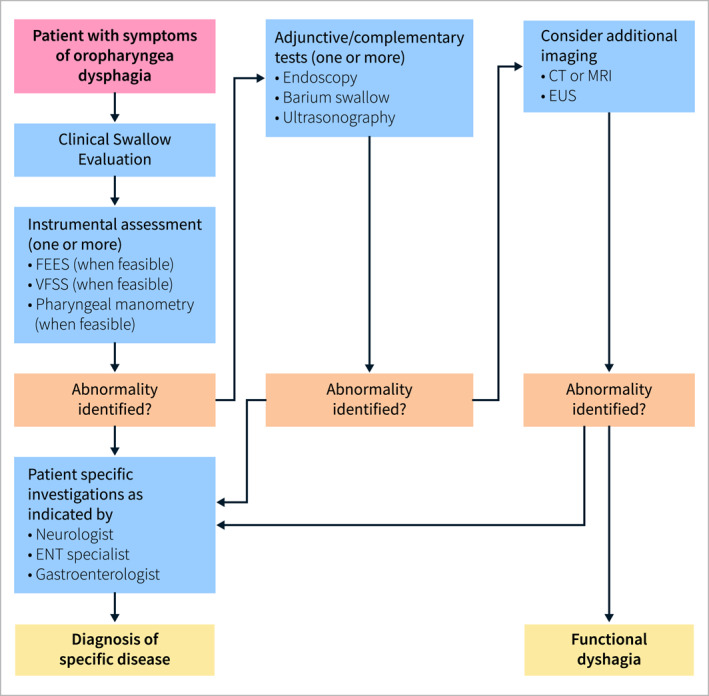
Diagnostic approach for oropharyngeal dysphagia. CT, computed tomography; ENT, ear, nose, and throat; EUS, endoscopic ultrasonography; FEES, fiberoptic endoscopic evaluation of swallowing; MRI, magnetic resonance imaging; VFSS, videofluoroscopic swallowing study.

Figure 3 schematically summarizes the main findings regarding the diagnostic approach to oropharyngeal dysphagia. The consensus recommends that an initial clinical swallow evaluation should be followed by instrumental assessments such as FEES, VFSS, or pharyngeal manometry (when feasible). Additional tests, including endoscopy, barium swallow, and imaging studies, may be conducted as necessary.

**FIGURE 4:**
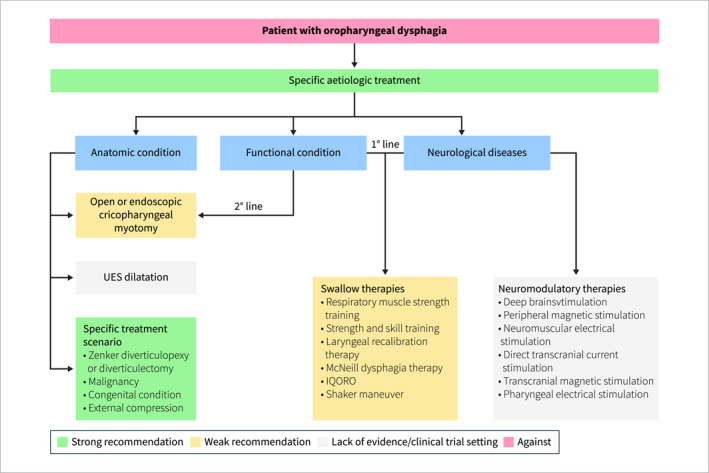
Therapeutic approach for oropharyngeal dysphagia. UES, upper esophageal sphincter.

Figure 4 illustrates recommendations regarding treatment schematically. The consensus supports the classification of oropharyngeal dysphagia based on the etiology. For anatomic conditions, a strong consensus was achieved for specific treatment scenario such as zenker diverticulopexy or diverticulectomy, malignancy, congenital condition and external compression. For neurological diseases, a very low/low recommendations were achieved for swallow therapies were is lack of evidence/clinical trial setting was reached by the consensus for neuromodulatory therapies. A very low/low recommendations were achieved for swallow therapies and Open/endoscopic cricopharyngeal myotomy for the treatment of oropharyngeal dysphagia with functional origin.

## 5 Conclusion

Dysphagia is a prevalent symptom in the general population, with a great impact on physical and psychological status. In recent years, major advances in dysphagia diagnosis and management have been achieved. This multinational and multidisciplinary group of experts applied a Delphi process to summarize and grade the current state of consensus on the diagnosis and treatment of esophageal and oropharyngeal dysphagia. The Consensus Group voted on various statements that may guide clinicians in the management of dysphagia in clinical practice. Future research should focus on refining diagnostic and therapeutic strategies through high‐quality randomized controlled trials, particularly in areas where current evidence is limited. Advances in diagnostic tools and novel endoscopic techniques are expected to significantly enhance the accuracy and efficiency of disease detection and management. Additionally, further studies are needed to evaluate long‐term outcomes of emerging therapeutic options and their integration into routine clinical practice.

## Author Contributions

All the authors contributed with data collection and analysis, writing of the manuscript, approving final version.

## Conflicts of Interest

Amir Mari, Francesco Calabrese, Greta Lorenzon, Bas Weusten, Jutta Keller, Pierfrancesco Visaggi, Ram Dickman, Jordi Serra, Nicola De Bortoli, Paola Iovino, Dan Dumitrascu, Mentore Ribolsi, Claudia Barber, Serhat Bor, Mark Fox, Rami Sweis, Vicente Lorenzo‐Zuniga, Filiz Akyuz, Matteo Ghisa, Altay Celebi, Fahmi Shibli, Rainer Dziewas, Ismail Hakkı Kalkan, Jan Tack, Pere Clavé, Silvia Carrion, Ivy Cheng, Noemi Tomsen, Omar Ortega, Sergio Marin Rubio, Nicole Pizzorni, Emilia Michou, Julie Regan, Nathalie Rommel, Martina Scharitzer, Olle Ekberg, Antonio Schindler, Renee Speyer, and Anna Gillman: no conflict of interest to declare; Andrea Pasta: Consultant for FenixPharma; Sabine Roman: Consultant for Dr Falk Pharma, Sanofi; Research support from Medtronic, Diversatek Healthcare; Elisa Marabotto: Consultant AlphaSigma, Dr. Falk; Daniel Pohl: Consultant and Speaker Medtronic; Shaheen Hamdy: Chief Scientific Officer of Phagenesis Ltd, and holds stocks/shares in that company; Frank Zerbib: Consultant for Medtronic, Dr Falk Pharma, Bioprojet, Sanofi, AstraZeneca, Bristol Myers Squibb; Edoardo V. Savarino: Speaker for Abbvie, Abivax, Agave, AGPharma, Alfasigma, Apoteca, Biosline, CaDiGroup, Celltrion, Dr Falk, EG Stada Group, Fenix Pharma, Galapagos, Johnson & Johnson, JB Pharmaceuticals, Innovamedica/Adacyte, Eli Lilly, Malesci, Mayoly Biohealth, Montefarco, Novartis, Omega Pharma, Pfizer, Rafa, Reckitt Benckiser, Sandoz, Sanofi/Regeneron, SILA, Sofar, Takeda, Tillots, Unifarco; has served as consultant for Abbvie, Agave, Alfasigma, Biogen, Bristol‐Myers Squibb, Celltrion, Dr. Falk, Eli Lilly, Fenix Pharma, Ferring, Giuliani, Grunenthal, Johnson & Johnson, JB Pharmaceuticals, Merck & Co, Nestlè, Pfizer, Reckitt Benckiser, Sanofi/Regeneron, SILA, Sofar, Takeda, Unifarco; he received research support from Bonollo, Difass, Pfizer, Reckitt Benckiser, Sanofi/Regeneron, SILA, Sofar, Unifarco, Zeta Farmaceutici.

## Supporting information

Table S1

## Data Availability

Data sharing not applicable to this article as no datasets were generated or analysed during the current study.

## References

[1] C. Adkins , W. Takakura , B. M. R. Spiegel , et al., “Prevalence and Characteristics of Dysphagia Based on a Population‐Based Survey,” Clinical Gastroenterology and Hepatology 18, no. 9 (2020): 1970–1979.e2, 10.1016/j.cgh.2019.10.029. 31669055 PMC7180111

[2] E. S. Dellon and I. Hirano , “Epidemiology and Natural History of Eosinophilic Esophagitis,” Gastroenterology 154, no. 2 (2018): 319–332.e3, 10.1053/J.GASTRO.2017.06.067. 28774845 PMC5794619

[3] P. Clavé and R. Shaker , “Dysphagia: Current Reality and Scope of the Problem,” Nature Reviews Gastroenterology & Hepatology 12, no. 12 (2015): 259–270, 10.1038/nrgastro.2015.49. 25850008

[4] P. Zuercher , C. S. Moret , R. Dziewas , and J. C. Schefold , “Dysphagia in the Intensive Care Unit: Epidemiology, Mechanisms, and Clinical Management,” Critical Care 23, no. 1 (2019): 103, 10.1186/S13054-019-2400-2. 30922363 PMC6438038

[5] A. Mari , F. A. Baker , R. Pellicano , and T. Khoury , “Diagnosis and Management of Achalasia: Updates of the Last Two Years,” Journal of Clinical Medicine 10, no. 16 (2021): 3607, 10.3390/JCM10163607. 34441901 PMC8397142

[6] R. Yadlapati , P. J. Kahrilas , M. R. Fox , et al., “Esophageal Motility Disorders on High Resolution Manometry: Chicago Classification Version 4.0©,” Neurogastroenterology and Motility 33, no. 1 (2021): e14058, 10.1111/NMO.14058. 33373111 PMC8034247

[7] E. Savarino , M. Di Pietro , A. J. Bredenoord , et al., “Use of the Functional Lumen Imaging Probe in Clinical Esophagology,” American Journal of Gastroenterology 115, no. 11 (2020): 1786–1796, 10.14309/AJG.0000000000000773. 33156096 PMC9380028

[8] P. Visaggi , M. Ghisa , E. Vespa , et al., “Optimal Assessment, Treatment, and Monitoring of Adults With Eosinophilic Esophagitis: Strategies to Improve Outcomes,” ImmunoTargets and Therapy 13 (2024): 367–383, 10.2147/ITT.S276869. 39071859 PMC11283784

[9] G. B. Nigam , D. H. Vasant , and A. Dhar , “Curriculum Review: Investigation and Management of Dysphagia,” Frontline Gastroenterology 13, no. 3 (2021): 254–261, 10.1136/FLGASTRO-2021-101917. 35493628 PMC8996094

[10] O. Dewidar , T. Lotfi , M. W. Langendam , et al., “Good or Best Practice Statements: Proposal for the Operationalisation and Implementation of GRADE Guidance,” BMJ Evidence‐Based Medicine 28, no. 3 (2023): 189–196, 10.1136/BMJEBM-2022-111962. PMC10313969 35428694

[11] E. B. McCarty and T. N. Chao , “Dysphagia and Swallowing Disorders,” Medical Clinics of North America 105, no. 5 (2021): 939–954, 10.1016/J.MCNA.2021.05.013. 34391544

[12] J. M. Wilkinson , D. C. Codipilly , and R. P. Wilfahrt , “Dysphagia: Evaluation and Collaborative Management,” American Family Physician 103 (2021): 97–106. 33448766

[13] ICD‐10 Version:2019, accessed October 23, 2024, https://icd.who.int/browse10/2019/en.

[14] J. R. Malagelada , F. Bazzoli , G. Boeckxstaens , et al., “World Gastroenterology Organisation Global Guidelines: Dysphagia–Global Guidelines and Cascades Update September 2014,” Journal of Clinical Gastroenterology 49, no. 5 (2015): 370–378, 10.1097/MCG.0000000000000307. 25853874

[15] R. Speyer , R. Cordier , D. Farneti , et al., “White Paper by the European Society for Swallowing Disorders: Screening and Non‐Instrumental Assessment for Dysphagia in Adults,” Dysphagia 37, no. 2 (2022): 333–349, 10.1007/S00455-021-10283-7. 33787994 PMC8009935

[16] H. Shaheen and H. Adeel , “Oropharyngeal Dysphagia,” in Dysphagia: A Clinical Guide (2024), 1–39, 10.1016/B978-0-443-19063-6.00002-6.

[17] A. H. Nielsen , S. J. Eskildsen , J. Danielsen , et al., “Defining Dysphagia – A Modified Multi‐Professional Danish Delphi Study,” Scandinavian Journal of Gastroenterology 58, no. 6 (2023): 583–588, 10.1080/00365521.2022.2151850. 36476215

[18] R. Speyer , R. Cordier , D. Denman , et al., “Development of Two Patient Self‐Reported Measures on Functional Health Status (FOD) and Health‐Related Quality of Life (QOD) in Adults With Oropharyngeal Dysphagia Using the Delphi Technique,” Journal of Clinical Medicine 11, no. 19 (2022): 5920, 10.3390/JCM11195920. 36233787 PMC9572600

[19] I. J. Cook and P. J. Kahrilas , “AGA Technical Review on Management of Oropharyngeal Dysphagia,” Gastroenterology 116, no. 2 (1999): 455–478, 10.1016/S0016-5085(99)70144-7. 9922328

[20] P. Clavé , R. Terré , M. de Kraa , and M. Serra , “Approaching Oropharyngeal Dysphagia,” Revista Española de Enfermedades Digestivas 96, no. 2 (2004): 119–131, 10.4321/s1130-01082004000200005. 15255021

[21] S. A. Riera , S. Marin , M. Serra‐Prat , et al., “A Systematic and a Scoping Review on the Psychometrics and Clinical Utility of the Volume‐Viscosity Swallow Test (V‐VST) in the Clinical Screening and Assessment of Oropharyngeal Dysphagia,” Foods 10, no. 8 (2021): 1900, 10.3390/FOODS10081900. 34441677 PMC8391460

[22] K. A. Hutcheson , M. P. Barrow , D. A. Barringer , et al., “Dynamic Imaging Grade of Swallowing Toxicity (DIGEST): Scale Development and Validation,” Cancer 123, no. 1 (2017): 62–70, 10.1002/CNCR.30283. 27564246 PMC5161634

[23] P. C. Belafsky , D. A. Mouadeb , C. J. Rees , et al., “Validity and Reliability of the Eating Assessment Tool (EAT‐10),” Annals of Otology, Rhinology & Laryngology 117, no. 12 (2008): 919–924, 10.1177/000348940811701210. 19140539

[24] K. Matsuo and J. B. Palmer , “Anatomy and Physiology of Feeding and Swallowing: Normal and Abnormal,” Physical Medicine and Rehabilitation Clinics of North America 19, no. 4 (2008): 691–707, 10.1016/J.PMR.2008.06.001. 18940636 PMC2597750

[25] M. N. Keles , H. I. Ertoy Karagol , A. S. Serel , et al., “Oropharyngeal Dysphagia in Children With Eosinophilic Esophagitis,” Dysphagia 38, no. 1 (2023): 474–482, 10.1007/S00455-022-10489-3/TABLES/4. 35781555

[26] B. T. Johnston , “Oesophageal Dysphagia: A Stepwise Approach to Diagnosis and Management,” Lancet Gastroenterology & Hepatology 2, no. 8 (2017): 604–609, 10.1016/S2468-1253(17)30001-8. 28691686

[27] T. Wilkins , R. A. Gillies , A. M. Thomas , and P. J. Wagner , “The Prevalence of Dysphagia in Primary Care Patients: A HamesNet Research Network Study,” Journal of the American Board of Family Medicine 20, no. 2 (2007): 144–150, 10.3122/JABFM.2007.02.060045. 17341750

[28] G. D. Eslick and N. J. Talley , “Dysphagia: Epidemiology, Risk Factors and Impact on Quality of Life – A Population‐Based Study,” Alimentary Pharmacology & Therapeutics 27, no. 10 (2008): 971–979, 10.1111/J.1365-2036.2008.03664.X. 18315591

[29] J. C. Chiocca , J. A. Olmos , G. B. Salis , L. O. Soifer , R. Higa , and M. Marcolongo , “Prevalence, Clinical Spectrum and Atypical Symptoms of Gastro‐Oesophageal Reflux in Argentina: A Nationwide Population‐Based Study,” Alimentary Pharmacology & Therapeutics 22, no. 4 (2005): 331–342, 10.1111/J.1365-2036.2005.02565.X. 16098000

[30] J. H. Wang , J. Y. Lou , L. Dong , et al., “Epidemiology of Gastroesophageal Reflux Disease: A General Population‐Based Study in Xi’an of Northwest China,” World Journal of Gastroenterology 10 (2004): 1647–1651, 10.3748/WJG.V10.I11.1647. 15162542 PMC4572771

[31] W. M. Wong , K. C. Lai , K. F. Lam , et al., “Prevalence, Clinical Spectrum and Health Care Utilization of Gastro‐Oesophageal Reflux Disease in a Chinese Population: A Population‐Based Study,” Alimentary Pharmacology & Therapeutics 18, no. 6 (2003): 595–604, 10.1046/J.1365-2036.2003.01737.X. 12969086

[32] A. D. Sperber , S. I. Bangdiwala , D. A. Drossman , et al., “Worldwide Prevalence and Burden of Functional Gastrointestinal Disorders, Results of Rome Foundation Global Study,” Gastroenterology 160, no. 1 (2021): 99–114.e3, 10.1053/J.GASTRO.2020.04.014. 32294476

[33] F. Rajati , N. Ahmadi , Z. A. S. Naghibzadeh , and M. Kazeminia , “The Global Prevalence of Oropharyngeal Dysphagia in Different Populations: A Systematic Review and Meta‐Analysis,” Journal of Translational Medicine 20 (2022): 1–15, 10.1186/S12967-022-03380-0/TABLES/4. 35410274 PMC9003990

[34] K. J. Banda , H. Chu , R. Chen , et al., “Prevalence of Oropharyngeal Dysphagia and Risk of Pneumonia, Malnutrition, and Mortality in Adults Aged 60 Years and Older: A Meta‐Analysis,” Gerontology 68, no. 8 (2022): 841–853, 10.1159/000520326. 34903688

[35] O. Ortega , A. Martín , and P. Clavé , “Diagnosis and Management of Oropharyngeal Dysphagia Among Older Persons, State of the Art,” Journal of the American Medical Directors Association 18 (2017): 576–582, 10.1016/J.JAMDA.2017.02.015. 28412164

[36] K. J. Banda , H. Chu , X. L. Kang , et al., “Prevalence of Dysphagia and Risk of Pneumonia and Mortality in Acute Stroke Patients: A Meta‐Analysis,” BMC Geriatrics 22, no. 1 (2022): 420, 10.1186/S12877-022-02960-5. 35562660 PMC9103417

[37] S. Gong , Y. Gao , J. Liu , et al., “The Prevalence and Associated Factors of Dysphagia in Parkinson’s Disease: A Systematic Review and Meta‐Analysis,” Frontiers in Neurology 13 (2022), 10.3389/FNEUR.2022.1000527/FULL. PMC9582284 36277913

[38] C. Takizawa , E. Gemmell , J. Kenworthy , and R. Speyer , “A Systematic Review of the Prevalence of Oropharyngeal Dysphagia in Stroke, Parkinson’s Disease, Alzheimer’s Disease, Head Injury, and Pneumonia,” Dysphagia 31, no. 3 (2016): 434–441, 10.1007/S00455-016-9695-9/FIGURES/1. 26970760

[39] B. Labeit , M. Pawlitzki , T. Ruck , et al., “The Impact of Dysphagia in Myositis: A Systematic Review and Meta‐Analysis,” Journal of Clinical Medicine 9, no. 7 (2020): 1–22, 10.3390/JCM9072150. PMC7408750 32650400

[40] S. Marin , M. Serra‐Prat , O. Ortega , and P. Clavé , “Healthcare‐Related Cost of Oropharyngeal Dysphagia and Its Complications Pneumonia and Malnutrition After Stroke: A Systematic Review,” BMJ Open 10, no. 8 (2020): e031629, 10.1136/BMJOPEN-2019-031629. PMC7418658 32784251

[41] D. A. Patel , S. Krishnaswami , E. Steger , et al., “Economic and Survival Burden of Dysphagia Among Inpatients in the United States,” Diseases of the Esophagus 31, no. 1 (2018): dox131, 10.1093/DOTE/DOX131. 29155982 PMC6454833

[42] S. Attrill , S. White , J. Murray , S. Hammond , and S. Doeltgen , “Impact of Oropharyngeal Dysphagia on Healthcare Cost and Length of Stay in Hospital: A Systematic Review,” BMC Health Services Research 18, no. 1 (2018): 594, 10.1186/S12913-018-3376-3. 30068326 PMC6090960

[43] A. W. Wojner and A. V. Alexandrov , “Predictors of Tube Feeding in Acute Stroke Patients With Dysphagia,” AACN Clinical Issues 11, no. 4 (2000): 531–540, 10.1097/00044067-200011000-00006. 11288417

[44] H. S. Bonilha , A. N. Simpson , C. Ellis , P. Mauldin , B. Martin‐Harris , and K. Simpson , “The One‐Year Attributable Cost of Post‐Stroke Dysphagia,” Dysphagia 29, no. 5 (2014): 545–552, 10.1007/S00455-014-9543-8. 24948438 PMC4179977

[45] L. Rofes , D. Muriana , E. Palomeras , et al., “Prevalence, Risk Factors and Complications of Oropharyngeal Dysphagia in Stroke Patients: A Cohort Study,” Neurogastroenterology and Motility 30, no. 8 (2018): e13338, 10.1111/NMO.13338. 29573064

[46] S. Marin , M. Serra‐Prat , O. Ortega , et al., “Healthcare Costs of Post‐Stroke Oropharyngeal Dysphagia and Its Complications: Malnutrition and Respiratory Infections,” European Journal of Neurology 28 (2021): 3670–3681, 10.1111/ENE.14998. 34176195

[47] B. Labeit , A. Kremer , P. Muhle , et al., “Costs of Post‐Stroke Dysphagia During Acute Hospitalization From a Health‐Insurance Perspective,” European Stroke Journal 8, no. 1 (2023): 361–369, 10.1177/23969873221147740. 37021194 PMC10069210

[48] S. Marin , O. Ortega , M. Serra‐Prat , E. Valls , L. Pérez‐Cordón , and P. Clavé , “Economic Evaluation of Clinical, Nutritional and Rehabilitation Interventions on Oropharyngeal Dysphagia After Stroke: A Systematic Review,” Nutrients 15, no. 7 (2023): 1714, 10.3390/NU15071714/S1. 37049553 PMC10097035

[49] S. Westmark , D. Melgaard , L. O. Rethmeier , and L. H. Ehlers , “The Cost of Dysphagia in Geriatric Patients,” ClinicoEconomics and Outcomes Research 10 (2018): 321–326, 10.2147/CEOR.S165713. 29922079 PMC5995296

[50] D. G. Di Luca , E. W. McArthur , A. Willis , R. Martino , and C. Marras , “Clinical and Economic Outcomes Associated With Dysphagia in Hospitalized Patients With Parkinson’s Disease,” Journal of Parkinson's Disease 11, no. 4 (2021): 1965–1971, 10.3233/JPD-212798. 34366378

[51] K. W. Altman , G. P. Yu , and S. D. Schaefer , “Consequence of Dysphagia in the Hospitalized Patient: Impact on Prognosis and Hospital Resources,” Archives of Otolaryngology ‐ Head and Neck Surgery 136, no. 8 (2010): 784–789, 10.1001/ARCHOTO.2010.129. 20713754

[52] O. Ekberg , S. Hamdy , V. Woisard , A. Wuttge‐Hannig , and P. Ortega , “Social and Psychological Burden of Dysphagia: Its Impact on Diagnosis and Treatment,” Dysphagia 17, no. 2 (2002): 139–146, 10.1007/S00455-001-0113-5. 11956839

[53] A. Mari and R. Sweis , “Assessment and Management of Dysphagia and Achalasia,” Clinical Medicine 21, no. 2 (2021): 119–123, 10.7861/CLINMED.2021-0069. 33762370 PMC8002782

[54] M. Panebianco , R. Marchese‐Ragona , S. Masiero , and D. A. Restivo , “Dysphagia in Neurological Diseases: A Literature Review,” Neurological Sciences 41, no. 11 (2020): 3067–3073, 10.1007/S10072-020-04495-2. 32506360 PMC7567719

[55] N. Rommel and S. Hamdy , “Oropharyngeal Dysphagia: Manifestations and Diagnosis,” Nature Reviews Gastroenterology & Hepatology 13, no. 1 (2016): 49–59, 10.1038/NRGASTRO.2015.199. 26627547

[56] A. Pasta , C. Facchini , F. Calabrese , et al., “Esophageal Motor Disorders Across Ages: A Retrospective Multicentric Analysis,” Journal of the American Geriatrics Society 72, no. 9 (2024): 2782–2791, 10.1111/JGS.19068. 38975863

[57] M. R. Shaik , N. A. Shaik , and J. Mikdashi , “Autoimmune Dysphagia Related to Rheumatologic Disorders: A Focused Review on Diagnosis and Treatment,” Cureus 15 (2023): e41883, 10.7759/CUREUS.41883. 37581141 PMC10423619

[58] Y. Marten Canavesio , A. Pasta , F. Calabrese , et al., “Association Between Esophageal Motor Disorders and Pulmonary Involvement in Patients Affected by Systemic Sclerosis: A Retrospective Study,” Rheumatology International 44, no. 12 (2023): 2905–2910, 10.1007/S00296-023-05399-Y. 37542603

[59] S. Negrini , G. Emmi , M. Greco , et al., “Sjögren’s Syndrome: A Systemic Autoimmune Disease,” Clinical and Experimental Medicine 22, no. 1 (2022): 9–25, 10.1007/S10238-021-00728-6. 34100160 PMC8863725

[60] A. Abraham , R. Hajar , R. Virdi , J. Singh , and P. Mustacchia , “Esophageal Sarcoidosis: A Review of Cases and an Update,” ISRN Gastroenterology 2013 (2013): 1–9, 10.1155/2013/836203. PMC3603204 23533794

[61] B. Krishnan , S. Babu , J. Walker , et al., “Gastrointestinal Complications of Diabetes Mellitus,” World Journal of Diabetes 4 (2013): 51, 10.4239/WJD.V4.I3.51. 23772273 PMC3680624

[62] R. Asayama , K. Tanaka‐Nishikubo , M. Okada , et al., “Dysphagia in Patients With Severe COVID‐19: A Retrospective Study,” Scientific Reports 14, no. 1 (2024): 6829, 10.1038/S41598-024-57508-X. 38514734 PMC10957916

[63] P. Hoversten , A. K. Kamboj , and D. A. Katzka , “Infections of the Esophagus: An Update on Risk Factors, Diagnosis, and Management,” Diseases of the Esophagus 31 (2018): doy094, 10.1093/DOTE/DOY094. 30295751

[64] S. Jadcherla , “Dysphagia in the High‐Risk Infant: Potential Factors and Mechanisms,” American Journal of Clinical Nutrition 103, no. 2 (2016): 622S–628S, 10.3945/AJCN.115.110106. 26791178 PMC4733255

[65] L. R. Carucci and M. Ann Turner , “Dysphagia Revisited: Common and Unusual Causes,” RadioGraphics 35, no. 1 (2015): 105–122, 10.1148/RG.351130150. 25590391

[66] J. E. Raber‐Durlacher , M. T. Brennan , I. M. Verdonck‐De Leeuw , et al., “Swallowing Dysfunction in Cancer Patients,” Supportive Care in Cancer 20, no. 3 (2012): 433–443, 10.1007/S00520-011-1342-2. 22205548 PMC3271214

[67] R. Yadlapati , J. E. Pandolfino , M. R. Fox , A. J. Bredenoord , and P. J. Kahrilas , “What Is New in Chicago Classification Version 4.0?,” Neurogastroenterology and Motility 33, no. 1 (2021), 10.1111/NMO.14053. PMC8098672 33340190

[68] A. Abdelghani , A. Ibrahim , E. S. El‐Sayed , M. El Sherbiny , and A. Al‐Badry , “Esophageal Motility Disorders in Symptomatic Patients and Its Relation to Age,” BMC Gastroenterology 23, no. 1 (2023): 69, 10.1186/S12876-023-02709-3. 36906548 PMC10007782

[69] M. R. Fox , J. E. Pandolfino , R. Sweis , et al., “Inter‐Observer Agreement for Diagnostic Classification of Esophageal Motility Disorders Defined in High‐Resolution Manometry,” Diseases of the Esophagus 28, no. 8 (2015): 711–719, 10.1111/DOTE.12278. 25185507

[70] D. L. Cohen and H. Shirin , “Technical Success in Performing Esophageal High‐Resolution Manometry: A Review of Competency Recommendations, Predictors of Failure, and Alternative Techniques,” Diseases of the Esophagus 36, no. 8 (2023): doad013, 10.1093/DOTE/DOAD013. 36912065

[71] R. Katsumata , N. Manabe , H. Sakae , et al., “Clinical Characteristics and Manometric Findings of Esophageal Achalasia—A Systematic Review Regarding Differences Among Three Subtypes,” Journal of Smooth Muscle Research 59 (2023): 14–27, 10.1540/JSMR.59.14. 36948611 PMC10036217

[72] S. Roman , L. Huot , F. Zerbib , et al., “High‐Resolution Manometry Improves the Diagnosis of Esophageal Motility Disorders in Patients With Dysphagia: A Randomized Multicenter Study,” American Journal of Gastroenterology 111, no. 3 (2016): 372–380, 10.1038/AJG.2016.1. 26832656

[73] S. L. Popa , T. Surdea‐Blaga , D. L. Dumitrascu , et al., “Automatic Diagnosis of High‐Resolution Esophageal Manometry Using Artificial Intelligence,” Journal of Gastrointestinal & Liver Diseases 31, no. 4 (2022): 383–389, 10.15403/JGLD-4525. 36535043

[74] J. E. Pandolfino , S. Roman , D. Carlson , et al., “Distal Esophageal Spasm in High‐Resolution Esophageal Pressure Topography: Defining Clinical Phenotypes,” Gastroenterology 141, no. 2 (2011): 469–475, 10.1053/J.GASTRO.2011.04.058. 21679709 PMC3626105

[75] H. U. De Schepper , F. A. M. Ponds , J. M. Oors , A. J. P. M. Smout , and A. J. Bredenoord , “Distal Esophageal Spasm and the Chicago Classification: Is Timing Everything?,” Neurogastroenterology and Motility 28, no. 2 (2016): 260–265, 10.1111/NMO.12721. 26553751

[76] S. Roman , G. Hebbard , K. W. Jung , et al., “Chicago Classification Update (v4.0): Technical Review on Diagnostic Criteria for Distal Esophageal Spasm,” Neurogastroenterology and Motility 33, no. 5 (2021): e14119, 10.1111/NMO.14119. 33666299

[77] D. Ang , B. Misselwitz , M. Hollenstein , et al., “Diagnostic Yield of High‐Resolution Manometry With a Solid Test Meal for Clinically Relevant, Symptomatic Oesophageal Motility Disorders: Serial Diagnostic Study,” Lancet Gastroenterology & Hepatology 2, no. 9 (2017): 654–661, 10.1016/S2468-1253(17)30148-6. 28684262

[78] M. R. Fox , R. Sweis , R. Yadlapati , et al., “Chicago Classification Version 4.0© Technical Review: Update on Standard High‐Resolution Manometry Protocol for the Assessment of Esophageal Motility HHS Public Access,” Neurogastroenterology and Motility 33, no. 4 (2021): 14120, 10.1111/nmo.14120. PMC8268048 33729668

[79] D. Sifrim , J. Janssens , and G. Vantrappen , “Failing Deglutitive Inhibition in Primary Esophageal Disorders,” Gastroenterology 108 (1994): 875882. 10.1016/0016-5085(94)90745-5 8143993

[80] A. Bogte , A. J. Bredenoord , J. Oors , P. D. Siersema , and A. J. P. M. Smout , “Sensation of Stasis Is Poorly Correlated With Impaired Esophageal Bolus Transport,” Neurogastroenterology and Motility 26, no. 4 (2014): 538–545, 10.1111/NMO.12298. 24372856

[81] D. Pohl , J. Ciolino , J. Roberts , et al., “Functional Aspects of Distal Oesophageal Spasm: The Role of Onset Velocity and Contraction Amplitude on Bolus Transit,” Digestive and Liver Disease 44, no. 7 (2012): 569–575, 10.1016/j.dld.2012.02.003. 22475443 PMC3477870

[82] R. Tutuian , I. Mainie , A. Agrawal , R. M. Gideon , P. O. Katz , and D. O. Castell , “Symptom and Function Heterogenicity Among Patients With Distal Esophageal Spasm: Studies Using Combined Impedance‐Manometry,” American Journal of Gastroenterology 101, no. 3 (2006): 464–469, 10.1111/J.1572-0241.2006.00408.X. 16542281

[83] E. Savarino and A. J. P. M. Smout , “The Hypercontractile Esophagus: Still a Tough Nut to Crack,” Neurogastroenterology and Motility 32, no. 11 (2020), 10.1111/NMO.14010. PMC7685127 33043556

[84] S. Roman , J. E. Pandolfino , J. Chen , L. Boris , D. Luger , and P. J. Kahrilas , “Phenotypes and Clinical Context of Hypercontractility in High Resolution Esophageal Pressure Topography (EPT),” American Journal of Gastroenterology 107, no. 1 (2012): 37–45, 10.1038/AJG.2011.313. 21931377 PMC3641840

[85] T. V. K. Herregods , A. J. P. M. Smout , J. L. S. Ooi , D. Sifrim , and A. J. Bredenoord , “Jackhammer Esophagus: Observations on a European Cohort,” Neurogastroenterology and Motility 29, no. 4 (2017): e12975, 10.1111/NMO.12975. 27753176

[86] G. Wahba and M. Bouin , “Jackhammer Esophagus: A Meta‐Analysis of Patient Demographics, Disease Presentation, High‐Resolution Manometry Data, and Treatment Outcomes,” Neurogastroenterology and Motility 32, no. 11 (2020): e13870, 10.1111/NMO.13870. 32406556

[87] N. de Bortoli , P. C. Gyawali , S. Roman , et al., “Hypercontractile Esophagus From Pathophysiology to Management: Proceedings of the Pisa Symposium,” American Journal of Gastroenterology 116, no. 2 (2021): 263–273, 10.14309/AJG.0000000000001061. 33273259

[88] M. Carmel , D. L. Cohen , B. Hijazi , et al., “Chicago Classification Version 4.0 Improves Stratification of Ineffective Esophageal Motility Patients Into Clinically Meaningful Subtypes: A Two‐Center International Study,” Dysphagia 39, no. 3 (2024): 444–451, 10.1007/S00455-023-10628-4/TABLES/5. 37934251

[89] C. P. Gyawali , D. Sifrim , D. A. Carlson , et al., “Ineffective Esophageal Motility: Concepts, Future Directions, and Conclusions From the Stanford 2018 Symposium,” Neurogastroenterology and Motility 31, no. 9 (2019): e13584, 10.1111/NMO.13584. 30974032 PMC9380027

[90] P. Pouderoux , G. Shi , R. P. Tatum , and P. J. Kahrilas , “Esophageal Solid Bolus Transit: Studies Using Concurrent Videofluoroscopy and Manometry,” American Journal of Gastroenterology 94, no. 6 (1999): 1457–1463, 10.1111/J.1572-0241.1999.01126.X. 10364007

[91] B. D. Rogers , D. Cisternas , A. Rengarajan , et al., “Breaks in Peristaltic Integrity Predict Abnormal Esophageal Bolus Clearance Better Than Contraction Vigor or Residual Pressure at the Esophagogastric Junction,” Neurogastroenterology and Motility 34, no. 2 (2022): e14141, 10.1111/NMO.14141. 33772977

[92] S. K. Ghosh , J. E. Pandolfino , M. A. Kwiatek , and P. J. Kahrilas , “Oesophageal Peristaltic Transition Zone Defects: Real But Few and Far Between,” Neurogastroenterology and Motility 20, no. 12 (2008): 1283–1290, 10.1111/J.1365-2982.2008.01169.X. 18662328 PMC2886597

[93] S. Roman , Z. Lin , M. A. Kwiatek , J. E. Pandolfino , and P. J. Kahrilas , “Weak Peristalsis in Esophageal Pressure Topography: Classification and Association With Dysphagia,” American Journal of Gastroenterology 106, no. 2 (2011): 349–356, 10.1038/AJG.2010.384. 20924368 PMC3035759

[94] J. F. Wu , I. J. Tsai , T. W. Tong , Y. Lin , C. Yang , and P. Tseng , “Pressure–Impedance Analysis: Assist the Diagnosis and Classification of Ineffective Esophageal Motility Disorder,” Journal of Gastroenterology and Hepatology 35, no. 8 (2020): 1317–1324, 10.1111/JGH.14981. 31927770

[95] P. Chugh , T. Collazo , B. Dworkin , and D. Jodorkovsky , “Ineffective Esophageal Motility Is Associated With Impaired Bolus Clearance But Does Not Correlate With Severity of Dysphagia,” Digestive Diseases and Sciences 64, no. 3 (2019): 811–814, 10.1007/S10620-018-5384-X/METRICS. 30535781

[96] M. Sallette , J. Lenz , F. Mion , and S. Roman , “From Chicago Classification v3.0 to v4.0: Diagnostic Changes and Clinical Implications,” Neurogastroenterology and Motility 35, no. 1 (2023): e14467, 10.1111/NMO.14467. 36314395 PMC10078267

[97] A. Sawada , M. Zhang , A. Ustaoglu , et al., “Superficial Oesophageal Mucosal Innervation May Contribute to Severity of Symptoms in Oesophageal Motility Disorders,” Alimentary Pharmacology & Therapeutics 59, no. 1 (2024): 100–112, 10.1111/APT.17773. 37845817

[98] Q. Aziz , R. Fass , C. P. Gyawali , H. Miwa , J. E. Pandolfino , and F. Zerbib , “Esophageal Disorders,” Gastroenterology 150, no. 6 (2016): 1368–1379, 10.1053/j.gastro.2016.02.012. 27144625

[99] A. Bogte , A. J. Bredenoord , J. Oors , P. D. Siersema , and A. J. P. M. Smout , “Relationship Between Esophageal Contraction Patterns and Clearance of Swallowed Liquid and Solid Boluses in Healthy Controls and Patients With Dysphagia,” Neurogastroenterology and Motility 24, no. 8 (2012): e364–e372, 10.1111/J.1365-2982.2012.01949.X. 22672410

[100] A. D. Farmer , J. K. Ruffle , and Q. Aziz , “The Role of Esophageal Hypersensitivity in Functional Esophageal Disorders,” Journal of Clinical Gastroenterology 51, no. 2 (2017): 91–99, 10.1097/MCG.0000000000000757. 28005634

[101] A. Lazarescu , G. Karamanolis , L. Aprile , R. B. De Oliveira , R. Dantas , and D. Sifrim , “Perception of Dysphagia: Lack of Correlation With Objective Measurements of Esophageal Function,” Neurogastroenterology and Motility 22, no. 12 (2010): 1292‐e337, 10.1111/J.1365-2982.2010.01578.X. 20718946

[102] D. A. Drossman and W. L. Hasler , “Rome IV – Functional GI Disorders: Disorders of Gut‐Brain Interaction,” Gastroenterology 150, no. 6 (2016): 1257–1261, 10.1053/j.gastro.2016.03.035. 27147121

[103] P. Woodland , S. Gabieta‐Sonmez , J. Arguero , et al., “200 mL Rapid Drink Challenge During High‐Resolution Manometry Best Predicts Objective Esophagogastric Junction Obstruction and Correlates With Symptom Severity,” Journal of Neurogastroenterology and Motility 24, no. 3 (2018): 410–414, 10.5056/JNM18038. 29969859 PMC6034657

[104] K. L. Lynch , J. Chen , A. Jain , et al., “Esophagogastric Junction Outflow Obstruction: A Diagnosis in Evolution,” Gastroenterology & Hepatology 20 (2024): 108. 38414912 PMC10895913

[105] A. S. Jain , C. Allamneni , M. Kline , et al., “Relationship Between Dysphagia, Lower Esophageal Sphincter Relaxation, and Esophagogastric Junction Distensibility,” Neurogastroenterology and Motility 34, no. 8 (2022): e14319, 10.1111/NMO.14319. 35060256

[106] J. Y. Choi , K. W. Jung , J. E. Pandolfino , et al., “Dysphagia Associated With Esophageal Wall Thickening in Patients With Nonspecific High‐Resolution Manometry Findings: Understanding Motility Beyond the Chicago Classification Version 4.0,” Neurogastroenterology and Motility 36, no. 4 (2024): e14736, 10.1111/NMO.14736. 38225864

[107] Y. Xiao , P. J. Kahrilas , F. Nicodème , et al., “Lack of Correlation Between HRM Metrics and Symptoms During the Manometric Protocol,” American Journal of Gastroenterology 109 (2014): 521, 10.1038/AJG.2014.13. 24513804 PMC4120962

[108] R. K. Mittal and A. Zifan , “Why So Many Patients With Dysphagia Have Normal Esophageal Function Testing,” Gastro Hep Advances 3, no. 1 (2024): 109–121, 10.1016/j.gastha.2023.08.021. 38420259 PMC10899865

[109] S. Sanagapalli , J. McGuire , R. W. Leong , et al., “The Clinical Relevance of Manometric Esophagogastric Junction Outflow Obstruction Can Be Determined Using Rapid Drink Challenge and Solid Swallows,” American Journal of Gastroenterology 116, no. 2 (2021): 280–288, 10.14309/AJG.0000000000000988. 33136563

[110] D. A. Patel , R. Yadlapati , and M. F. Vaezi , “Esophageal Motility Disorders: Current Approach to Diagnostics and Therapeutics,” Gastroenterology 162, no. 6 (2022): 1617–1634, 10.1053/J.GASTRO.2021.12.289. 35227779 PMC9405585

[111] D. A. Carlson , W. Kou , Z. Lin , et al., “Normal Values of Esophageal Distensibility and Distension‐Induced Contractility Measured by Functional Luminal Imaging Probe Panometry,” Clinical Gastroenterology and Hepatology 17, no. 4 (2019): 674–681.e1, 10.1016/J.CGH.2018.07.042. 30081222 PMC6360138

[112] S. K. Ghosh , J. E. Pandolfino , J. Rice , J. O. Clarke , M. Kwiatek , and P. J. Kahrilas , “Impaired Deglutitive EGJ Relaxation in Clinical Esophageal Manometry: A Quantitative Analysis of 400 Patients and 75 Controls,” American Journal of Physiology ‐ Gastrointestinal and Liver Physiology 293, no. 4 (2007): G878–G885, 10.1152/AJPGI.00252.2007. 17690172

[113] R. A. B. Oude Nijhuis , G. Zaninotto , S. Roman , et al., “European Guideline on Achalasia – UEG and ESNM Recommendations,” United European Gastroenterology Journal 8, no. 1 (2020): 13–33, 10.1177/2050640620903213. 32213062 PMC7005998

[114] J. E. Pandolfino and A. J. Gawron , “Achalasia: A Systematic Review,” JAMA 313, no. 18 (2015): 1841–1852, 10.1001/JAMA.2015.2996. 25965233

[115] J. E. Pandolfino , M. A. Kwiatek , T. Nealis , W. Bulsiewicz , J. Post , and P. J. Kahrilas , “Achalasia: A New Clinically Relevant Classification by High‐Resolution Manometry,” Gastroenterology 135, no. 5 (2008): 1526–1533, 10.1053/J.GASTRO.2008.07.022. 18722376 PMC2894987

[116] P. J. Kahrilas and G. Boeckxstaens , “The Spectrum of Achalasia: Lessons From Studies of Pathophysiology and High‐Resolution Manometry,” Gastroenterology 145, no. 5 (2013): 954–965, 10.1053/J.GASTRO.2013.08.038. 23973923 PMC3835179

[117] D. A. Carlson , J. M. Schauer , W. Kou , P. J. Kahrilas , and J. E. Pandolfino , “Functional Lumen Imaging Probe Panometry Helps Identify Clinically Relevant Esophagogastric Junction Outflow Obstruction Per Chicago Classification v4.0,” American Journal of Gastroenterology 118, no. 1 (2023): 77–86, 10.14309/AJG.0000000000001980. 36002925 PMC9822847

[118] L. K. Besanko , C. M. Burgstad , R. Mountifield , et al., “Lower Esophageal Sphincter Relaxation Is Impaired in Older Patients With Dysphagia,” World Journal of Gastroenterology: WJG 17 (2011): 1326, 10.3748/WJG.V17.I10.1326. 21455332 PMC3068268

[119] R. E. Kraichely , A. S. Arora , and J. A. Murray , “Opiate‐Induced Oesophageal Dysmotility,” Alimentary Pharmacology & Therapeutics 31, no. 5 (2010): 601–606, 10.1111/J.1365-2036.2009.04212.X. 20003176 PMC3092396

[120] W. Schima , E. Schober , G. Stacher , et al., “Association of Midoesophageal Diverticula With Oesophageal Motor Disorders. Videofluoroscopy and Manometry,” Acta Radiologica 38, no. 1 (1997): 108–114, 10.1080/02841859709171252. 9059412

[121] D. L. Cohen , A. Bermont , V. Richter , E. Avivi , A. Mari , and H. Shirin , “Technical Success in Performing Esophageal High‐Resolution Manometry in Patients With an Epiphrenic Diverticulum,” Dysphagia 39, no. 2 (2024): 282–288, 10.1007/S00455-023-10610-0. 37542551

[122] A. A. Mohamed , X. L. Lu , and F. A. Mounmin , “Diagnosis and Treatment of Esophageal Candidiasis: Current Updates,” Chinese Journal of Gastroenterology and Hepatology 2019 (2019): 1–6, 10.1155/2019/3585136. PMC6854261 31772927

[123] Z. Shen , Y. Hou , A. Huerman , and A. Ma , “Patients With Dysphagia: How to Supply Nutrition Through Non‐Tube Feeding,” Frontiers in Nutrition 9 (2022), 10.3389/FNUT.2022.1060630. PMC9757495 36532550

[124] A. Martin–Martinez , O. Ortega , P. Viñas , et al., “COVID‐19 Is Associated With Oropharyngeal Dysphagia and Malnutrition in Hospitalized Patients During the Spring 2020 Wave of the Pandemic,” Clinical Nutrition 41, no. 12 (2022): 2996–3006, 10.1016/J.CLNU.2021.06.010. 34187698 PMC8205257

[125] M. Cabré , M. Serra‐Prat , L. Force , et al., “Oropharyngeal Dysphagia Is a Risk Factor for Readmission for Pneumonia in the Very Elderly Persons: Observational Prospective Study,” Journals of Gerontology Series A: Biomedical Sciences and Medical Sciences 69 (2014): 330–337, 10.1093/GERONA/GLT099. 23833199

[126] R. Wirth , R. Dziewas , A. M. Beck , et al., “Oropharyngeal Dysphagia in Older Persons – From Pathophysiology to Adequate Intervention: A Review and Summary of an International Expert Meeting,” Clinical Interventions in Aging 11 (2016): 189–208, 10.2147/CIA.S97481. 26966356 PMC4770066

[127] L. Rofes , V. Arreola , M. Romea , et al., “Pathophysiology of Oropharyngeal Dysphagia in the Frail Elderly,” Neurogastroenterology and Motility 22, no. 8 (2010): 851, 10.1111/J.1365-2982.2010.01521.X. 20529208

[128] R. Martino , N. Foley , S. Bhogal , N. Diamant , M. Speechley , and R. Teasell , “Dysphagia After Stroke: Incidence, Diagnosis, and Pulmonary Complications,” Stroke 36, no. 12 (2005): 2756–2763, 10.1161/01.STR.0000190056.76543.EB. 16269630

[129] A. S. Morgan and L. E. Mackay , “Causes and Complications Associated With Swallowing Disorders in Traumatic Brain Injury,” Journal of Head Trauma Rehabilitation 14, no. 5 (1999): 454–461, 10.1097/00001199-199910000-00006. 10653941

[130] A. Costa , A. Martin , V. Arreola , et al., “Assessment of Swallowing Disorders, Nutritional and Hydration Status, and Oral Hygiene in Students With Severe Neurological Disabilities Including Cerebral Palsy,” Nutrients 13, no. 7 (2021): 2413, 10.3390/NU13072413/S1. 34371923 PMC8308512

[131] I. Suttrup and T. Warnecke , “Dysphagia in Parkinson’s Disease,” Dysphagia 31, no. 1 (2016): 24–32, 10.1007/S00455-015-9671-9. 26590572

[132] K. A. Hutcheson , Z. Nurgalieva , H. Zhao , et al., “Two‐Year Prevalence of Dysphagia and Related Outcomes in Head and Neck Cancer Survivors: An Updated SEER‐Medicare Analysis,” Head & Neck 41, no. 2 (2019): 479–487, 10.1002/HED.25412. 30536748 PMC6355350

[133] H. E. Choi , G. Y. Jo , W. J. Kim , H. K. Do , J. K. Kwon , and S. H. Park , “Characteristics and Clinical Course of Dysphagia Caused by Anterior Cervical Osteophyte,” Annals of Rehabilitation Medicine 43, no. 1 (2019): 27–37, 10.5535/ARM.2019.43.1.27. 30852868 PMC6409658

[134] I. Cheng , K. Takahashi , A. Miller , and S. Hamdy , “Cerebral Control of Swallowing: An Update on Neurobehavioral Evidence,” Journal of the Neurological Sciences 442 (2022): 120434, 10.1016/J.JNS.2022.120434. 36170765

[135] N. Tomsen , O. Ortega , D. Alvarez‐Berdugo , L. Rofes , and P. Clavé , “A Comparative Study on the Effect of Acute Pharyngeal Stimulation With TRP Agonists on the Biomechanics and Neurophysiology of Swallow Response in Patients With Oropharyngeal Dysphagia,” International Journal of Molecular Sciences 23, no. 18 (2022): 10773, 10.3390/IJMS231810773. 36142680 PMC9506471

[136] C. Cabib , O. Ortega , N. Vilardell , L. Mundet , P. Clavé , and L. Rofes , “Chronic Post‐Stroke Oropharyngeal Dysphagia Is Associated With Impaired Cortical Activation to Pharyngeal Sensory Inputs,” European Journal of Neurology 24, no. 11 (2017): 1355–1362, 10.1111/ENE.13392. 28872738

[137] S. Suntrup , I. Teismann , J. Bejer , et al., “Evidence for Adaptive Cortical Changes in Swallowing in Parkinson’s Disease,” Brain 136, no. 3 (2013): 726–738, 10.1093/BRAIN/AWT004. 23412935

[138] N. Vilardell , L. Rofes , V. Arreola , et al., “Videofluoroscopic Assessment of the Pathophysiology of Chronic Poststroke Oropharyngeal Dysphagia,” Neurogastroenterology and Motility 29, no. 10 (2017): 1–8, 10.1111/NMO.13111. 28547922

[139] M. Serra‐Prat , M. Palomera , C. Gomez , et al., “Oropharyngeal Dysphagia as a Risk Factor for Malnutrition and Lower Respiratory Tract Infection in Independently Living Older Persons: A Population‐Based Prospective Study,” Age and Ageing 41, no. 3 (2012): 376–381, 10.1093/AGEING/AFS006. 22311895

[140] R. Mehanna and J. Jankovic , “Respiratory Problems in Neurologic Movement Disorders,” Parkinsonism & Related Disorders 16, no. 10 (2010): 628–638, 10.1016/J.PARKRELDIS.2010.07.004. 20674459

[141] P. E. Marik , “Aspiration Pneumonitis and Aspiration Pneumonia,” New England Journal of Medicine 344, no. 9 (2001): 665–671, 10.1056/NEJM200103013440908. 11228282

[142] L. A. Mandell and M. S. Niederman , “Aspiration Pneumonia,” New England Journal of Medicine 380, no. 7 (2019): 651–663, 10.1056/NEJMRA1714562. 30763196

[143] D. G. Smithard and Y. Yoshimatsu , “Pneumonia, Aspiration Pneumonia, or Frailty‐Associated Pneumonia?,” Geriatrics (Basel) 7, no. 5 (2022): 115, 10.3390/GERIATRICS7050115. 36286218 PMC9602119

[144] S. Carrión , M. Cabré , R. Monteis , et al., “Oropharyngeal Dysphagia Is a Prevalent Risk Factor for Malnutrition in a Cohort of Older Patients Admitted With an Acute Disease to a General Hospital,” Clinical Nutrition 34, no. 3 (2015): 436–442, 10.1016/j.clnu.2014.04.014. 24882372

[145] A. M. Namasivayam and C. M. Steele , “Malnutrition and Dysphagia in Long‐Term Care: A Systematic Review,” Journal of Nutrition in Gerontology and Geriatrics 34 (2015): 1–21, 10.1080/21551197.2014.1002656. 25803601

[146] N. C. Foley , R. E. Martin , K. L. Salter , and R. Teasell , “A Review of the Relationship Between Dysphagia and Malnutrition Following Stroke,” Journal of Rehabilitation Medicine 41, no. 9 (2009): 707–713, 10.2340/16501977-0415. 19774302

[147] P. Viñas , M. Bolivar‐Prados , N. Tomsen , et al., “The Hydration Status of Adult Patients With Oropharyngeal Dysphagia and the Effect of Thickened Fluid Therapy on Fluid Intake and Hydration: Results of Two Parallel Systematic and Scoping Reviews,” Nutrients 14, no. 12 (2022): 2497, 10.3390/NU14122497. 35745228 PMC9228104

[148] S. Bushuven , I. Niebel , J. Huber , and P. Diesener , “Emotional and Psychological Effects of Dysphagia: Validation of the Jugendwerk Dysphagia Emotion and Family Assessment (JDEFA),” Dysphagia 37, no. 2 (2022): 375–391, 10.1007/S00455-021-10289-1/TABLES/4. 33817751 PMC8019588

[149] G. D. Eslick , M. P. Jones , and N. J. Talley , “Non‐Cardiac Chest Pain: Prevalence, Risk Factors, Impact and Consulting–A Population‐Based Study,” Alimentary Pharmacology & Therapeutics 17, no. 9 (2003): 1115–1124, 10.1046/J.1365-2036.2003.01557.X. 12752348

[150] Y. Manor , M. Balas , N. Giladi , R. Mootanah , and J. T. Cohen , “Anxiety, Depression and Swallowing Disorders in Patients With Parkinson’s Disease,” Parkinsonism & Related Disorders 15 (2009): 453–456, 10.1016/j.parkreldis.2008.11.005. 19071054

[151] R. J. C. G. Verdonschot , L. W. J. Baijens , J. L. Serroyen , C. Leue , and B. Kremer , “Symptoms of Anxiety and Depression Assessed With the Hospital Anxiety and Depression Scale in Patients With Oropharyngeal Dysphagia,” Journal of Psychosomatic Research 75 (2013): 451–455, 10.1016/J.JPSYCHORES.2013.08.021. 24182634

[152] I. Bjelland , A. A. Dahl , T. T. Haug , and D. Neckelmann , “The Validity of the Hospital Anxiety and Depression Scale: An Updated Literature Review,” Journal of Psychosomatic Research 52, no. 2 (2002): 69–77, 10.1016/S0022-3999(01)00296-3. 11832252

[153] M. J. Schmulson and D. A. Drossman , “What Is New in Rome IV,” Journal of Neurogastroenterology and Motility 23, no. 2 (2017): 151–163, 10.5056/JNM16214. 28274109 PMC5383110

[154] B. T. Johnston , “Oesophageal Dysphagia: A Stepwise Approach to Diagnosis and Management,” Lancet Gastroenterology & Hepatology 2, no. 8 (2017): 604–609, 10.1016/S2468-1253(17)30001-8. 28691686

[155] L. W. C. Liu , C. N. Andrews , D. Armstrong , et al., “Clinical Practice Guidelines for the Assessment of Uninvestigated Esophageal Dysphagia,” Journal of the Canadian Association of Gastroenterology 1 (2018): 5–19, 10.1093/JCAG/GWX008. 31294391 PMC6487990

[156] P. J. Kahrilas , N. J. Shaheen , M. F. Vaezi , et al., “American Gastroenterological Association Medical Position Statement on the Management of Gastroesophageal Reflux Disease,” Gastroenterology 135, no. 4 (2008): 1383–1391.e5, 10.1053/J.GASTRO.2008.08.045. 18789939

[157] S. Selvanderan , S. Wong , R. Holloway , and P. Kuo , “Dysphagia: Clinical Evaluation and Management,” Internal Medicine Journal 51 (2021): 1021–1027, 10.1111/IMJ.15409. 34278699

[158] English | World Gastroenterology Organisation, accessed July 12, 2024, https://www.worldgastroenterology.org/guidelines/dysphagia/dysphagia‐english.

[159] D. A. Edwards , “Discriminative Information in the Diagnosis of Dysphagia,” Journal of the Royal College of Physicians of London 9, no. 3 (1975): 257–263, 10.1016/s0035-8819(25)03091-0. 1142327 PMC5366509

[160] I. J. Cook , “Diagnostic Evaluation of Dysphagia,” Nature Clinical Practice Gastroenterology & Hepatology 5, no. 7 (2008): 393–403, 10.1038/ncpgasthep1153. 18542115

[161] S. Beg , K. Ragunath , A. Wyman , et al., “Quality Standards in Upper Gastrointestinal Endoscopy: A Position Statement of the British Society of Gastroenterology (BSG) and Association of Upper Gastrointestinal Surgeons of Great Britain and Ireland (AUGIS),” Gut 66, no. 11 (2017): 1886–1899, 10.1136/GUTJNL-2017-314109. 28821598 PMC5739858

[162] S. F. Pasha , R. D. Acosta , V. Chandrasekhara , et al., “The Role of Endoscopy in the Evaluation and Management of Dysphagia,” Gastrointestinal Endoscopy 79, no. 2 (2014): 191–201, 10.1016/J.GIE.2013.07.042. 24332405

[163] I. A. Murray , J. Palmer , C. Waters , et al., “Predictive Value of Symptoms and Demographics in Diagnosing Malignancy or Peptic Stricture,” World Journal of Gastroenterology: WJG 18 (2012): 4357, 10.3748/WJG.V18.I32.4357. 22969199 PMC3436051

[164] S. Varadarajulu , M. A. Eloubeidi , R. S. Patel , et al., “The Yield and the Predictors of Esophageal Pathology When Upper Endoscopy Is Used for the Initial Evaluation of Dysphagia,” Gastrointestinal Endoscopy 61, no. 7 (2005): 804–808, 10.1016/S0016-5107(05)00297-X. 15933679

[165] Overview | Suspected Cancer: Recognition and Referral | Guidance | NICE, accessed July 13, 2024, https://www.nice.org.uk/guidance/NG12.

[166] R. Jones , J. Charlton , R. Latinovic , and M. C. Gulliford , “Alarm Symptoms and Identification of Non‐Cancer Diagnoses in Primary Care: Cohort Study,” BMJ 339, no. aug13 2 (2009): 491–493, 10.1136/BMJ.B3094. PMC2726930 19679615

[167] M. Birk , P. Bauerfeind , P. H. Deprez , et al., “Removal of Foreign Bodies in the Upper Gastrointestinal Tract in Adults: European Society of Gastrointestinal Endoscopy (ESGE) Clinical Guideline,” Endoscopy 48, no. 5 (2016): 489–496, 10.1055/S-0042-100456. 26862844

[168] T. Esfandyari , J. W. Potter , and M. F. Vaezi , “Dysphagia: A Cost Analysis of the Diagnostic Approach,” American Journal of Gastroenterology 97, no. 11 (2002): 2733–2737, 10.1111/J.1572-0241.2002.07061.X. 12425540

[169] S. M. Kavic and M. D. Basson , “Complications of Endoscopy,” American Journal of Surgery 181, no. 4 (2001): 319–332, 10.1016/S0002-9610(01)00589-X. 11438266

[170] C. Krishnamurthy , K. Hilden , K. A. Peterson , N. Mattek , D. G. Adler , and J. C. Fang , “Endoscopic Findings in Patients Presenting With Dysphagia: Analysis of a National Endoscopy Database,” Dysphagia 27, no. 1 (2012): 101–105, 10.1007/S00455-011-9346-0. 21674194 PMC5970000

[171] N. B. Vakil , B. Traxler , and D. Levine , “Dysphagia in Patients With Erosive Esophagitis: Prevalence, Severity, and Response to Proton Pump Inhibitor Treatment,” Clinical Gastroenterology and Hepatology 2 (2004): 665–668, 10.1016/S1542-3565(04)00289-7. 15290658

[172] A. Mari , F. A. Baker , H. S. Ahmad , et al., “The Yield of Endoscopy and Histology in the Evaluation of Esophageal Dysphagia: Two Referral Centers’ Experiences,” Medicina (Buenos Aires) 57, no. 12 (2021): 1336, 10.3390/MEDICINA57121336. PMC8705225 34946281

[173] H. Philpott and R. Sweis , “Hiatus Hernia as a Cause of Dysphagia,” Current Gastroenterology Reports 19, no. 8 (2017): 40, 10.1007/S11894-017-0580-Y. 28730506

[174] T. Kidambi , E. Toto , N. Ho , et al., “Temporal Trends in the Relative Prevalence of Dysphagia Etiologies From 1999‐2009,” World Journal of Gastroenterology: WJG 18 (2012): 4335, 10.3748/WJG.V18.I32.4335. 22969196 PMC3436048

[175] V. N. Mahesh , R. H. Holloway , and N. Q. Nguyen , “Changing Epidemiology of Food Bolus Impaction: Is Eosinophilic Esophagitis to Blame?,” Journal of Gastroenterology and Hepatology 28 (2013): 963–966, 10.1111/JGH.12135. 23425056

[176] H. P. Kim , R. B. Vance , N. J. Shaheen , and E. S. Dellon , “The Prevalence and Diagnostic Utility of Endoscopic Features of Eosinophilic Esophagitis: A Meta‐Analysis,” Clinical Gastroenterology and Hepatology 10, no. 9 (2012): 988–996.e5, 10.1016/J.CGH.2012.04.019. 22610003 PMC3424367

[177] P. Visaggi , B. Barberio , D. Gregori , et al., “Systematic Review With Meta‐Analysis: Artificial Intelligence in the Diagnosis of Oesophageal Diseases,” Alimentary Pharmacology & Therapeutics 55, no. 5 (2022): 528–540, 10.1111/APT.16778. 35098562 PMC9305819

[178] D. Sacks , B. Baxter , B. C. V. Campbell , et al., “Multisociety Consensus Quality Improvement Revised Consensus Statement for Endovascular Therapy of Acute Ischemic Stroke,” International Journal of Stroke 13, no. 6 (2018): 612–632, 10.1177/1747493018778713. 29786478

[179] T. L. Jue , A. C. Storm , M. Naveed , et al., “ASGE Guideline on the Role of Endoscopy in the Management of Benign and Malignant Gastroduodenal Obstruction,” Gastrointestinal Endoscopy 93, no. 2 (2021): 309–322.e4, 10.1016/J.GIE.2020.07.063. 33168194

[180] A. Dhar , H. N. Haboubi , S. E. Attwood , et al., “British Society of Gastroenterology (BSG) and British Society of Paediatric Gastroenterology, Hepatology and Nutrition (BSPGHAN) Joint Consensus Guidelines on the Diagnosis and Management of Eosinophilic Oesophagitis in Children and Adults,” Gut 71 (2022): 1459–1487, 10.1136/GUTJNL-2022-327326. 35606089 PMC9279848

[181] E. S. Dellon , A. B. Muir , D. A. Katzka , et al., “ACG Clinical Guideline: Diagnosis and Management of Eosinophilic Esophagitis,” American Journal of Gastroenterology 120, no. 1 (2025): 31–59, 10.14309/AJG.0000000000003194. 39745304

[182] S. Dellon E , S. Rusin , J. H. Gebhart , et al., “A Clinical Prediction Tool Identifies Cases of Eosinophilic Esophagitis Without Endoscopic Biopsy: A Prospective Study,” American Journal of Gastroenterology 110 (2015): 1347–1354, 10.1038/AJG.2015.239. 26303128 PMC4586067

[183] C. C. Cotton , R. Betancourt , C. Randall , et al., “A Model Using Clinical and Endoscopic Characteristics Identifies Patients at Risk for Eosinophilic Esophagitis According to Updated Diagnostic Guidelines,” Clinical Gastroenterology and Hepatology 19, no. 9 (2021): 1824–1834.e2, 10.1016/J.CGH.2020.06.068. 32634625 PMC7779708

[184] P. Visaggi , G. Del Corso , F. Baiano Svizzero , et al., “Artificial Intelligence Tools for the Diagnosis of Eosinophilic Esophagitis in Adults Reporting Dysphagia: Development, External Validation, and Software Creation for Point‐of‐Care Use,” Journal of Allergy and Clinical Immunology: In Practice 12, no. 4 (2024): 1008–1016.e1, 10.1016/J.JAIP.2023.12.031. 38154556

[185] G. A. Prasad , N. J. Talley , Y. Romero , et al., “Prevalence and Predictive Factors of Eosinophilic Esophagitis in Patients Presenting With Dysphagia: A Prospective Study,” American Journal of Gastroenterology 102, no. 12 (2007): 2627–2632, 10.1111/J.1572-0241.2007.01512.X. 17764492

[186] S. H. Mackenzie , M. Go , B. Chadwick , et al., “Eosinophilic Oesophagitis in Patients Presenting With Dysphagia–A Prospective Analysis,” Alimentary Pharmacology & Therapeutics 28, no. 9 (2008): 1140–1146, 10.1111/J.1365-2036.2008.03795.X. 18624788

[187] I. Hirano , N. Moy , M. G. Heckman , C. S. Thomas , N. Gonsalves , and S. R. Achem , “Endoscopic Assessment of the Oesophageal Features of Eosinophilic Oesophagitis: Validation of a Novel Classification and Grading System,” Gut 62, no. 4 (2013): 489–495, 10.1136/GUTJNL-2011-301817. 22619364

[188] B. D. Van Rhijn , M. J. Warners , W. L. Curvers , et al., “Evaluating the Endoscopic Reference Score for Eosinophilic Esophagitis: Moderate to Substantial Intra‐ and Interobserver Reliability,” Endoscopy 46, no. 12 (2014): 1049–1055, 10.1055/S-0034-1377781. 25208033

[189] P. Visaggi , E. Savarino , G. Sciume , et al., “Eosinophilic Esophagitis: Clinical, Endoscopic, Histologic and Therapeutic Differences and Similarities Between Children and Adults,” Therapeutic Advances in Gastroenterology 14 (2021): 1756284820980860, 10.1177/1756284820980860. 33613690 PMC7871287

[190] J. B. Wechsler , S. M. Bolton , K. Amsden , B. K. Wershil , I. Hirano , and A. F. Kagalwalla , “Eosinophilic Esophagitis Reference Score Accurately Identifies Disease Activity and Treatment Effects in Children,” Clinical Gastroenterology and Hepatology 16, no. 7 (2018): 1056–1063, 10.1016/J.CGH.2017.12.019. 29248734 PMC6003847

[191] E. S. Dellon , C. C. Cotton , J. H. Gebhart , et al., “Accuracy of the Eosinophilic Esophagitis Endoscopic Reference Score in Diagnosis and Determining Response to Treatment,” Clinical Gastroenterology and Hepatology 14, no. 1 (2016): 31–39, 10.1016/j.cgh.2015.08.040. 26404868 PMC4690779

[192] E. S. Dellon , D. A. Katzka , M. H. Collins , et al., “Budesonide Oral Suspension Improves Symptomatic, Endoscopic, and Histologic Parameters Compared With Placebo in Patients With Eosinophilic Esophagitis,” Gastroenterology 152, no. 4 (2017): 776–786.e5, 10.1053/j.gastro.2016.11.021. 27889574

[193] A. J. Lucendo , S. Miehlke , C. Schlag , et al., “Efficacy of Budesonide Orodispersible Tablets as Induction Therapy for Eosinophilic Esophagitis in a Randomized Placebo‐Controlled Trial,” Gastroenterology 157, no. 1 (2019): 74–86.e15, 10.1053/J.GASTRO.2019.03.025. 30922997

[194] M. Kaplan , E. A. Mutlu , S. Jakate , et al., “Endoscopy in Eosinophilic Esophagitis: ‘Feline’ Esophagus and Perforation Risk,” Clinical Gastroenterology and Hepatology 1, no. 6 (2003): 433–437, 10.1016/S1542-3565(03)00222-2. 15017642

[195] P. Visaggi and E. S. Dellon , “The Esophageal Mucosa: Clues to Underlying Pathology,” Gastrointestinal Endoscopy Clinics of North America 35, no. 3 (2025): 503–522, 10.1016/J.GIEC.2024.12.006. 40412987

[196] N. Ahuja , J. Weedon , S. M. Schwarz , R. Sklar , and S. S. Rabinowitz , “Applying the Eosinophilic Esophagitis Endoscopic Reference Scores (EREFS) to Different Aged Children,” Journal of Pediatric Gastroenterology and Nutrition 71, no. 3 (2020): 328–332, 10.1097/MPG.0000000000002788. 32427654

[197] E. S. Dellon , C. A. Liacouras , J. Molina‐Infante , et al., “Updated International Consensus Diagnostic Criteria for Eosinophilic Esophagitis: Proceedings of the AGREE Conference,” Gastroenterology 155 (2018): 1022–1033.e10, 10.1053/J.GASTRO.2018.07.009/ATTACHMENT/64F822E8-1C29-41A5-8D26-FBDB9296201A/MMC1.PDF. 30009819 PMC6174113

[198] C. Römmele , R. Mendel , C. Barrett , et al., “An Artificial Intelligence Algorithm Is Highly Accurate for Detecting Endoscopic Features of Eosinophilic Esophagitis,” Scientific Reports 12, no. 1 (2022): 11115, 10.1038/S41598-022-14605-Z. 35778456 PMC9249895

[199] M. Ribolsi , M. Ghisa , and E. Savarino , “Nonachalasic Esophageal Motor Disorders, From Diagnosis to Therapy,” Expert Review of Gastroenterology & Hepatology 16, no. 3 (2022): 205–216, 10.1080/17474124.2022.2047648. 35220870

[200] M. Ribolsi , G. Andrisani , F. M. Di Matteo , and M. Cicala , “Achalasia, From Diagnosis to Treatment,” Expert Review of Gastroenterology & Hepatology 17, no. 1 (2023): 21–30, 10.1080/17474124.2022.2163236. 36588469

[201] C. P. Gyawali and P. J. Kahrilas , “A Short History of High‐Resolution Esophageal Manometry,” Dysphagia 38, no. 2 (2023): 586–595, 10.1007/S00455-021-10372-7. 34739589 PMC9380033

[202] D. A. Carlson and S. Roman , “Esophageal Provocation Tests: Are They Useful to Improve Diagnostic Yield of High Resolution Manometry?,” Neurogastroenterology and Motility 30, no. 4 (2018): e13321, 10.1111/NMO.13321. 29603510

[203] M. R. Fox and A. J. Bredenoord , “Oesophageal High‐Resolution Manometry: Moving From Research Into Clinical Practice,” Gut 57, no. 3 (2008): 405–423, 10.1136/GUT.2007.127993. 17895358

[204] M. Ribolsi , D. Biasutto , A. Giordano , P. Balestrieri , and M. Cicala , “Role of Esophageal Motility, Acid Reflux, and of Acid Suppression in Nonobstructive Dysphagia,” Journal of Clinical Gastroenterology 52, no. 7 (2018): 607–613, 10.1097/MCG.0000000000000903. 28787356

[205] M. Ribolsi , P. Balestrieri , S. Emerenziani , M. P. L. Guarino , and M. Cicala , “Weak Peristalsis With Large Breaks Is Associated With Higher Acid Exposure and Delayed Reflux Clearance in the Supine Position in GERD Patients,” American Journal of Gastroenterology 109, no. 1 (2014): 46–51, 10.1038/AJG.2013.373. 24189712

[206] S. Kayali , F. Calabrese , A. Pasta , et al., “Effect of Hiatal Hernia and Esophagogastric Junction Morphology on Esophageal Motility: Evidence From High‐Resolution Manometry Studies,” Neurogastroenterology and Motility 36, no. 12 (2024), 10.1111/NMO.14929. 39344398

[207] M. Yang , Z. S. Li , D. F. Chen , et al., “Quantitative Assessment and Characterization of Visceral Hyperalgesia Evoked by Esophageal Balloon Distention and Acid Perfusion in Patients With Functional Heartburn, Nonerosive Reflux Disease, and Erosive Esophagitis,” Clinical Journal of Pain 26, no. 4 (2010): 326–331, 10.1097/AJP.0B013E3181C8FC83. 20393268

[208] K. C. Trimble , A. Pryde , and R. C. Heading , “Lowered Oesophageal Sensory Thresholds in Patients With Symptomatic But Not Excess Gastro‐Oesophageal Reflux: Evidence for a Spectrum of Visceral Sensitivity in GORD,” Gut 37, no. 1 (1995): 7–12, 10.1136/GUT.37.1.7. 7672684 PMC1382759

[209] C. P. Gyawali , R. Yadlapati , R. Fass , et al., “Updates to the Modern Diagnosis of GERD: Lyon Consensus 2.0,” Gut 73, no. 2 (2024): 361–371, 10.1136/GUTJNL-2023-330616. 37734911 PMC10846564

[210] J. P. Kim and P. J. Kahrilas , “How I Approach Dysphagia,” Current Gastroenterology Reports 21, no. 10 (2019): 49, 10.1007/S11894-019-0718-1. 31432250

[211] M. S. Levine and S. E. Rubesin , “Diseases of the Esophagus: Diagnosis With Esophagography,” Radiology 237, no. 2 (2005): 414–427, 10.1148/RADIOL.2372050199. 16170017

[212] W. Blonski , A. Kumar , J. Feldman , and J. E. Richter , “Timed Barium Swallow: Diagnostic Role and Predictive Value in Untreated Achalasia, Esophagogastric Junction Outflow Obstruction, and Non‐Achalasia Dysphagia,” American Journal of Gastroenterology 113, no. 2 (2018): 196–203, 10.1038/AJG.2017.370. 29257145

[213] E. Savarino , S. Bhatia , S. Roman , et al., “Achalasia,” Nature Reviews Disease Primers 8, no. 1 (2022): 28, 10.1038/S41572-022-00356-8. 35513420

[214] M. Andersson , S. Kostic , M. Ruth , et al., “Characteristics of Timed Barium Esophagogram in Newly Diagnosed Idiopathic Achalasia: Clinical and Manometric Correlates,” Acta Radiologica 48, no. 1 (2007): 2–9, 10.1080/02841850601026393. 17325917

[215] L. Fuller , J. E. Huprich , J. Theisen , et al., “Abnormal Esophageal Body Function: Radiographic‐Manometric Correlation,” American Surgeon 65, no. 10 (1999): 911–914, 10.1177/000313489906501003. 10515533

[216] I. El‐Takli , P. O’Brien , and W. G. Paterson , “Clinical Diagnosis of Achalasia: How Reliable Is the Barium x‐Ray?,” Canadian Journal of Gastroenterology 20 (2006): 335–337, 10.1155/2006/193823. 16691299 PMC2659891

[217] S. Sanagapalli , A. Plumb , J. Maynard , R. W. Leong , and R. Sweis , “The Timed Barium Swallow and Its Relationship to Symptoms in Achalasia: Analysis of Surface Area and Emptying Rate,” Neurogastroenterology and Motility 32, no. 12 (2020), 10.1111/NMO.13928. 32578341

[218] W. O. Rohof , D. P. Hirsch , B. F. Kessing , and G. E. Boeckxstaens , “Efficacy of Treatment for Patients With Achalasia Depends on the Distensibility of the Esophagogastric Junction,” Gastroenterology 143, no. 2 (2012): 328–335, 10.1053/J.GASTRO.2012.04.048. 22562023

[219] M. F. Vaezi , M. E. Baker , and J. E. Richter , “Assessment of Esophageal Emptying Post‐Pneumatic Dilation: Use of the Timed Barium Esophagram,” American Journal of Gastroenterology 94 (1999): 1802–1807, 10.1111/J.1572-0241.1999.01209.X. 10406238

[220] R. P. Tatum , G. Shi , M. A. Manka , J. G. Brasseur , R. J. Joehl , and P. J. Kahrilas , “Bolus Transit Assessed by an Esophageal Stress Test in Postfundoplication Dysphagia,” Journal of Surgical Research 91, no. 1 (2000): 56–60, 10.1006/JSRE.2000.5907. 10816350

[221] S. Sanagapalli , A. Plumb , and R. Sweis , “Timed Barium Swallow: Esophageal Stasis Varies Markedly Across Subtypes of Esophagogastric Junction Obstruction,” Neurogastroenterology and Motility 34, no. 3 (2022), 10.1111/NMO.14322. 35072303

[222] M. S. Levine , L. R. Carucci , D. J. DiSantis , et al., “Consensus Statement of Society of Abdominal Radiology Disease‐Focused Panel on Barium Esophagography in Gastroesophageal Reflux Disease,” American Journal of Roentgenology 207, no. 5 (2016): 1009–1015, 10.2214/AJR.16.16323. 27490234

[223] M. Scharitzer , J. Lenglinger , W. Schima , M. Weber , C. Ringhofer , and P. Pokieser , “Comparison of Videofluoroscopy and Impedance Planimetry for the Evaluation of Oesophageal Stenosis: A Retrospective Study,” European Radiology 27, no. 4 (2017): 1760–1767, 10.1007/S00330-016-4516-Y. 27553930 PMC5334389

[224] I. Hirano , J. E. Pandolfino , and G. E. Boeckxstaens , “Functional Lumen Imaging Probe for the Management of Esophageal Disorders: Expert Review From the Clinical Practice Updates Committee of the AGA Institute,” Clinical Gastroenterology and Hepatology 15, no. 3 (2017): 325–334, 10.1016/J.CGH.2016.10.022. 28212976 PMC5757507

[225] J. E. Pandolfino , A. De Ruigh , F. Nicodème , et al., “Distensibility of the Esophagogastric Junction Assessed With the Functional Lumen Imaging Probe (FLIP^TM^) in Achalasia Patients,” Neurogastroenterology and Motility 25 (2013): 496‐e368, 10.1111/nmo.12097. 23413801 PMC3789137

[226] A. L. Holmstrom , R. A. J. Campagna , A. Cirera , et al., “Intraoperative Use of FLIP Is Associated With Clinical Success Following POEM for Achalasia,” Surgical Endoscopy 35 (2021): 3090–3096, 10.1007/s00464-020-07739-6. 32632483

[227] R. A. J. Campagna , D. A. Carlson , E. S. Hungness , et al., “Intraoperative Assessment of Esophageal Motility Using FLIP During Myotomy for Achalasia,” Surgical Endoscopy 34, no. 6 (2020): 2593–2600, 10.1007/s00464-019-07028-x. 31376012 PMC6995437

[228] Z. Lin , P. J. Kahrilas , Y. Xiao , et al., “Functional Luminal Imaging Probe Topography: An Improved Method for Characterizing Esophageal Distensibility in Eosinophilic Esophagitis,” Therapeutic Advances in Gastroenterology 6, no. 2 (2013): 97–107, 10.1177/1756283X12470017. 23503784 PMC3589134

[229] F. Nicodème , I. Hirano , J. Chen , et al., “Esophageal Distensibility as a Measure of Disease Severity in Patients With Eosinophilic Esophagitis,” Clinical Gastroenterology and Hepatology 11, no. 9 (2013): 1101–1107.e1, 10.1016/j.cgh.2013.03.020. 23591279 PMC3790569

[230] F. A. Ponds , A. J. Bredenoord , B. F. Kessing , and A. J. P. M. Smout , “Esophagogastric Junction Distensibility Identifies Achalasia Subgroup With Manometrically Normal Esophagogastric Junction Relaxation,” Neurogastroenterology and Motility 29 (2017): 1–8, 10.1111/nmo.12908. 27458129

[231] J. R. Triggs , D. A. Carlson , C. Beveridge , W. Kou , P. J. Kahrilas , and J. E. Pandolfino , “Functional Luminal Imaging Probe Panometry Identifies Achalasia‐Type Esophagogastric Junction Outflow Obstruction,” Clinical Gastroenterology and Hepatology 18, no. 10 (2020): 2209–2217, 10.1016/j.cgh.2019.11.037. 31778806 PMC7246143

[232] Y. Ichkhanian , O. Sanaei , A. Canakis , et al., “Esophageal Peroral Endoscopic Myotomy (POEM) for Treatment of Esophagogastric Junction Outflow Obstruction: Results From the First Prospective Trial,” Endoscopy International Open 8, no. 9 (2020): E1137–E1143, 10.1055/A-1198-4643. 32904698 PMC7458721

[233] N. K. Ahuja , A. Agnihotri , K. L. Lynch , et al., “Esophageal Distensibility Measurement: Impact on Clinical Management and Procedure Length,” Diseases of the Esophagus 30 (2017): 1–8, 10.1093/dote/dox038. 28575249

[234] D. A. Carlson , C. P. Gyawali , P. J. Kahrilas , et al., “Esophageal Motility Classification Can Be Established at the Time of Endoscopy: A Study Evaluating Real‐Time Functional Luminal Imaging Probe Panometry,” Gastrointestinal Endoscopy 90, no. 6 (2019): 915–923.e1, 10.1016/j.gie.2019.06.039. 31279625 PMC6875629

[235] J. Zarzour , J. Revels , K. B. Rao , et al., “An Update on Pharyngeal Assessment by the Modified Barium Swallow,” Abdominal Radiology (2024): 1–12, 10.1007/S00261-024-04707-9/FIGURES/5. PMC12069153 39648178

[236] M. F. Vaezi and J. E. Richter , “Diagnosis and Management of Achalasia. American College of Gastroenterology Practice Parameter Committee,” American Journal of Gastroenterology 94 (1999): 3406–3412, 10.1111/J.1572-0241.1999.01639.X. 10606295

[237] K. Krishnan , C. Y. Lin , R. Keswani , J. E. Pandolfino , P. J. Kahrilas , and S. Komanduri , “Endoscopic Ultrasound as an Adjunctive Evaluation in Patients With Esophageal Motor Disorders Subtyped by High‐Resolution Manometry,” Neurogastroenterology and Motility 26 (2014): 1172–1178, 10.1111/NMO.12379. 25041229 PMC4331010

[238] P. Kuo , R. H. Holloway , and N. Q. Nguyen , “Current and Future Techniques in the Evaluation of Dysphagia,” Journal of Gastroenterology and Hepatology 27, no. 5 (2012): 873–881, 10.1111/J.1440-1746.2012.07097.X. 22369033

[239] C. Samanci , Y. Onal , and U. Korman , “Videofluoroscopic and Manometric Evaluation of Oropharyngeal and Esophageal Motility Disorders,” Current Medical Imaging Reviews 16, no. 1 (2020): 65–69, 10.2174/157340561501190611154916. 31989895

[240] L. Biggemann , J. Uhlig , N. Gliem , et al., “Assessment of Esophageal Motility Disorders by Real‐Time MRI,” European Journal of Radiology 132 (2020): 109265, 10.1016/J.EJRAD.2020.109265. 33010683

[241] R. Cordier , R. Speyer , M. Martinez , and L. Parsons , “Reliability and Validity of Non‐Instrumental Clinical Assessments for Adults With Oropharyngeal Dysphagia: A Systematic Review,” Journal of Clinical Medicine 12, no. 2 (2023): 721, 10.3390/JCM12020721. 36675650 PMC9861493

[242] R. Speyer , M. Balaguer , E. Cugy , et al., “Expert Consensus on Clinical Decision Making in the Disease Trajectory of Oropharyngeal Dysphagia in Adults: An International Delphi Study,” Journal of Clinical Medicine 12, no. 20 (2023): 6572, 10.3390/JCM12206572. 37892711 PMC10607151

[243] A. Schindler , L. W. J. Baijens , A. Geneid , and N. Pizzorni , “Phoniatricians and Otorhinolaryngologists Approaching Oropharyngeal Dysphagia: An Update on FEES,” European Archives of Oto‐Rhino‐Laryngology 279, no. 6 (2021): 2727–2742, 10.1007/S00405-021-07161-1. 34779927 PMC8591442

[244] B. Labeit , E. Michou , S. Hamdy , et al., “The Assessment of Dysphagia After Stroke: State of the Art and Future Directions,” Lancet Neurology 22, no. 9 (2023): 858–870, 10.1016/S1474-4422(23)00153-9. 37596008

[245] R. Dziewas , M. auf dem Brinke , U. Birkmann , et al., “Safety and Clinical Impact of FEES – Results of the FEES‐Registry,” Neurological Research and Practice 1 (2019): 16, 10.1186/S42466-019-0021-5. 33324882 PMC7650078

[246] T. Warnecke , I. Suttrup , J. B. Schröder , et al., “Levodopa Responsiveness of Dysphagia in Advanced Parkinson’s Disease and Reliability Testing of the FEES‐Levodopa‐Test,” Parkinsonism & Related Disorders 28 (2016): 100–106, 10.1016/J.PARKRELDIS.2016.04.034. 27158122

[247] B. Labeit , E. Berkovich , I. Claus , et al., “Dysphagia for Medication in Parkinson’s Disease,” npj Parkinson's Disease 8, no. 1 (2022): 156, 10.1038/S41531-022-00421-9. PMC9653428 36371409

[248] T. Warnecke , B. Labeit , J. Schroeder , et al., “Neurogenic Dysphagia: Systematic Review and Proposal of a Classification System,” Neurology 96, no. 6 (2021): E876–E889, 10.1212/WNL.0000000000011350. 33318164

[249] A. Westergren , D. Smithard , M. Westergaard , et al., “Convergent and Discriminant Validity of the Minimal Eating Observation Form – Version II: A Cross‐Sectional Study,” BMC Geriatrics 24, no. 1 (2024): 27, 10.1186/S12877-023-04639-X. 38182980 PMC10770885

[250] S. E. Langmore , D. R. Scarborough , L. N. Kelchner , et al., “Tutorial on Clinical Practice for Use of the Fiberoptic Endoscopic Evaluation of Swallowing Procedure With Adult Populations: Part 1,” American Journal of Speech‐Language Pathology 31 (2022): 163–187, 10.1044/2021_AJSLP-20-00348. 34818509

[251] R. Newman , N. Vilardell , P. Clavé , and R. Speyer , “Effect of Bolus Viscosity on the Safety and Efficacy of Swallowing and the Kinematics of the Swallow Response in Patients With Oropharyngeal Dysphagia: White Paper by the European Society for Swallowing Disorders (ESSD),” Dysphagia 31, no. 2 (2016): 232–249, 10.1007/S00455-016-9696-8. 27016216 PMC4929168

[252] E. Boaden , J. Nightingale , C. Bradbury , L. Hives , and R. Georgiou , “Clinical Practice Guidelines for Videofluoroscopic Swallowing Studies: A Systematic Review,” Radiography (London) 26, no. 2 (2020): 154–162, 10.1016/J.RADI.2019.10.011. 32052773

[253] K. Swan , R. Cordier , T. Brown , and R. Speyer , “Psychometric Properties of Visuoperceptual Measures of Videofluoroscopic and Fibre‐Endoscopic Evaluations of Swallowing: A Systematic Review,” Dysphagia 34, no. 1 (2019): 2–33, 10.1007/S00455-018-9918-3. 30019178

[254] L. Baijens , A. Barikroo , and W. Pilz , “Intrarater and Interrater Reliability for Measurements in Videofluoroscopy of Swallowing,” European Journal of Radiology 82, no. 10 (2013): 1683–1695, 10.1016/J.EJRAD.2013.05.009. 23773554

[255] J. Y. Hong , N. K. Hwang , G. Lee , J. S. Park , and Y. J. Jung , “Radiation Safety in Videofluoroscopic Swallowing Study: Systematic Review,” Dysphagia 36, no. 1 (2021): 73–82, 10.1007/S00455-020-10112-3. 32279120

[256] L. F. Giraldo‐Cadavid , L. R. Leal‐Leaño , G. A. Leon‐Basantes , et al., “Accuracy of Endoscopic and Videofluoroscopic Evaluations of Swallowing for Oropharyngeal Dysphagia,” Laryngoscope 127, no. 9 (2017): 2002–2010, 10.1002/LARY.26419. 27859291

[257] P. Potente , A. Buoite Stella , M. Vidotto , et al., “Application of Ultrasonography in Neurogenic Dysphagia: A Systematic Review,” Dysphagia 38, no. 1 (2023): 65–75, 10.1007/S00455-022-10459-9. 35556172 PMC9873712

[258] L. B. Mokkink , C. B. Terwee , D. L. Patrick , et al., “The COSMIN Study Reached International Consensus on Taxonomy, Terminology, and Definitions of Measurement Properties for Health‐Related Patient‐Reported Outcomes,” Journal of Clinical Epidemiology 63, no. 7 (2010): 737–745, 10.1016/J.JCLINEPI.2010.02.006. 20494804

[259] A. A. Timmerman , C. M. G. Meesters , R. Speyer , and L. Anteunis , “Psychometric Qualities of Questionnaires for the Assessment of Otitis Media Impact,” Clinical Otolaryngology 32, no. 6 (2007): 429–439, 10.1111/J.1749-4486.2007.01570.X. 18076428

[260] T. I. Omari , M. Ciucci , K. Gozdzikowska , et al., “High‐Resolution Pharyngeal Manometry and Impedance: Protocols and Metrics‐Recommendations of a High‐Resolution Pharyngeal Manometry International Working Group,” Dysphagia 35, no. 2 (2020): 281–295, 10.1007/S00455-019-10023-Y. 31168756

[261] A. M. Caruso , D. Bommarito , V. Girgenti , et al., “Evaluation of Dysphagia and Inhalation Risk in Neurologically Impaired Children Using Esophageal High‐Resolution Manometry With Swallowing Analysis,” Children (Basel) 9, no. 12 (2022): 1987, 10.3390/CHILDREN9121987. 36553430 PMC9777053

[262] E. M. Diver and J. Regan , “Use of Pharyngeal High‐Resolution Manometry to Evaluate Dysphagia in Adults With Motor Neurone Disease: A Scoping Review,” Dysphagia 37, no. 6 (2022): 1697–1714, 10.1007/S00455-022-10418-4. 35235032 PMC9643180

[263] M. M. Szczesniak , P. I. Wu , J. Maclean , T. I. Omari , and I. J. Cook , “The Critical Importance of Pharyngeal Contractile Forces on the Validity of Intrabolus Pressure as a Predictor of Impaired Pharyngo‐Esophageal Junction Compliance,” Neurogastroenterology and Motility 30, no. 10 (2018), 10.1111/NMO.13374. 29797467

[264] S. P. Rosen , C. A. Jones , M. R. Hoffman , M. A. Knigge , and T. M. McCulloch , “Pressure Abnormalities in Patients With Zenker’s Diverticulum Using Pharyngeal High‐Resolution Manometry,” Laryngoscope Investig Otolaryngol 5, no. 4 (2020): 708–717, 10.1002/LIO2.434. PMC7444802 32864443

[265] C. A. Jones , E. L. Meisner , C. K. Broadfoot , S. P. Rosen , C. R. Samuelsen , and T. M. McCulloch , “Methods for Measuring Swallowing Pressure Variability Using High‐Resolution Manometry,” Frontiers in Applied Mathematics and Statistics 4 (2018): 1–17, 10.3389/FAMS.2018.00023/FULL. PMC6345545 30687729

[266] P. I. Wu , M. M. Szczesniak , T. Omari , et al., “Cricopharyngeal Peroral Endoscopic Myotomy Improves Oropharyngeal Dysphagia in Patients With Parkinson’s Disease,” Endoscopy International Open 9, no. 11 (2021): E1811–E1819, 10.1055/A-1562-7107. 34790549 PMC8589553

[267] T. Omari , C. Cock , P. Wu , et al., “Using High Resolution Manometry Impedance to Diagnose Upper Esophageal Sphincter and Pharyngeal Motor Disorders,” Neurogastroenterology and Motility 35, no. 1 (2023), 10.1111/NMO.14461. 36121685

[268] M. Hollenstein , P. Thwaites , S. Bütikofer , et al., “Pharyngeal Swallowing and Oesophageal Motility During a Solid Meal Test: A Prospective Study in Healthy Volunteers and Patients With Major Motility Disorders,” Lancet Gastroenterology & Hepatology 2 (2017): 644–653, 10.1016/S2468-1253(17)30151-6. 28684261

[269] A. Rengarajan , A. Bolkhir , P. Gor , D. Wang , S. Munigala , and C. P. Gyawali , “Esophagogastric Junction and Esophageal Body Contraction Metrics on High‐Resolution Manometry Predict Esophageal Acid Burden,” Neurogastroenterology and Motility 30, no. 5 (2018): e13267, 10.1111/NMO.13267. 29266647

[270] V. Rangan , N. S. George , F. Khan , et al., “Severity of Ineffective Esophageal Motility Is Associated With Utilization of Skeletal Muscle Relaxant Medications,” Neurogastroenterology and Motility 30, no. 4 (2018): e13235, 10.1111/NMO.13235. 29027725

[271] C. L. Chen , C. H. Yi , T. T. Liu , and W. C. Orr , “Effects of Mosapride on Secondary Peristalsis in Patients With Ineffective Esophageal Motility,” Scandinavian Journal of Gastroenterology 48, no. 12 (2013): 1363–1370, 10.3109/00365521.2013.840856. 24099237

[272] M. Di Stefano , A. Papathanasopoulos , K. Blondeau , et al., “Effect of Buspirone, a 5‐HT1A Receptor Agonist, on Esophageal Motility in Healthy Volunteers,” Diseases of the Esophagus 25, no. 5 (2012): 470–476, 10.1111/J.1442-2050.2011.01275.X. 22050410

[273] G. P. Karamanolis , S. Panopoulos , K. Denaxas , et al., “The 5‐HT1A Receptor Agonist Buspirone Improves Esophageal Motor Function and Symptoms in Systemic Sclerosis: A 4‐Week, Open‐Label Trial,” Arthritis Research and Therapy 18 (2016): 1–6, 10.1186/S13075-016-1094-Y/TABLES/3. 27586891 PMC5009650

[274] N. Aggarwal , P. N. Thota , R. Lopez , and S. Gabbard , “A Randomized Double‐Blind Placebo‐Controlled Crossover‐Style Trial of Buspirone in Functional Dysphagia and Ineffective Esophageal Motility,” Neurogastroenterology and Motility 30, no. 2 (2018), 10.1111/NMO.13213. 28884884

[275] F. Alborzi Avanaki , E. Baghereslami , H. A. Varpaei , et al., “Evaluation of Therapeutic Effect of Buspirone in Improving Dysphagia in Patients With GERD and Ineffective Esophageal Motility: A Randomized Clinical Trial,” Gastroenterology Insights 14 (2023): 1–12, 10.3390/GASTROENT14010001.

[276] W. O. Rohof , R. J. Bennink , A. A. De Ruigh , D. P. Hirsch , A. H. Zwinderman , and G. E. Boeckxstaens , “Effect of Azithromycin on Acid Reflux, Hiatus Hernia and Proximal Acid Pocket in the Postprandial Period,” Gut 61, no. 12 (2012): 1670–1677, 10.1136/GUTJNL-2011-300926. 22267599

[277] A. Agrawal , A. Hila , R. Tutuian , I. Mainie , and D. O. Castell , “Bethanechol Improves Smooth Muscle Function in Patients With Severe Ineffective Esophageal Motility,” Journal of Clinical Gastroenterology 41, no. 4 (2007): 366–370, 10.1097/01.MCG.0000225542.03880.68. 17413603

[278] A. A. Jalil , J. Freeman , and D. O. Castell , “Bethanechol Does Not Improve Dysphagia in Patients With Ineffective Esophageal Motility and Defective Bolus Transit,” supplement, American Journal of Gastroenterology 113, no. Supplement (2018): S231, 10.14309/00000434-201810001-00399.

[279] W. Y. Lei , J. S. Hung , T. T. Liu , C. Yi , and C. Chen , “Influence of Prucalopride on Esophageal Secondary Peristalsis in Reflux Patients With Ineffective Motility,” Journal of Gastroenterology and Hepatology 33, no. 3 (2018): 650–655, 10.1111/JGH.13986. 28898473

[280] M. Fox , D. Menne , B. Stutz , M. Fried , and W. Schwizer , “The Effects of Tegaserod on Oesophageal Function and Bolus Transport in Healthy Volunteers: Studies Using Concurrent High‐Resolution Manometry and Videofluoroscopy,” Alimentary Pharmacology & Therapeutics 24, no. 7 (2006): 1017–1027, 10.1111/J.1365-2036.2006.03090.X. 16984495

[281] B. F. Kessing , A. J. P. M. Smout , R. J. Bennink , N. Kraaijpoel , J. M. Oors , and A. J. Bredenoord , “Prucalopride Decreases Esophageal Acid Exposure and Accelerates Gastric Emptying in Healthy Subjects,” Neurogastroenterology and Motility 26, no. 8 (2014): 1079–1086, 10.1111/NMO.12359. 24891067

[282] R. Tutuian and D. O. Castell , “Review Article: Oesophageal Spasm – Diagnosis and Management,” Alimentary Pharmacology & Therapeutics 23, no. 10 (2006): 1393–1402, 10.1111/J.1365-2036.2006.02917.X. 16669954

[283] I. Kristo , K. Schwameis , S. Maschke , et al., “Phenotypes of Jackhammer Esophagus in Patients With Typical Symptoms of Gastroesophageal Reflux Disease Responsive to Proton Pump Inhibitors,” Scientific Reports 8, no. 1 (2018): 9949, 10.1038/S41598-018-27756-9. 29967357 PMC6028385

[284] S. Philonenko , S. Roman , F. Zerbib , et al., “Jackhammer Esophagus: Clinical Presentation, Manometric Diagnosis, and Therapeutic Results‐Results From a Multicenter French Cohort,” Neurogastroenterology and Motility 32, no. 11 (2020), 10.1111/NMO.13918. 32510747

[285] N. Viazis , G. Karamanolis , E. Vienna , and D. G. Karamanolis , “Selective‐Serotonin Reuptake Inhibitors for the Treatment of Hypersensitive Esophagus,” Therapeutic Advances in Gastroenterology 4, no. 5 (2011): 295–300, 10.1177/1756283X11409279. 21922028 PMC3165206

[286] H. Lee , J. H. Kim , B. H. Min , et al., “Efficacy of Venlafaxine for Symptomatic Relief in Young Adult Patients With Functional Chest Pain: A Randomized, Double‐Blind, Placebo‐Controlled, Crossover Trial,” American Journal of Gastroenterology 105, no. 7 (2010): 1504–1512, 10.1038/AJG.2010.82. 20332772

[287] R. E. Clouse , P. J. Lustman , T. C. Eckert , D. M. Ferney , and L. S. Griffith , “Low‐Dose Trazodone for Symptomatic Patients With Esophageal Contraction Abnormalities: A Double‐Blind, Placebo‐Controlled Trial,” Gastroenterology 92, no. 4 (1987): 1027–1036, 10.1016/0016-5085(87)90979-6. 3549420

[288] M. Pimentel , G. G. Bonorris , E. J. Chow , and H. C. Lin , “Peppermint Oil Improves the Manometric Findings in Diffuse Esophageal Spasm,” Journal of Clinical Gastroenterology 33, no. 1 (2001): 27–31, 10.1097/00004836-200107000-00007. 11418786

[289] B. L. A. M. Weusten , M. Barret , A. J. Bredenoord , et al., “Endoscopic Management of Gastrointestinal Motility Disorders – Part 2: European Society of Gastrointestinal Endoscopy (ESGE) Guideline,” Endoscopy 52, no. 7 (2020): 600–614, 10.1055/A-1171-3174. 32462649

[290] B. L. A. M. Weusten , M. Barret , A. J. Bredenoord , et al., “Endoscopic Management of Gastrointestinal Motility Disorders – Part 1: European Society of Gastrointestinal Endoscopy (ESGE) Guideline,” Endoscopy 52, no. 6 (2020): 498–515, 10.1055/A-1160-5549. 32375192

[291] D. Schupack , D. A. Katzka , D. M. Geno , and K. Ravi , “The Clinical Significance of Esophagogastric Junction Outflow Obstruction and Hypercontractile Esophagus in High Resolution Esophageal Manometry,” Neurogastroenterology and Motility 29, no. 10 (2017): 1–9, 10.1111/NMO.13105. 28544670

[292] F. Mion , S. Marjoux , F. Subtil , et al., “Botulinum Toxin for the Treatment of Hypercontractile Esophagus: Results of a Double‐Blind Randomized Sham‐Controlled Study,” Neurogastroenterology and Motility 31, no. 5 (2019): e13587, 10.1111/NMO.13587. 30974039

[293] T. Vanuytsel , R. Bisschops , R. Farré , et al., “Botulinum Toxin Reduces Dysphagia in Patients With Nonachalasia Primary Esophageal Motility Disorders,” Clinical Gastroenterology and Hepatology 11 (2013): 1115–1121.e2, 10.1016/J.CGH.2013.03.021. 23591282

[294] L. Bernardot , S. Roman , M. Barret , et al., “Efficacy of Per‐Oral Endoscopic Myotomy for the Treatment of Non‐Achalasia Esophageal Motor Disorders,” Surgical Endoscopy 34, no. 12 (2020): 5508–5515, 10.1007/S00464-019-07348-Y/METRICS. 31932930

[295] M. A. Khashab , P. Familiari , P. V. Draganov , et al., “Peroral Endoscopic Myotomy Is Effective and Safe in Non‐Achalasia Esophageal Motility Disorders: An International Multicenter Study,” Endoscopy International Open 6, no. 8 (2018): E1031–E1036, 10.1055/A-0625-6288. 30105290 PMC6086680

[296] H. Inoue , H. Shiwaku , K. Iwakiri , et al., “Clinical Practice Guidelines for Peroral Endoscopic Myotomy,” Digestive Endoscopy 30, no. 5 (2018): 563–579, 10.1111/DEN.13239. 30022514

[297] M. A. Khan , V. Kumbhari , S. Ngamruengphong , et al., “Is POEM the Answer for Management of Spastic Esophageal Disorders? A Systematic Review and Meta‐Analysis,” Digestive Diseases and Sciences 62, no. 1 (2017): 35–44, 10.1007/S10620-016-4373-1. 27858325

[298] J. D. Irving , W. J. Owen , J. Linsell , M. McCullagh , A. Keightley , and A. Anggiansah , “Management of Diffuse Esophageal Spasm With Balloon Dilatation,” Gastrointestinal Radiology 17, no. 1 (1992): 189–192, 10.1007/BF01888544/METRICS. 1612298

[299] P. Visaggi , M. Ghisa , C. G. Del , et al., “Chicago Classification v4.0 Protocol Improves Specificity and Accuracy of Diagnosis of Oesophagogastric Junction Outflow Obstruction,” Alimentary Pharmacology & Therapeutics 56, no. 4 (2022): 606–613, 10.1111/APT.17101. 35751633 PMC9544646

[300] J. E. Richter and S. B. Clayton , “Diagnosis and Management of Esophagogastric Junction Outflow Obstruction,” American Journal of Gastroenterology 114, no. 4 (2019): 544–547, 10.14309/AJG.0000000000000100. 30848733

[301] R. Salvador , L. Provenzano , G. Nezi , et al., “Laparoscopic Heller‐Dor Is an Effective Treatment for Esophageal‐Gastric Junction Outflow Obstruction,” Journal of Gastrointestinal Surgery 25, no. 9 (2021): 2201–2207, 10.1007/S11605-021-05021-1. 33959877 PMC8484249

[302] N. de Bortoli , P. Visaggi , R. Penagini , et al., “The 1st EoETALY Consensus on the Diagnosis and Management of Eosinophilic Esophagitis–Current Treatment and Monitoring,” Digestive and Liver Disease 56, no. 7 (2024): 1173–1184, 10.1016/J.DLD.2024.02.020/ATTACHMENT/923AA5C5-F43C-45BE-AB47-BDC941A7973B/MMC1.DOCX. 38521670

[303] A. Babaei , A. Szabo , S. D. Yorio , and B. T. Massey , “Pressure Exposure and Catheter Impingement Affect the Recorded Pressure in the Manoscan 360^TM^ System,” Neurogastroenterology and Motility 30, no. 8 (2018), 10.1111/NMO.13329. PMC6129441 29520927

[304] C. Beveridge and K. Lynch , “Diagnosis and Management of Esophagogastric Junction Outflow Obstruction,” Gastroenterology & Hepatology 16 (2020): 131, 10.14309/00000434-900000000-99796. 34035712 PMC8132699

[305] C. Maradey‐Romero and R. Fass , “Antidepressants for Functional Esophageal Disorders: Evidence‐ or Eminence‐Based Medicine?,” Clinical Gastroenterology and Hepatology 13 (2015): 260–262, 10.1016/j.cgh.2014.09.044. 25283580

[306] P. L. Peghini , P. O. Katz , and D. O. Castell , “Imipramine Decreases Oesophageal Pain Perception in Human Male Volunteers,” Gut 42, no. 6 (1998): 807–813, 10.1136/GUT.42.6.807. 9691919 PMC1727141

[307] P. W. Weijenborg , H. S. de Schepper , A. J. P. M. Smout , and A. J. Bredenoord , “Effects of Antidepressants in Patients With Functional Esophageal Disorders or Gastroesophageal Reflux Disease: A Systematic Review,” Clinical Gastroenterology and Hepatology 13, no. 2 (2015): 251–259.e1, 10.1016/j.cgh.2014.06.025. 24997325

[308] M. Fox , C. Barr , S. Nolan , M. Lomer , A. Anggiansah , and T. Wong , “The Effects of Dietary Fat and Calorie Density on Esophageal Acid Exposure and Reflux Symptoms,” Clinical Gastroenterology and Hepatology 5, no. 4 (2007): 439–444.e1, 10.1016/J.CGH.2006.12.013. 17363334

[309] K. Ravi , L. Friesen , R. Issaka , P. J. Kahrilas , and J. E. Pandolfino , “Long‐Term Outcomes of Patients With Normal or Minor Motor Function Abnormalities Detected by High‐Resolution Esophageal Manometry,” Clinical Gastroenterology and Hepatology 13, no. 8 (2015): 1416–1423, 10.1016/J.CGH.2015.02.046. 25771245 PMC4510014

[310] V. J. Colon , M. A. Young , and F. C. Ramirez , “The Short‐ and Long‐Term Efficacy of Empirical Esophageal Dilation in Patients With Nonobstructive Dysphagia: A Prospective, Randomized Study,” American Journal of Gastroenterology 95, no. 4 (2000): 910–913, 10.1111/J.1572-0241.2000.01928.X. 10763936

[311] A. Gasiorowska , T. Navarro‐Rodriguez , R. Dickman , et al., “Clinical Trial: The Effect of Johrei on Symptoms of Patients With Functional Chest Pain,” Alimentary Pharmacology & Therapeutics 29, no. 1 (2009): 126–134, 10.1111/J.1365-2036.2008.03859.X. 18945261

[312] K. F. Kopel and M. Quinn , “Hypnotherapy Treatment for Dysphagia,” International Journal of Clinical and Experimental Hypnosis 44, no. 2 (1996): 101–105, 10.1080/00207149608416073. 8871337

[313] A. R. Kumar and P. O. Katz , “Functional Esophageal Disorders: A Review of Diagnosis and Management,” Expert Review of Gastroenterology & Hepatology 7, no. 5 (2013): 453–461, 10.1586/17474124.2013.811028. 23899284

[314] D. H. Vasant , E. Michou , N. O’Leary , A. Vail , S. Mistry , and S. Hamdy , “Pharyngeal Electrical Stimulation in Dysphagia Poststroke: A Prospective, Randomized Single‐Blinded Interventional Study,” Neurorehabilitation and Neural Repair 30, no. 9 (2016): 866–875, 10.1177/1545968316639129. 27053641

[315] A. Guillén‐Solà , S. M. Messagi , S. N. Bofill , E. Duarte , M. C. Barrera , and E. Marco , “Respiratory Muscle Strength Training and Neuromuscular Electrical Stimulation in Subacute Dysphagic Stroke Patients: A Randomized Controlled Trial,” Clinical Rehabilitation 31, no. 6 (2017): 761–771, 10.1177/0269215516652446. 27271373

[316] A. B. Hammad , E. A. Elhamrawy , H. Abdel‐Tawab , et al., “Transcranial Magnetic Stimulation Versus Transcutaneous Neuromuscular Electrical Stimulation in Post Stroke Dysphagia: A Clinical Randomized Controlled Trial,” Journal of Stroke and Cerebrovascular Diseases 31, no. 8 (2022): 106554, 10.1016/j.jstrokecerebrovasdis.2022.106554. 35691184

[317] E. M. Khedr , K. O. Mohamed , R. K. Soliman , A. M. M. Hassan , and J. C. Rothwell , “The Effect of High‐Frequency Repetitive Transcranial Magnetic Stimulation on Advancing Parkinson’s Disease With Dysphagia: Double Blind Randomized Clinical Trial,” Neurorehabilitation and Neural Repair 33, no. 6 (2019): 442–452, 10.1177/1545968319847968. 31072214

[318] Y. H. Ahn , H. J. Sohn , J. S. Park , et al., “Effect of Bihemispheric Anodal Transcranial Direct Current Stimulation for Dysphagia in Chronic Stroke Patients: A Randomized Clinical Trial,” Journal of Rehabilitation Medicine 49, no. 1 (2017): 30–35, 10.2340/16501977-2170. 27904911

[319] V. Arreola , O. Ortega , D. Álvarez‐Berdugo , et al., “Effect of Transcutaneous Electrical Stimulation in Chronic Poststroke Patients With Oropharyngeal Dysphagia: 1‐Year Results of a Randomized Controlled Trial,” Neurorehabilitation and Neural Repair 35, no. 9 (2021): 778–789, 10.1177/15459683211023187. 34137329

[320] L. Van Der Molen , M. A. Van Rossum , L. M. Burkhead , L. E. Smeele , C. R. N. Rasch , and F. J. M. Hilgers , “A Randomized Preventive Rehabilitation Trial in Advanced Head and Neck Cancer Patients Treated With Chemoradiotherapy: Feasibility, Compliance, and Short‐Term Effects,” Dysphagia 26, no. 2 (2011): 155–170, 10.1007/S00455-010-9288-Y. 20623305 PMC3098976

[321] B. N. Krekeler , J. Yee , A. Kurosu , et al., “Effects of Device‐Facilitated Lingual Strengthening Therapy on Dysphagia Related Outcomes in Patients Post‐Stroke: A Randomized Controlled Trial,” Dysphagia 38, no. 6 (2023): 1551–1567, 10.1007/S00455-023-10583-0. 37195518 PMC10615659

[322] R. Shaker , C. Easterling , M. Kern , et al., “Rehabilitation of Swallowing by Exercise in Tube‐Fed Patients With Pharyngeal Dysphagia Secondary to Abnormal UES Opening,” Gastroenterology 122, no. 5 (2002): 1314–1321, 10.1053/gast.2002.32999. 11984518

[323] G. D. Carnaby , L. LaGorio , S. Silliman , and M. Crary , “Exercise‐Based Swallowing Intervention (McNeill Dysphagia Therapy) With Adjunctive NMES to Treat Dysphagia Post‐Stroke: A Double‐Blind Placebo‐Controlled Trial,” Journal of Oral Rehabilitation 47, no. 4 (2020): 501–510, 10.1111/JOOR.12928. 31880338 PMC7067660

[324] X. Wei , F. Yu , M. Dai , et al., “Change in Excitability of Cortical Projection After Modified Catheter Balloon Dilatation Therapy in Brainstem Stroke Patients With Dysphagia: A Prospective Controlled Study,” Dysphagia 32, no. 5 (2017): 645–656, 10.1007/S00455-017-9810-6. 28550485 PMC5608794

[325] E. Walsh , A. J. Krause , M. Greytak , et al., “Laryngeal Recalibration Therapy Improves Laryngopharyngeal Symptoms in Patients With Suspected Laryngopharyngeal Reflux Disease,” American Journal of Gastroenterology 119, no. 11 (2024): 2198–2205, 10.14309/AJG.0000000000002839. 38656937 PMC11534515

[326] M. Hägg , L. Tibbling , and T. Franzén , “Effect of IQoro(R) Training in Hiatal Hernia Patients With Misdirected Swallowing and Esophageal Retention Symptoms,” Acta Oto‐Laryngologica 135, no. 7 (2015): 635–639, 10.3109/00016489.2015.1016185. 25963055

[327] R. Speyer , R. Cordier , A. L. Sutt , et al., “Behavioural Interventions in People With Oropharyngeal Dysphagia: A Systematic Review and Meta‐Analysis of Randomised Clinical Trials,” Journal of Clinical Medicine 11, no. 3 (2022): 685, 10.3390/JCM11030685/S1. 35160137 PMC8836405

[328] D. G. Pfister , S. Spencer , D. Adelstein , et al., “Head and Neck Cancers, Version 2.2020, NCCN Clinical Practice Guidelines in Oncology,” Journal of the National Comprehensive Cancer Network 18, no. 7 (2020): 873–898, 10.6004/JNCCN.2020.0031. 32634781

[329] T. Y. Seiwert and E. E. W. Cohen , “State‐of‐the‐Art Management of Locally Advanced Head and Neck Cancer,” British Journal of Cancer 92 (2005): 1341–1348, 10.1038/sj.bjc.6602510. 15846296 PMC2361996

[330] D. V. Albers , A. Kondo , W. M. Bernardo , et al., “Endoscopic Versus Surgical Approach in the Treatment of Zenker’s Diverticulum: Systematic Review and Meta‐Analysis,” Endoscopy International Open 4, no. 6 (2016): E678–E686, 10.1055/S-0042-106203. 27556078 PMC4993875

[331] S. Elkholy , M. El‐Sherbiny , R. Delano‐Alonso , et al., “Peroral Endoscopic Myotomy as Treatment for Zenker’s Diverticulum (Z‐POEM): A Multi‐Center International Study,” Esophagus 18, no. 3 (2021): 693–699, 10.1007/S10388-020-00809-7. 33387150

[332] A. P. Marston , F. J. Maldonado , K. Ravi , J. L. Kasperbauer , and D. C. Ekbom , “Treatment of Oropharyngeal Dysphagia Secondary to Idiopathic Cricopharyngeal Bar: Surgical Cricopharyngeal Muscle Myotomy Versus Dilation,” American Journal of Otolaryngology 37, no. 6 (2016): 507–512, 10.1016/J.AMJOTO.2016.07.006. 27522437

[333] O. Gilheaney , P. Kerr , S. Béchet , and M. Walshe , “Effectiveness of Endoscopic Cricopharyngeal Myotomy in Adults With Neurological Disease: Systematic Review,” Journal of Laryngology & Otology 130, no. 12 (2016): 1077–1085, 10.1017/S0022215116008975. 27938463

[334] M. Pitman and P. Weissbrod , “Endoscopic CO_2_ Laser Cricopharyngeal Myotomy,” Laryngoscope 119, no. 1 (2009): 45–53, 10.1002/LARY.20032. 19117309

[335] S. S. Al Ghamdi , M. Bejjani , O. V. Hernández Mondragón , et al., “Peroral Endoscopic Myotomy for Management of Cricopharyngeal Bars (CP‐POEM): A Retrospective Evaluation,” Endoscopy 54, no. 5 (2022): 498–502, 10.1055/A-1646-1151. 34710910

[336] Z. Liu , J. Cheng , C. Tan , H. Liu , and D. Han , “Pharyngeal Cavity Electrical Stimulation‐Assisted Swallowing for Post‐Stroke Dysphagia: A Systematic Review and Meta‐Analysis of Randomized Controlled Studies,” Dysphagia 39, no. 4 (2023): 541–551, 10.1007/S00455-023-10644-4/METRICS. 38117313

[337] I. Cheng , A. Sasegbon , and S. Hamdy , “Dysphagia Treatments in Parkinson’s Disease: A Systematic Review and Meta‐Analysis,” Neurogastroenterology and Motility 35, no. 8 (2023): e14517, 10.1111/NMO.14517. 36546568

[338] I. Diéguez‐Pérez and R. Leirós‐Rodríguez , “Effectiveness of Different Application Parameters of Neuromuscular Electrical Stimulation for the Treatment of Dysphagia After a Stroke: A Systematic Review,” Journal of Clinical Medicine 9, no. 8 (2020): 2618, 10.3390/JCM9082618. 32806675 PMC7463982

[339] H. Yu , K. Takahashi , L. Bloom , S. D. Quaynor , and T. Xie , “Effect of Deep Brain Stimulation on Swallowing Function: A Systematic Review,” Frontiers in Neurology 11 (2020): 534003, 10.3389/FNEUR.2020.00547/BIBTEX. PMC7380112 32765388

[340] Y. Sun , X. Chen , J. Qiao , et al., “Effects of Transcutaneous Neuromuscular Electrical Stimulation on Swallowing Disorders: A Systematic Review and Meta‐Analysis,” American Journal of Physical Medicine & Rehabilitation 99, no. 8 (2020): 701–711, 10.1097/PHM.0000000000001397. 32209833 PMC7343179

[341] W. J. Kim , C. Rosselin , B. Amatya , P. Hafezi , and F. Khan , “Repetitive Transcranial Magnetic Stimulation for Management of Post‐Stroke Impairments: An Overview of Systematic Reviews,” Journal of Rehabilitation Medicine 52 (2020): 1–10, 10.2340/16501977-2637. 31922207

[342] C. F. Chiang , M. T. Lin , M. Y. Hsiao , Y. C. Yeh , Y. C. Liang , and T. G. Wang , “Comparative Efficacy of Noninvasive Neurostimulation Therapies for Acute and Subacute Poststroke Dysphagia: A Systematic Review and Network Meta‐Analysis,” Archives of Physical Medicine and Rehabilitation 100, no. 4 (2019): 739–750.e4, 10.1016/j.apmr.2018.09.117. 30352222

[343] X. Liao , G. Xing , Z. Guo , et al., “Repetitive Transcranial Magnetic Stimulation as an Alternative Therapy for Dysphagia After Stroke: A Systematic Review and Meta‐Analysis,” Clinical Rehabilitation 31, no. 3 (2016): 289–298, 10.1177/0269215516644771. 27113337

[344] S. N. Yang , S. B. Pyun , H. J. Kim , H. S. Ahn , and B. J. Rhyu , “Effectiveness of Non‐Invasive Brain Stimulation in Dysphagia Subsequent to Stroke: A Systemic Review and Meta‐Analysis,” Dysphagia 30, no. 4 (2015): 383–391, 10.1007/S00455-015-9619-0/METRICS. 25917018

[345] Y. W. Chen , K. H. Chang , H. C. Chen , W. M. Liang , Y. H. Wang , and Y. N. Lin , “The Effects of Surface Neuromuscular Electrical Stimulation on Post‐Stroke Dysphagia: A Systemic Review and Meta‐Analysis,” Clinical Rehabilitation 30, no. 1 (2015): 24–35, 10.1177/0269215515571681. 25697453

[346] R. Speyer , A. L. Sutt , L. Bergström , et al., “Neurostimulation in People With Oropharyngeal Dysphagia: A Systematic Review and Meta‐Analyses of Randomised Controlled Trials‐Part I: Pharyngeal and Neuromuscular Electrical Stimulation,” Journal of Clinical Medicine 11, no. 3 (2022): 776, 10.3390/JCM11030776. 35160228 PMC8836998

