# Correction to “Esophageal and Oropharyngeal Dysphagia: Clinical Recommendations From the United European Gastroenterology and European Society for Neurogastroenterology and Motility”

**DOI:** 10.1002/ueg2.70088

**Published:** 2025-07-31

Mari A, Calabrese F, Pasta A, Lorenzon G, Weusten B, Keller J, Visaggi P, Roman S, Marabotto E, Dickman R, Serra J, De Bortoli N, Iovino P, Pohl D, Dumitrascu D, Ribolsi M, Barber C, Bor S, Fox M, Sweis R, Lorenzo‐Zuniga V, Akyuz F, Ghisa M, Celebi A, Shibli F, Dziewas R, Kalkan IH, Tack J, Clavé P, Carrion S, Cheng I, Tomsen N, Ortega O, Rubio SM, Pizzorni N, Michou E, Regan J, Hamdy S, Rommel N, Scharitzer M, Ekberg O, Schindler A, Speyer R, Gillman A, Zerbib F, and Savarino, EV. (2025), Esophageal and Oropharyngeal Dysphagia: Clinical Recommendations From the United European Gastroenterology and European Society for Neurogastroenterology and Motility. *United European Gastroenterol J*, 13: 855–901. https://doi.org/10.1002/ueg2.70062.

Rami Sweis’s name was misspelled in the original publication and has now been corrected.

We apologize for this error.

