# Supplementary material for: Esophageal and Oropharyngeal Dysphagia: Clinical Recommendations From the United European Gastroenterology and European Society for Neurogastroenterology and Motility
Source: United European Gastroenterol J. 2025 Jun 21;13(6):855–901. doi: 10.1002/ueg2.70062 (PMC12269739; doi:10.1002/ueg2.70062)
Supplement: Supplementary file 1 — Table S1 [file UEG2-13-855-s001.docx]

**Supplementary Table 1.** Overview of the clinical questions addressed in the guideline, along with search terms, filters, and study types included in the evidence assessment for each statement

| **Section and number** | **Question** | **Search terms** | **Filters** | **Types of studies** |
| --- | --- | --- | --- | --- |
| **Statement 1.1** | What is the definition of esophageal dysphagia? | - Esophageal dysphagia - Dysphagia - Definition | 1930 articles found on pubmed and 6 on cochrane. After manual search through titles/abstracts by AM, only 7 papers found to be relevant. | Systematic reviews with/without meta-analysis, randomized/non-randomized clinical trials, cohort studies, observational studies, abstract communications (major conferences 2020-2023). I excluded low quality evidence, such as expert opinion articles, case reports, case series or preclinical studies. |
| **Statement 1.2** | What is the current definition of oropharyngeal dysphagia and what symptoms and signs are currently included in this definition? | - Dysphagia - Oro-pharyngeal dysphagia - Oropharyngeal dysphagia - Sign - Symptom | Not applicable | Systematic reviews with/without meta-analysis, non-randomized clinical trials, cohort studies, observational studies, book chapters |
| **Statement 1.3** | What is the prevalence of esophageal dysphagia? | - Esophageal dysphagia - Dysphagia - Prevalence | 210 articles found on Pubmed and 6 on Cochrane. After manual search through titles/abstracts by AM, only 28 papers found to be relevant. | Systematic reviews with/without meta-analysis, randomized/non-randomized clinical trials, cohort studies, observational studies, abstract communications (major conferences 2020-2023).I excluded low quality evidence, such as expert opinion articles, case reports, case series or preclinical studies. |
| **Statement 1.4** | What is the prevalence of oropharyngeal dysphagia, and does it differ across target populations? | - dysphagia; - oro-pharyngeal dysphagia; - oropharyngeal dysphagia; - prevalence; - epidemiology | adult patients | Systematic reviews with/without meta-analysis, randomized/non-randomized clinical trials, cohort studies, observational studies |
| **Statement 1.5** | How does oropharyngeal dysphagia impact the health economics of the healthcare system? | - Deglutition OR deglutition disorders - Oropharynx/abnormalities - Oropharynx/pathology - Oropharynx/physiopathology - Oropharynx/therapy - Economics - Health Resources - Tertiary Care Centers/economics - Rehabilitation Centers/economics - Medicare/economics - Food, Formulated/ economics | Animal studies were excluded | Systematic reviews with/without meta-analysis, cohort studies and observational studies |
| **Statement 2.1** | What systemic diseases can lead to esophageal and/or oropharyngeal dysphagia? | - Esophageal dysphagia - Oropharyngeal dysphagia - Systemic diseases - Dysphagia etiology | Peer-reviewed articles  Publications from 2010 to the present  Human studies only  Exclude case reports and preclinical studies | Systematic reviews with/without meta-analysis, Randomized/non-randomized clinical trials, Cohort studies, Observational studies, Clinical guidelines |
| **Statement 2.2** | Are Major Motility disorders at HRM definitely explain dysphagia? | - Vomiting - Nausea - Esophageal manometry - High resolution esophageal manometry - Esophageal motility disorders - Achalasia - Nutcracker esophagus, jackhammer esophagus, esophageal hypomotility; - esophageal motility disorder [mesh] - dysphagia | Not applicable | Systematic reviews with/without meta-analysis, randomized/non-randomized clinical trials, cohort studies, observational studies, abstract communications (major conferences 2020-2022). I excluded low quality evidence, such as expert opinion articles, case reports, case series or preclinical studies. |
| **Statement 2.3** | Does spastic (premature) esophageal contractions contribute to the symptoms of esophageal dysphagia? | - Dysphagia - Esophageal spasms, - Diffuse esophageal spasm - Premature esophageal contraction - Esophageal dysphagia. | Not applicable | Systematic reviews with/without meta-analysis, randomized/non-randomized clinical trials, cohort studies, observational studies. I try to exclude low quality evidence, such as expert opinion articles, case reports, case series or preclinical studies. |
| **Statement 2.4** | Does hypercontractile contraction contribute to the symptoms of esophageal dysphagia? | - Dysphagia - Esophageal dysphagia - Esophageal hypercontractile contraction - Hypercontractile esophagus   Jackhammer esophagus. | Not applicable | Systematic reviews with/without meta-analysis, randomized/non-randomized clinical trials, cohort studies, observational studies. I try to exclude low quality evidence, such as expert opinion articles, case reports, case series or preclinical studies. |
| **Statement 2.5** | Is IEM a mechanism underlying symptoms of esophageal dysphagia? | - Vomiting - Nausea - High resolution esophageal manometry - Esophageal motility disorders - Esophageal motility disorder | Not applicable | Systematic reviews with/without meta-analysis, randomized/non-randomized clinical trials, cohort studies, observational studies, abstract communications (major conferences 2020-2022) |
| **Statement 2.6** | Does Esophageal hypersensitivity contribute to the symptoms of esophageal dysphagia? | - Esophageal hypersensitivity - Dysphagia mechanisms - Esophageal dysphagia - Functional dysphagia - Bolus stasis - Esophageal motor disorders | Not applicable | Systematic reviews with/without meta-analysis, randomized/non-randomized clinical trials, cohort studies, observational studies, abstract communications (major conferences 2020-2023). I was not able to exclude low quality evidence, such as expert opinion articles, case reports or case series |
| **Statement 2.7** | Does decreased EGJ distensibility contribute to the symptoms of esophageal dysphagia? | - Esophageal dysphagia - Esophagogastric junction-outflow obstruction - Functional luminal imaging probe (FLIP) - EGJ distensibility | Not applicable | Systematic reviews with/without meta-analysis, randomized/non-randomized clinical trials, cohort studies, observational studies. I try to exclude low quality evidence, such as expert opinion articles, case reports, case series or preclinical studies. |
| **Statement 2.8** | Does impaired LES relaxation contribute to the symptoms of esophageal dysphagia? | - Dysphagia - Lower esophageal sphincter - Esophageal dysphagia - Esophageal achalasia - Esophageal gastro junction outflow obstruction | Not applicable | Systematic reviews with/without meta-analysis, randomized/non-randomized clinical trials, cohort studies, observational studies. I try to exclude low quality evidence, such as expert opinion articles, case reports, case series or preclinical studies. |
| **Statement 2.9** | Are there any consequences of esophageal motility disorders? | - Chronic esophageal dysmotility - Epiphrenic diverticulum - Candida esophagitis - Esophageal strictures - Management strategies - POEM - Surgery - Nutrition management - Antifungal therapy | Peer-reviewed articles, clinical guidelines, and reviews.  Excluded non-peer-reviewed sources, case reports, and articles older than 10 years unless deemed seminal. | Systematic reviews with/without meta-analysis, randomized/non-randomized clinical trials, cohort studies, observational studies, clinical guidelines. Excluded low quality evidence such as expert opinion articles, single case reports, case series, or preclinical studies. |
| **Statement 2.10** | What are the main phenotypes of patients with OD? | - Oropharyngeal dysphagia - Swallowing disorders - Phenotypes: patients, aging, older people, neurogenic, stroke, neurodegenerative disease, Parkinson, Alzheimer, dementia, traumatic brain injury, neurodegenerative disease, multiple sclerosis, amyotrophic lateral sclerosis, cerebral palsy, neuromuscular disorders, myositis, myasthenia gravis, head and neck cancer | Not applicable | Systematic reviews with/without meta-analysis, randomized/non-randomized clinical trials, cohort studies, observational studies |
| **Statement 2.11** | What biomechanical, neurophysiological, and structural alterations contribute/are the cause of oropharyngeal dysphagia? | - Dysphagia - Deglutition - Oropharyngeal dysphagia - Oro-pharyngeal dysphagia   AND   - Biomechanics - Videofluoroscopy - Physiology - Pathophysiology - Swallowing - Neurophysiology - Neurogenic - Pharyngeal - Head and neck cancer - Radiotherapy, surgery, laryngectomy - Zenker’s diverticulum - Osteophytes - Stricture | Human study | Reviews, systematic reviews with/without meta-analysis, cohort studies, observational studies, experimental studies and clinical trials (of relevance). |
| **Statement 2.12** | What are the main complications of oropharyngeal dysphagia and their prevalence? | - Dysphagia - Deglutition - Oropharyngeal dysphagia   AND   - Respiratory complications - Aspiration - Sarcopenia - Malnutrition OR undernutrition - Dehydration - Prevalence. | Not applicable | Reviews, systematic reviews with/without meta-analysis, cohort studies, observational studies. |
| **Statement 2.13** | Is there an overlap between esophageal dysphagia and anxiety/depression? | - Esophageal dysphagia - Dysphagia - Anxiety and/or depression | 96 articles found on Pubmed and 0 on Cochrane. After manual search through titles/abstracts by AM, only 9 papers found to be relevant. | Systematic reviews with/without meta-analysis, randomized/non-randomized clinical trials, cohort studies, observational studies, abstract communications (major conferences 2020-2023). I excluded low quality evidence, such as expert opinion articles, case reports, case series or preclinical studies. |
| **Statement 3.1** | Is medical history sufficient to differentiate between esophageal and oropharyngeal dysphagia? | - Esophageal dysphagia - Oropharyngeal dysphagia - Clinical, anamnesis, and/or history taking | 64 articles found on Pubmed and 0 on Cochrane. After manual search through titles/abstracts by GL, ES and AM, 22 papers found to be relevant. | Systematic reviews with/without meta-analysis, randomized/non-randomized clinical trials, cohort studies, observational studies, abstract communications (major conferences 2020-2023). I excluded low quality evidence, such as expert opinion articles, case reports, case series or preclinical studies. |
| **Statement 3.2** | When endoscopy should be performed in esophageal dysphagia evaluation? | - Deglutition disorders - Dysphagia - Endoscopy - Diagnosis | - Humans - Adolescent - Adult - Middle-age - Young adult - Adult - 80 and over | Systematic reviews with/without meta-analysis, randomized/non-randomized clinical trials, cohort studies, observational studies |
| **Statement 3.3** | What are the endoscopic findings at EGD that may explain a structural origin in esophageal dysphagia? | - Deglutition disorders - Dysphagia - Endoscopy - Diagnosis | - Humans - Adolescent - Adult - Middle-age - Young adult - Adult - 80 and over | Systematic reviews with/without meta-analysis, randomized/non-randomized clinical trials, cohort studies, observational studies |
| **Statement 3.4** | When are esophageal biopsies needed in patients esophageal dysphagia? | - Vomiting - Nausea - High resolution esophageal manometry - Esophageal motility disorders | Not applicable | Systematic reviews with/without meta-analysis, randomized/non-randomized clinical trials, cohort studies, observational studies, abstract communications (major conferences 2020-2022). I try to exclude low quality evidence, such as expert opinion articles, case reports, case series or preclinical studies. |
| **Statement 3.5** | What are the EoE typical endoscopic findings at EGD? | - Vomiting - Nausea - High resolution esophageal manometry - Esophageal motility disorders | Not applicable | Systematic reviews with/without meta-analysis, randomized/non-randomized clinical trials, cohort studies, observational studies, abstract communications (major conferences 2020-2022). I try to exclude low quality evidence, such as expert opinion articles, case reports, case series or preclinical studies. |
| **Statement 3.6** | Where is the place of HRM assessment in esophageal dysphagia? | - Dysphagia - high resolution esophageal manometry - esophageal motility disorders - Chicago Criteria | Not applicable | Systematic reviews with/without meta-analysis, cohort studies, observational prospective/retrospective studies |
| **Statement 3.7** | Where is the place of pH and impedance measurements in esophageal dysphagia? | - Dysphagia - pH-metry - impedance-pH - GERD | Not applicable | Systematic cohort studies, observational prospective/retrospective studies |
| **Statement 3.8** | When barium esophagogram is needed in the evaluation of esophageal dysphagia? | - Esophageal dysphagia - barium esophagogram - esophageal stricture - esophageal motor disorders | Peer-reviewed articles, clinical guidelines, and reviews. Excluded non-peer-reviewed sources, case reports, and articles older than 10 years unless deemed seminal. | Systematic reviews with/without meta-analysis, randomized/non-randomized clinical trials, cohort studies, observational studies, clinical guidelines. Excluded low quality evidence such as expert opinion articles, single case reports, case series, or preclinical studies. |
| **Statement 3.9** | When EndoFlip is useful in the evaluation of esophageal dysphagia? | - EndoFLIP and dysphagia - Achalasia - Eosinophil esophagitis - Esophageal motility disorders | Not applicable | Systematic reviews with/without meta-analysis, randomized/non-randomized clinical trials, cohort studies, observational studies, abstract communications. I try to exclude low quality evidence, such as expert opinion articles, case reports, case series or preclinical studies. |
| **Statement 3.10** | When Imaging studies are needed in the evaluation of dysphagia? | - Dysphagia - Imaging studies - Barium swallow - CT - MRI - Esophageal motility studies | Peer-reviewed articles, clinical guidelines, and reviews.  Excluded non-peer-reviewed sources, case reports, and articles older than 10 years unless deemed seminal. | Systematic reviews with/without meta-analysis, randomized/non-randomized clinical trials, cohort studies, observational studies, clinical guidelines. Excluded low quality evidence such as expert opinion articles, single case reports, case series, or preclinical studies. |
| **Statement 3.11** | Is clinical swallow evaluation sufficient to characterize oropharyngeal dysphagia? | - Deglutition OR Deglutition Disorders   AND   - Assessment - Screen - Measure - Tool - Instrument - Evaluation | Oropharyngeal dysphagia  Adult populations  Exclusion criteria: Esophageal dysphagia, Pediatric populations | Systematic reviews with/without meta-analysis, randomized/non-randomized clinical trials, cohort studies, observational studies, abstract communications (major conferences 2020-2022)*, ESSD white papers |
| **Statement 3.12** | When is FEES assessment indicated in oropharyngeal dysphagia evaluation? | - Oropharyngeal dysphagia   AND   - FEES - Fiberoptic endoscopic evaluation swallowing - Flexible endoscopic evaluation swallowing   AND   - Indication - indicated | Inclusion criteria: Oropharyngeal dysphagia, Adult populations  Exclusion criteria: Esophageal dysphagia, Pediatric populations | Systematic reviews with/without meta-analysis, randomized/non-randomized clinical trials, cohort studies, observational studies, abstract communications (major conferences 2020-2022). |
| **Statement 3.13** | When and what type of radiological imaging is needed in the evaluation of oropharyngeal dysfunction? | - Deglutition - Dysphagia - Oropharyngeal   AND   - Fluoroscopy - Barium - Diagnostic Imaging | ESSD white papers + reference checking  Exclusion criteria: Esophageal dysphagia, Pediatric dysphagia | Systematic reviews with/without meta-analysis, randomized/non-randomized clinical trials, cohort studies, observational studies, abstract communications (major conferences 2020-2022)*, ESSD white papers |
| **Statement 3.14** | When is high resolution manometry assessment indicated in oropharyngeal dysphagia? | - Manometr* AND Pharyn* - High resolution manometry - Manometry   AND   - Assessment - Evaluation   AND   - Oropharyngeal - Oro-pharyngeal - Pharyngeal | Inclusion: pharyngeal manometry, oropharyngeal dysphagia, adult population, healthy participants  Exclusion: oesophageal dysphagia, infant & paediatric dysphagia, esophageal manometry – esophageal dysphagia | Systematic reviews with/without meta-analysis, randomized/non-randomized clinical trials, cohort studies, observational studies, abstract communications (major conferences 2020-2022). Low quality evidence, such as expert opinion articles, case reports, case series or preclinical studies have been excluded |
| **Statement 4.1** | Which is the medical management of ineffective motility and aperistalsis? | - Hypotensive esophageal motility - Esophageal aperistalsis - Ineffective esophageal motility - Medical management of esophageal motility disorders | Peer-reviewed articles, clinical guidelines, and reviews.  Excluded non-peer-reviewed sources, case reports, and articles older than 10 years unless deemed seminal. | Systematic reviews with/without meta-analysis, randomized/non-randomized clinical trials, cohort studies, observational studies, clinical guidelines. Excluded low quality evidence such as expert opinion articles, single case reports, case series, or preclinical studies. |
| **Statement 4.2** | Which is the medical management of esophageal hypercontraction or esophageal spasm? | - Esophageal hypercontraction - Esophageal spasm - Medical management - Proton pump inhibitors - Antidepressants - Peppermint oil | Peer-reviewed articles, clinical guidelines, and reviews.  Excluded non-peer-reviewed sources, case reports, and articles older than 10 years unless deemed seminal. | Systematic reviews with/without meta-analysis, randomized/non-randomized clinical trials, cohort studies, observational studies, clinical guidelines. Excluded low quality evidence such as expert opinion articles, single case reports, case series, or preclinical studies. |
| **Statement 4.3** | What is the effectiveness of endoscopic management of hypercontractile esophageal motility disorders? | - Esophageal spasms - Diffuse esophageal spasm - Hypercontractile esophageal motility - Hypercontractile esophagus - Peroral endoscopic myotomy - Botulin toxin injection - Ballon dilatation | Not applicable | Meta-analysis, randomized/non-randomized clinical trials, cohort studies, observational studies |
| **Statement 4.4** | What are the medical and endoscopic management strategies for esophagogastric junction outflow obstruction (EGJOO) apart from achalasia? | - Esophagogastric junction outflow obstruction (EGJOO) - Medical management - Endoscopic management - Structural EGJOO - Functional EGJOO | Peer-reviewed articles, clinical guidelines, and reviews.  Excluded non-peer-reviewed sources, case reports, and articles older than 10 years unless deemed seminal. | Systematic reviews with/without meta-analysis, randomized/non-randomized clinical trials, cohort studies, observational studies, clinical guidelines. Excluded low quality evidence such as expert opinion articles, single case reports, case series, or preclinical studies. |
| **Statement 4.5** | What are the medical management options for dysphagia in the context of normal motility? | - Functional dysphagia - Normal esophageal motility - Medical management - Antidepressants - Neuromodulators | Peer-reviewed articles, clinical guidelines, and reviews.  Excluded non-peer-reviewed sources, case reports, and articles older than 10 years unless deemed seminal. | Systematic reviews with/without meta-analysis, randomized/non-randomized clinical trials, cohort studies, observational studies, clinical guidelines. Excluded low quality evidence such as expert opinion articles, single case reports, case series, or preclinical studies. |
| **Statement 4.6** | What is the role of alternative therapies (Psychiatric, Cognitive Behavioral Therapy (CBT), Behavioral Modification, etc.) for functional dysphagia? | - Functional dysphagia - Alternative therapies - Psychiatric therapies - Cognitive behavioural therapy - Behavioural modification - Complementary therapies | Peer-reviewed articles, clinical guidelines, and reviews.  Excluded non-peer-reviewed sources, case reports, and articles older than 10 years unless deemed seminal. | Systematic reviews with/without meta-analysis, randomized/non-randomized clinical trials, cohort studies, observational studies, clinical guidelines. Excluded low quality evidence such as expert opinion articles, single case reports, case series, or preclinical studies. |
| **Statement 4.7** | Is there a role for swallow therapy in the management of oropharyngeal dysphagia? When? | - Deglutition disorders - Deglutition - Swallow - Dysphag* - Rehabilitat* - Therap* - Prehabilitat* - Pre-habilit*   AND   - Oropharyngeal - Oro-pharyngeal - Pharyngeal - Oral | Inclusion: Oropharyngeal dysphagia, adult populations  Exclusion: oesophageal dysphagia, infant & paediatric dysphagia, healthy participants | Given the high number of articles retrieved (2727), only randomised control trials were included, with the following exclusion criteria added: Excluded: pilot RCTs, free water protocols, compensatory strategies, oral hygiene interventions, pharmacological interventions. Included RCTs: 47 |
| **Statement 4.8** | Is there a role for surgical treatment in the management of oropharyngeal dysphagia? When? | - Cricopharyngeal muscle - Cricopharyngeus - Cricopharyngeal - Cricopharyngeal spasm - Cricopharyngeal dysfunction - Cricopharyngeal achalasia - Cricopharyngeal bar - Cricopharyngeal sphincter - Upper esophageal sphincter - UES   AND   - Myotomy - Endoscopic myotomy - Laster myotomy - Open myotomy - Zenker OR Zenker’s diverticulum AND surgery OR endoscopic treatment OR endoscopy OR divetuculopexy | Only articles in English were included | Systematic reviews with/without meta-analysis, randomized/non-randomized clinical trials, cohort studies, observational studies, abstract communications (major conferences 2020-2022) |
| **Statement 4.9** | What is the evidence for neuromodulatory treatments in Oropharyngeal Dysphagia and which patient group could be targeted with neuromodulation? | - Deglutition - Deglutition Disorders - AND - Neurostim* - Brain stim* - Neuromodulation* - Neuromuscular Electrical Stim* - Transcranial stimulation* - Current stimulation* | Inclusion criteria: Oropharyngeal dysphagia, Adult populations,  Exclusion criteria: Esophageal dysphagia, Pediatric populations | Systematic reviews with/without meta-analysis |

**Abbreviations:** CBT: Cognitive Behavioral Therapy; CT: Computed Tomography; EGD: Esophagogastroduodenoscopy; EGJ: Esophagogastric Junction; EGJOO: Esophagogastric Junction Outflow Obstruction; EoE: Eosinophilic Esophagitis; FEES: Fiberoptic Endoscopic Evaluation of Swallowing; FLIP: Functional Luminal Imaging Probe; GERD: Gastroesophageal Reflux Disease; GL: Author initials; HRM: High Resolution Manometry; IEM: Ineffective Esophageal Motility; LES: Lower Esophageal Sphincter; MRI: Magnetic Resonance Imaging; OD: Oropharyngeal Dysphagia; POEM: Peroral Endoscopic Myotomy; RCT: Randomized Controlled Trial; RCTs: Randomized Controlled Trials
